# Time-resolved transcriptome analysis and lipid pathway reconstruction of the oleaginous green microalga *Monoraphidium neglectum* reveal a model for triacylglycerol and lipid hyperaccumulation

**DOI:** 10.1186/s13068-017-0882-1

**Published:** 2017-08-14

**Authors:** Daniel Jaeger, Anika Winkler, Jan H. Mussgnug, Jörn Kalinowski, Alexander Goesmann, Olaf Kruse

**Affiliations:** 1 Algae Biotechnology and Bioenergy, Faculty of Biology, Center for Biotechnology (CeBiTec), Bielefeld University, 33615 Bielefeld, Germany; 2 Microbial Genomics and Biotechnology, Center for Biotechnology (CeBiTec), Bielefeld University, 33615 Bielefeld, Germany; 3 Bioinformatics and Systems Biology, Justus-Liebig-Universität, 35392 Gießen, Germany; 4 Algae Biotechnology and Bioenergy, Faculty of Biology, Center for Biotechnology (CeBiTec), Bielefeld University, Universitaetsstrasse 27, 33615 Bielefeld, Germany

**Keywords:** *Monoraphidium neglectum*, mRNA-seq, Biodiesel, Lipid metabolism, Nitrogen starvation, TAG accumulation, Pathway analysis, Fatty acid, Lipase, Central carbon metabolism

## Abstract

**Background:**

Oleaginous microalgae are promising production hosts for the sustainable generation of lipid-based bioproducts and as bioenergy carriers such as biodiesel. Transcriptomics of the lipid accumulation phase, triggered efficiently by nitrogen starvation, is a valuable approach for the identification of gene targets for metabolic engineering.

**Results:**

An explorative analysis of the detailed transcriptional response to different stages of nitrogen availability was performed in the oleaginous green alga *Monoraphidium neglectum*. Transcript data were correlated with metabolic data for cellular contents of starch and of different lipid fractions. A pronounced transcriptional down-regulation of photosynthesis became apparent in response to nitrogen starvation, whereas glucose catabolism was found to be up-regulated. An in-depth reconstruction and analysis of the pathways for glycerolipid, central carbon, and starch metabolism revealed that distinct transcriptional changes were generally found only for specific steps within a metabolic pathway. In addition to pathway analyses, the transcript data were also used to refine the current genome annotation. The transcriptome data were integrated into a database and complemented with data for other microalgae which were also subjected to nitrogen starvation. It is available at https://tdbmn.cebitec.uni-bielefeld.de.

**Conclusions:**

Based on the transcriptional responses to different stages of nitrogen availability, a model for triacylglycerol and lipid hyperaccumulation is proposed, which involves transcriptional induction of thioesterases, differential regulation of lipases, and a re-routing of the central carbon metabolism. Over-expression of distinct thioesterases was identified to be a potential strategy to increase the oleaginous phenotype of *M. neglectum*, and furthermore specific lipases were identified as potential targets for future metabolic engineering approaches.

**Electronic supplementary material:**

The online version of this article (doi:10.1186/s13068-017-0882-1) contains supplementary material, which is available to authorized users.

## Background

The production of bulk bio-commodities in a sustainable way is a key target of many biotechnological processes. Owing to their phototrophic growth characteristics, microalgae have been considered to be promising candidates for the production of biofuels such as biodiesel, bioethanol, biogas, or biohydrogen (H_2_), as well as of high-value products such as terpenoids, polyunsaturated fatty acids (FAs), or recombinant proteins [1–3]. In the context of biodiesel production however, microalgal lipid productivity needs to be improved for overall economic feasibility [3–5]. A valuable strategy to reach this goal is metabolic engineering [6, 7]. However, the current understanding of the algal lipid metabolism is still incomplete, although it has been progressively investigated in the model green alga *Chlamydomonas reinhardtii* [8]. For the design of rational metabolic engineering strategies, a valuable approach is to follow the cell’s endogenous regulation of carbon partitioning under conditions of high lipid and especially triacylglycerol (TAG) productivity. TAG accumulation in microalgae is efficiently induced by nitrogen starvation (−N) [9] and transcriptome studies yield an initial overview of pathway regulation, which is scalable to the single-gene level. Therefore, the investigation of the transcriptome profiles during nitrogen limitation is an appropriate strategy for the identification of gene targets.

Previous transcriptome studies with the aim to investigate the molecular mechanisms of TAG accumulation under −N conditions were performed for the green algae *C. reinhardtii* [10–15], *Chlorella sorokiniana* [16], *Neochloris oleoabundans* [17], and *Neodesmus* sp. [18], as well as the diatom *Phaeodactylum tricornutum* [19] and the eustigmatophyceae *Nannochloropsis oceanica* [20] and *Nannochloropsis gaditana* [21]. These studies have shown a transcriptional induction of nitrogen assimilation [10, 11, 17, 19], of pyruvate kinase [13, 17, 19, 20, 22] indicating a redirection of carbon flow towards pyruvate generation, of the tricarboxylic acid cycle [17, 19, 20], and of a subset of diacylglycerol acyltransferases which are key enzymes for TAG synthesis [10, 12, 19, 20]. These changes were accompanied by a general transcriptional repression of the cellular processes photosynthesis [10, 11, 17, 19, 20, 23], translation (ribosomes) [11, 17, 20, 23], and gluconeogenesis [10, 19, 20].

Today, the most detailed time course experiments were performed for the model green alga *C. reinhardtii* [12–14, 22], where the nutrient starvation phase was investigated in great detail at several time points and in different mutants, such as the starchless strain *sta6* [14]. However, in those works, the reverse phase of nutrient resupply after the starvation phase, triggering degradation of TAGs [24] and other storage compounds such as starch [25], has not been investigated so far. Furthermore, *C. reinhardtii* is not considered as the optimal choice for large-scale biofuel production [26]. At the same time, dynamic transcript changes in other chlorophyceae have not been investigated in great detail. In this regard, only single time point analyses are available for the oleaginous chlorophyceae *N. oleoabundans* (11 days of –N) [17] and *Neodesmus* sp. (a single pool of samples from 3 and 6 h of –N) [18], as well as for the squalene-rich chlorophyceae *Botryosphaerella sudetica* (3 days of –N) [23]. Although more extensive transcriptome data were acquired for *C. sorokiniana*, such that nitrogen limitation was investigated in both heterotrophic and autotrophic conditions, only one time point from each condition was sampled and biological replicates were not performed [16].

The chlorophyceae *Monoraphidium neglectum* was recently identified as a promising strain for the sustainable production of lipid-derived bioproducts [27]. The species was demonstrated to exhibit robust growth characteristics and a neutral lipid productivity of 52 ± 6 mg L^−1^ day^−1^ under autotrophic conditions, which is four times the productivity of the model chlorophyceae *C. reinhardtii* [27]. When exposed to –N treatment, neutral lipids accumulated to ca. 33% of the total dry biomass [28], with fatty acid profiles being well suited for biodiesel production [27]. Furthermore, the genome has recently been sequenced [27], and it was shown that genetic transformation and stable recombinant protein expression are possible [29]. We therefore chose this promising species as a target for a time-resolved investigation of its transcriptome profiles under nutrient replete and nutrient starvation conditions. In contrast to transcriptome studies with *C. reinhardtii* [10–14, 22], we applied fully autotrophic conditions with excess CO_2_, therefore more closely representing the conditions for sustainable biofuel production. Our goals for the transcriptome analysis were to (a) elucidate the physiological pathways important for cellular lipid turnover processes and (b) identify potential bottlenecks for metabolic pathway engineering to further improve the capacity for neutral lipid production with this microalga. Towards this end, we performed a time course experiment consisting of a −N phase and a subsequent +N resupply step. The −N phase was subdivided into two stages, an early −N stage (e−N), characterized by increased starch production, and late −N stage (l−N), characterized by increased lipid production. The third stage investigated in this work constituted the N resupply treatment (r+N). During each of the stages, multiple samples were taken and analyzed by mRNA sequencing (mRNA-seq). With this experimental setup, lipid accumulation (−N) and lipid degradation (+N) were both analyzed at the transcriptional level for the first time by mRNA-seq in the context of microalgal lipid accumulation. As an additional benefit of the transcriptome sequencing, we used the extensive data to improve the currently available genome annotation with the aim to facilitate future genetic engineering approaches.

## Results

### Overview of experiments

One major goal of this study was the identification of gene targets that could be promising for subsequent genetic engineering approaches with the aim to increase the microalgal triacylglycerol (TAG) accumulation. Towards this end, we applied transcriptomics of *M. neglectum* under alternating phases of nitrogen (N) availability, as microalgal TAG accumulation is efficiently induced by −N treatment [9].

**Fig. 1:**
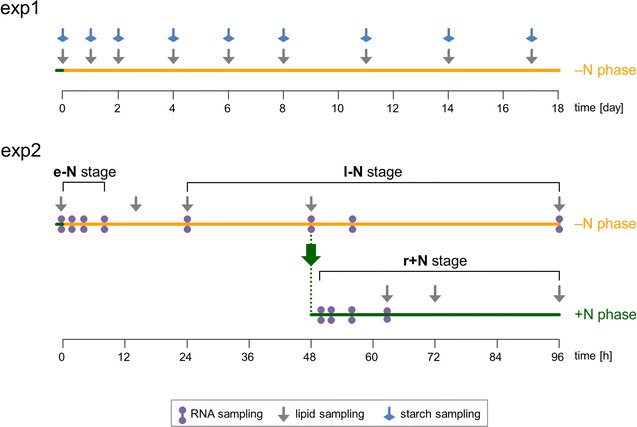
Experimental design to elucidate the transcriptional mechanism of microalgal TAG accumulation, triggered by nitrogen starvation, in *M. neglectum*. In a first experiment, the dynamic of cellular starch and lipid accumulation in response to −N treatment by *M. neglectum* was investigated in a long-term −N experiment. The individual sampling time points for starch and lipid determination are depicted; the corresponding metabolic data are shown in Fig. 2. In order to elucidate the transcriptional mechanisms correlating with starch and lipid accumulation, a second −N experiment was performed. In this experiment, an additional N resupply treatment was included, in order to induce the end of cellular quiescence and to consequently trigger the reversal of storage compound accumulation. By this combined treatment of alternating phases of N availability, the transcriptional program for both accumulation and degradation of TAGs was monitored, which facilitated the identification of central transcriptional responses underlying microalgal TAG accumulation. As starch accumulation precedes TAG accumulation under −N conditions, the −N phase was subdivided into two stages: e−N = starch accumulation stage and l−N = TAG and lipid hyperaccumulation stage. Upon N resupply, storage compounds were degraded, i.e., r+N = TAG degradation stage. The individual sampling time points for RNA and lipid isolation are depicted; the corresponding metabolic data are shown in Additional file 1: Figure S1

As a preparatory step, a long-term −N experiment of 17 days of autotrophic −N conditions was performed, in which the dynamics of starch, TAG, and total lipid accumulation in *M. neglectum* were identified (Fig. 1, exp1). As a result, the −N phase could be subdivided into an early stage of starch accumulation (e−N stage), and a subsequent, late stage where TAG and total lipid levels increased (l−N stage).

For transcriptome analysis, we decided to further extend the scope by not only investigating the conditions of TAG accumulation, but also including conditions of TAG degradation, in order to obtain a profound understanding of the transcriptional regulation of the TAG metabolism in *M. neglectum*. TAG accumulation can be reversed by resupplementation of N [24, 30, 31]. Therefore, the experiment one was repeated, and an additional N resupply treatment (r+N stage) after 48 h of −N conditions was included (Fig. 1, exp2).

### Characterization of the cellular response of *M. neglectum* to nitrogen starvation to define the timing of starch, TAG, and total lipid accumulation

Starch and TAGs are the major storage compounds in chlorophyceae [32, 33]. For the model microalga *C. reinhardtii*, a biphasic pattern of starch and TAG accumulation under –N conditions has been described, with starch accumulation preceding TAG accumulation [34]. In order to investigate how far this pattern also applies to *M. neglectum*, the dynamics of starch and lipid accumulation were determined during a long-term −N trial (Fig. 1).

**Fig. 2:**
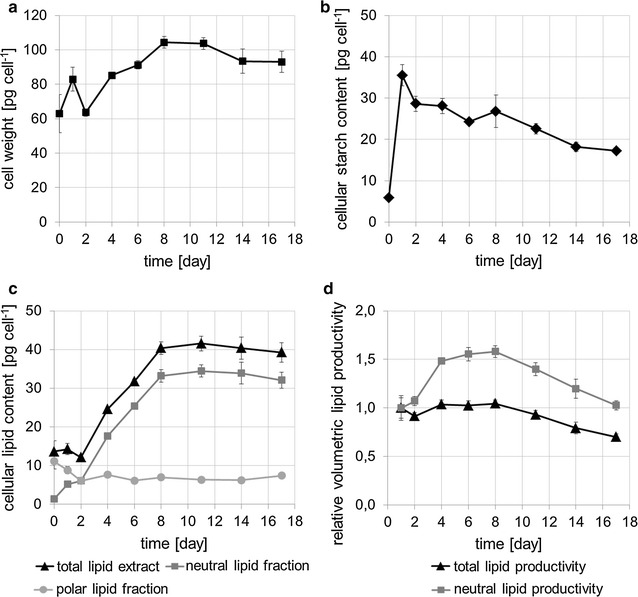
Cell weight, starch, and lipid profiles of *M. neglectum* during 17 days of nitrogen starvation (exp1). **a** Cell weight during the time course of −N treatment. **b** Starch content per cell, determined by enzymatic reaction of solubilized starch with amyloglucosidase, hexokinase, and glucose-6-phosphate dehydrogenase measuring NADPH production. **c** Gravimetrically determined total (*black*, *triangles*), neutral (*dark gray*, *squares*), and polar (*light gray*, *circles*) lipid content per cell. **d** Relative volumetric total (*black*, *triangles*) and neutral (*gray*, *squares*) lipid productivities. In **a**–**d**, mean values and standard errors (*n* = 3) are shown

Removal of N resulted in cessation of cell doubling after approximately 2 days of starvation, while biomass concentrations continuously increased from initially 0.236 ± 0.024 g L^−1^ SE (standard error, *n* = 3) to 1.583 ± 0.06 g L^−1^ SE after 11 days of −N conditions. Increasing biomass concentrations and cessation of cell doubling indicate storage compound accumulation and translated into an increase in cell weight (Fig. 2a).

The cellular starch level was found to peak at day 1 and slowly declined afterwards (Fig. 2b). Such a pattern was also described for *Chlorella zofingiensis* [35], but is in contrast to *C. reinhardtii*, for which a continuously increasing starch level was reported, however with a decreasing slope after 2 days of −N conditions [34, 36]. The neutral lipid content of *M. neglectum* increased almost linearly until day 8, after which it remained approximately constant (Fig. 2c).

During the first 2 days of −N cultivation, no net increase of the cellular total lipid content was observed (Fig. 2c, black line). Interestingly, a clear accumulation of the neutral lipid fraction was observed (Fig. 2c, dark gray line), whereas the fraction of polar lipids decreased (Fig. 2c, light gray line). These opposing tendencies indicate that during this period TAG accumulation was connected to acyl chain recycling from membrane lipids into the TAG pool. At the same time, we noticed that FA synthesis remained active, because the volumetric total lipid content increased from 51 ± 7 mg L^−1^ SE (day 0) to 155 ± 5 mg L^−1^ SE (day 2) (data not shown). This did, however, not translate into an increase of the cellular total lipid content, because the cell concentration concomitantly increased from 3.7 ± 0.2 × 10^6^ cells mL^−1^ SE (day 0) to 12.9 ± 0.9 × 10^6^ cells mL^−1^ SE (day 2) (data not shown). In the following days, both the total lipid levels as well as the neutral lipid levels strongly increased, while the polar lipid amount remained approximately constant (Fig. 2c). This indicates that during this period TAG accumulation was directly fueled by de novo synthesis of cellular FAs. Additionally, a metabolic state allowing for total lipid hyperaccumulation had been established. Importantly, starch degradation apparently was not a major contributor to the total lipid accumulation in *M. neglectum*, because cellular starch levels declined by only ~4 pg from day 2 to day 8, while the total lipid content increased by ~28 pg in the same period of time (Fig. 2b, c).

Interestingly, in this period the cell weight even increased by ~40 pg (Fig. 2a), therefore by more than the sum of the lipid and starch levels. Accordingly, the observed increase of the cell weight cannot be attributed solely to lipid accumulation, indicating that additional carbon sinks must exist, possibly associated with cell wall components.

The optimal harvesting time for maximum volumetric neutral lipid production was found to be between day 4 and day 8, while the volumetric productivity of total lipids remained constant until day 8 (Fig. 2d). Note that low cell concentrations of approximately 4 million cells mL^−1^ were used for inoculation, in order to ensure optimal light penetration and consequently rapid neutral lipid accumulation [27, 37]. Low cell concentrations, however, might not be biotechnologically most relevant. Accordingly, we did not calculate absolute volumetric neutral lipid productivity values, as those would certainly be misleading by underestimating the amounts expected from cultures with higher cell concentrations.

In summary, the long-term −N experiment revealed a similar biphasic pattern of starch accumulation preceding TAG accumulation in *M. neglectum*, as has been reported for other chlorophyceae [34–36, 38].

### Design of the transcriptome experiment and data evaluation

For the transcriptome study, we extended the analysis from −N conditions to include N resupply conditions, in order to analyze both the accumulation and the degradation phase of the TAGs. Towards this end, the −N experiment was repeated and after 48 h an aliquot of N-starved cells was resuspended in N-containing media, while the remaining cells were kept under continuous −N conditions (Fig. 1, exp2). As expected, the N resupply treatment resulted in decreased levels of both the neutral lipid and the total lipid contents (Additional file 1: Figure S1), indicating TAG and FA degradation, respectively.

RNA samples for deep mRNA sequencing were taken from all three stages of N availability (e−N, l−N, and r+N stages), constituting a total of twelve time points (Fig. 1, exp2, purple circles). The first sample was taken immediately prior to the −N treatment and is referred to as the reference time point N_0 representing the transcriptome from exponential growth conditions. For the e−N stage, three samples were taken, after 2, 4, and 8 h of −N conditions, referred to as N_2, N_4, and N_8, respectively. These time points were chosen to represent the transcriptional basis of starch accumulation and of acyl chain recycling from membrane lipids for early TAG accumulation. For the l−N stage, four samples were taken, after 24, 48, 56, and 96 h of −N conditions, referred to as N_24, N_48, N_56, and N_96, respectively. This was to elucidate the transcriptional basis for TAG and total lipid hyperaccumulation. The relatively large time interval was chosen to cover putatively different phases of TAG accumulation, as well as to differentiate between transient and stable transcriptional responses. For the r+N stage, another four samples were taken, after 2, 4, 8, and 14 h of N resupply conditions, referred to as R_2, R_4, R_8, and R_14, respectively. It was accordingly possible to identify different timings of transcript changes triggering the end of cellular quiescence and to comprehensively investigate the reversal of storage compound accumulation.

As the transcriptome of *M. neglectum* was sequenced for the first time, we aimed to acquire a great sequencing depth, in order to also obtain read support for genes with low expression, allowing accurate reconstruction of transcript models for the majority of genes. To reach this goal, we limited the number of samples being sequenced, so that more reads per individual sample could be obtained. Since we expected that transcript abundance changes were rather accurately monitored by mRNA-seq because mRNA-seq was often reported to exhibit low technical variability [39, 40] and to correlate well with RT-qPCR data [15, 19, 20, 41–44], sequencing replicates were not performed. Sequencing replicates have also been omitted in other studies investigating transcriptome changes of microalgae subjected to −N conditions [15, 21, 23]. To mitigate biological variance, a pool of equal amounts of total RNA from two biological replicates was sequenced for each individual time point. The approach of sequencing a pool of RNA from biological replicates was also conducted in other studies [41, 45, 46]. We note that using this approach we could not quantify the biological variance. However, the setup comprised two directly opposing culture conditions, each including several harvesting time points (Fig. 1, −N vs +N resupply). Therefore, despite this limitation, we chose this approach to obtain both a structural genome annotation refinement (Additional file 1: Results) and a first approximation of the transcriptome response of *M. neglectum* to alternating phases of N availability.

A total of 796 million 100 nt paired-end reads were obtained, translating into approximately 33 million fragments for each time point, and thus a coverage of >200-fold assuming a 32 mbp transcriptome. For data processing, the Tuxedo protocol was followed [47]. Accordingly, a genome- and reference-guided approach was conducted, based on read mapping by TopHat2 [48] and transcript assembly and quantification by the Cufflinks suite [49]. Both software tools used the genome assembly from [27] and the improved genome annotation obtained in this study by BRAKER1 [50] as additional input (Additional file 1: Results). Of the Cufflinks tools, we used cuffquant and cuffnorm, instead of cuffdiff due to the absence of sequencing replicates. As a result, normalized transcript abundance values, expressed as fragments per kilobase of exon per million fragments mapped (FPKM), were obtained.

Changes in transcript abundances relative to the reference time point N_0 were expressed as log2-transformed fold change (FC) values. We selected an absolute log2-FC > 1 to define a gene as responsive to the treatment, whereas an absolute log2-FC between 0 and 1 classified a gene as not-responsive, as has been done in previous studies [15, 46, 51, 52]. Interestingly, we observed in MA plot from previous studies that most absolute log2-FC values greater than ~1 were also statistically significant [53–56]. However, we stress that FC is not indicative of any statistical significance, and that furthermore less abundant transcripts might require higher absolute FC values to detect statistical significance than more abundant transcripts [56]. The FC threshold used in our study was supported by our own data for the expression of housekeeping genes. For those, the absolute log2-FC of at most one of the eight time points of −N conditions was >1, whereas the remaining FC values of −N conditions were ≤1. Although most housekeeping genes showed a transient transcriptional response in the r+N stage, their expression usually relaxed to the range of absolute log2-FC ≤ 1 after 8 h of N resupply (Additional file 1: Figure S2a).

The data were integrated into a database, which is available at https://tdbmn.cebitec.uni-bielefeld.de. For *M. neglectum*, the structural and functional annotation of the queried transcript locus is displayed, as well as the transcript abundance profile during the three different stages of N availability. In addition, published transcriptome datasets from other microalgal species that were also subjected to −N treatment were integrated into this database, enabling inter-species comparisons of transcript changes to −N conditions (Additional file 1: Results).

### Transcriptome reconstruction and quantification

20,751 transcript loci were assembled by Cufflinks [47], which contained a total of 35,146 isoforms. The higher number of isoforms was predominantly due to the presence and absence of untranslated regions (UTRs). While UTRs were predicted by Cufflinks, they were not included in the reference annotation. Therefore, 85% of all loci had either a single or two isoforms attributed, i.e., the provided “UTR-free CDS isoform” and the fully annotated version including UTRs. For the remaining 15%, it was checked whether evidence for alternative splicing and dominant isoform switching [49] could be detected. This was found for at least four genes (Additional file 1: Results, Figures S3–S6); however, a more detailed analysis is required to further investigate the extent and effect of alternative splicing and dominant isoform switching in *M. neglectum*.

The top 100 most abundant transcripts under exponential growth conditions were annotated as proteins involved in the cellular processes translation (52%) and photosynthesis (28%) (Additional file 2). Two examples are a putative 60 S ribosomal protein L13a and a putative chlorophyll a-b binding protein (XLOC_013860, FPKM at N_0 = 4715 and XLOC_000814, FPKM at N_0 = 10,978, respectively). This has also been noted for *B. sudeticus* [23]. Interestingly, although the majority of transcripts assigned to photosynthesis were strongly decreased in the l−N stage (Additional file 2), a few exhibited both a high abundance and a stable expression pattern under −N conditions. A notable example is one isoform of RuBisCo small subunit (*rbcS2*, XLOC_007679, median FPKM = 10,028). Further examples for transcripts with highest abundances and stable expression patterns under −N conditions are a putative component of the cytosolic large 60 S ribosomal subunit (*rpl7ae*, XLOC_000987, median FPKM = 3838) and a putative elongation factor (putative fragment pair XLOC_005939 and XLOC_012699, median FPKM = 2413 and 2393, respectively) (Additional file 1: Figure S2b). Importantly, these genes are ideal candidates for cloning of the promoter regions to efficiently drive transgene expression in subsequent genetic engineering studies.

**Fig. 3:**
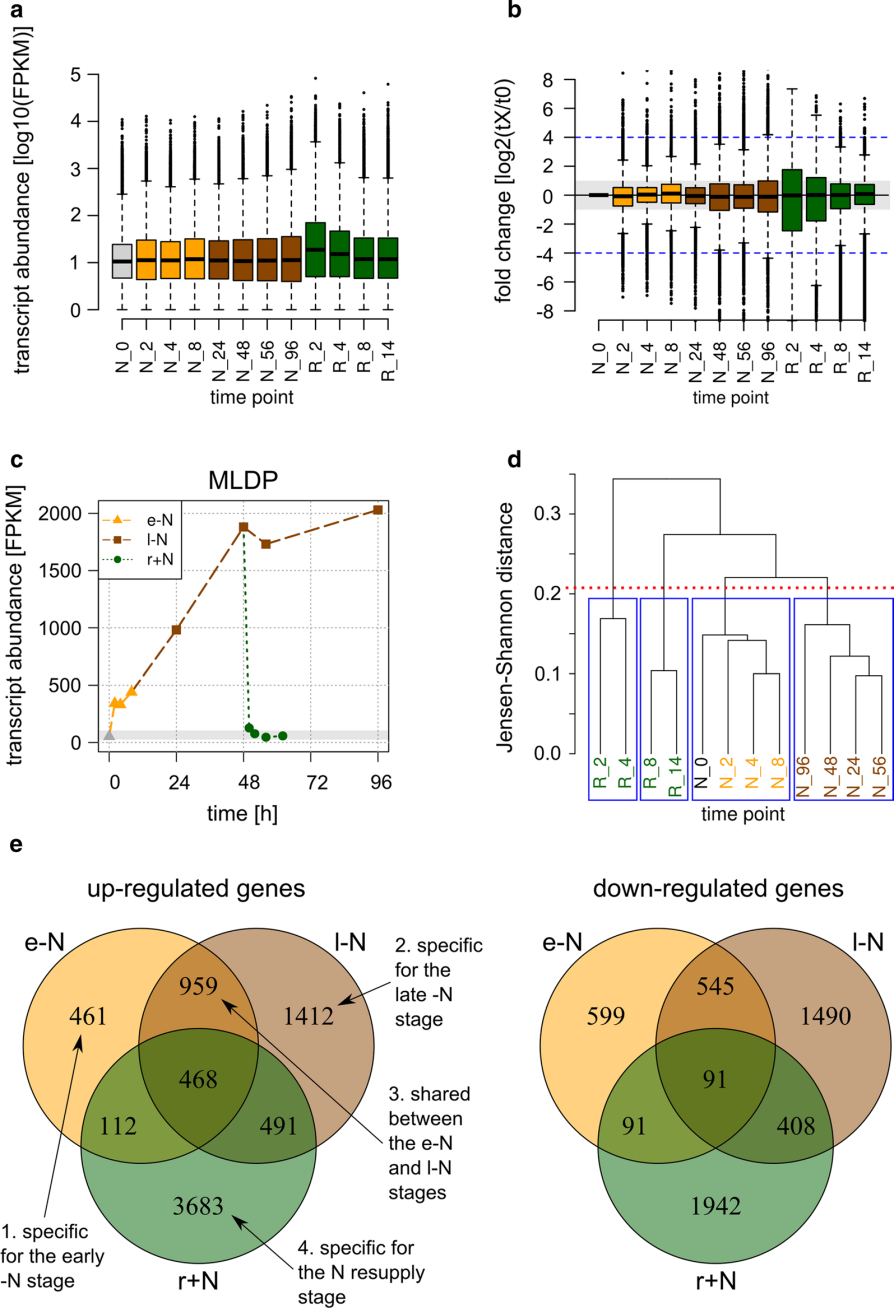
Overview of the transcriptome dataset analysis. **a** Distribution of absolute transcript abundances as FPKM values from all time points on a half-log scale (log10). In the *box-whisker plot* representation, the *thick line* represents the median value, the *colored box* represents the interval between the first and third quartiles, the *two whiskers* indicate the respective 1.5× interquartile ranges, and the *black dots* mark the outliers. **b** Distribution of relative transcript abundance changes normalized to the reference time point N_0, expressed as fold change (FC) on a half-log scale (log2); *box-whisker plots* are as in (**a**). The *black vertical line* highlights the zero line (no regulation), while the *light gray box* indicates the threshold range where absolute log2-FC < 1 indicating no response to −N or N resupply. The *dashed blue line* indicates the threshold above which a transcript was considered as transcriptionally highly regulated, and above which the log2-FC was restricted to 4 and appropriately highlighted by *darker color* in Figs. 4, 5, 6 and Additional file 1: Figure S10. **c** Expression profile of the MLDP gene (XLOC_008097). *Different colors* represent the three stages of nitrogen availability: early −N (e−N, *orange*), late −N (l−N, *brown*), and N resupply (r+N, *green*). The reference time point N_0 is shown in gray. The *light gray box* highlights the threshold range where the absolute log2-FC relative to N_0 was less than one. **d** Dendrogram of a hierarchical clustering of time points using the CummeRbund package [154] with default settings. The *red dotted line* indicates the applied threshold yielding the four clusters (*blue frames*). **e** Venn diagrams of shared genes between the three stages of N availability. The sets on the left consist of genes classified as up-regulated by mean FC during the e−N stage (*orange*), during the l−N stage (*brown*), and during the r+N stage (*green*). On the right, the same was done for down-regulated genes

**Fig. 4:**
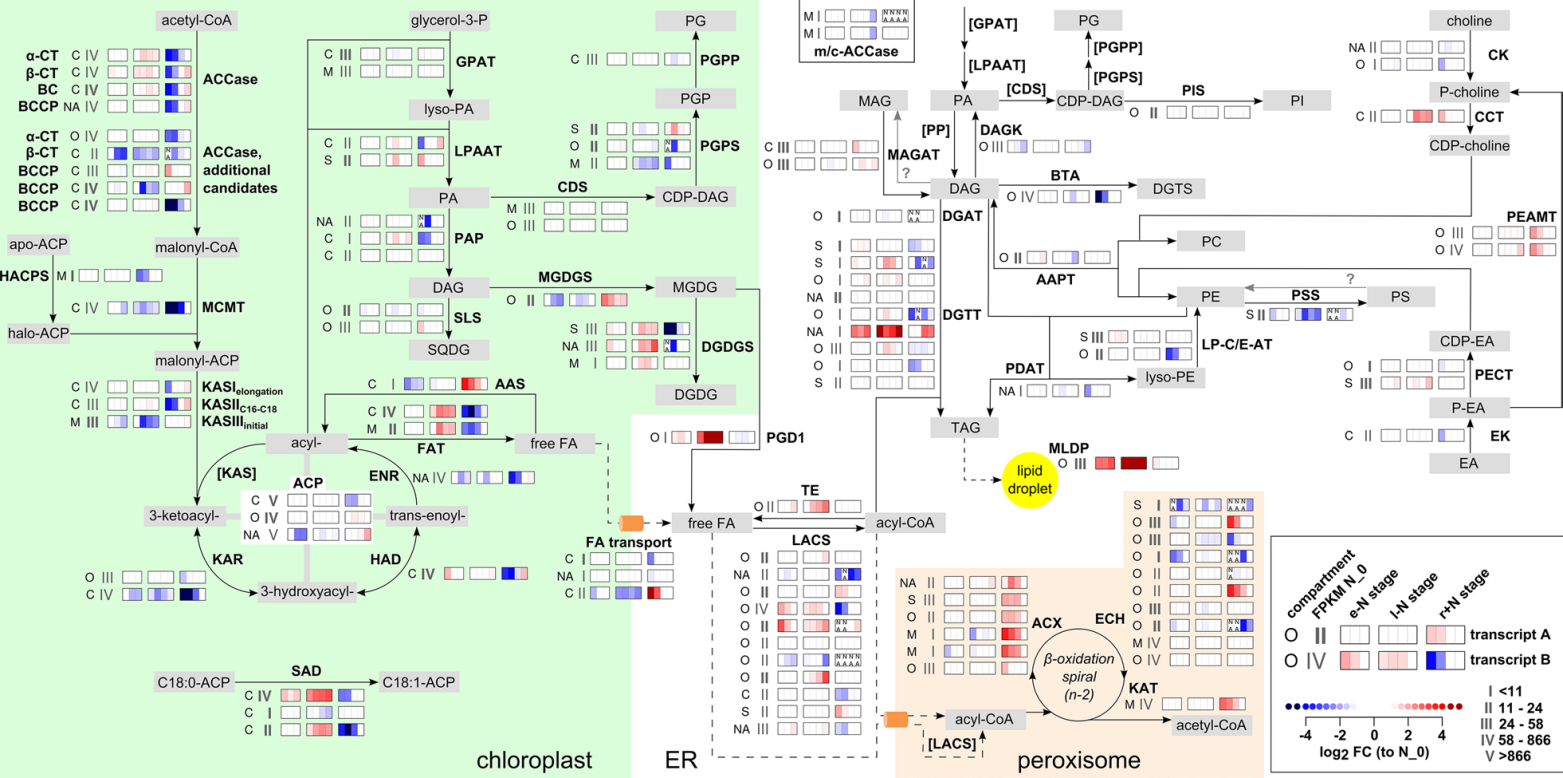
Schematic representation of the putative enzymatic steps of the glycerolipid metabolism in *M. neglectum*, including the transcriptional responses to N starvation (stages e−N and l−N) or N resupply (stage r+N). Enzymatic steps are represented by *solid arrows* and transport processes by *dashed lines*. For simplicity, PDAT is drawn utilizing PE, but has been shown to also use other lipid substrates [69]. Fatty acid desaturation steps are not shown, except for the generation of oleic acid (C18:1). The localization is drawn according to [8] and for additional reactions according to [87]. Each step has at least one transcript associated, and the putative localization is indicated on the *left* (*C* chloroplast, *M* mitochondrion, *O* other, *S* secretory pathway, *NA* localization prediction not possible due to truncation). The section with the *gray Roman numerals* next to the predicted localization shows the binned transcript abundance at the reference time point N_0. Five abundance categories are defined: I = below 50% percentile abundance, II = 50–75% percentile abundance, III = 75–90% percentile abundance, IV = 90–99% percentile abundance, V = >99% percentile abundance; see also legend on the *bottom right* and Fig. 3a for the distribution of FPKM values at N_0. *Bold Roman numerals* indicate that the respective gene is likely not fragmented, whereas normal font style indicates that only the transcript abundance of the putative fragment containing the 5′ end is shown. The transcription profile of each enzyme is represented by *three color boxes*, representing the three different cultivation stages investigated in this work (e−N, l−N, r+N). In each of the *boxes*, the transcriptional regulation at the individual harvesting time points relative to time point zero (N_0) is indicated by *color-coded bars* (*red* up-regulation, *blue* down-regulation compared to N_0). *White bars* are shown if the change in relative transcript abundance was between 50 and 200% (absolute log2-FC < 1). The tag “NA” (not available) is added if the absolute transcript abundance (as FPKM) at that time point was less than 1.0, which was set as the minimum threshold for reliable transcript abundance estimation. The full annotations of the corresponding genes are given in Additional file 4. *ACCase* acetyl-CoA carboxylase, *ACP* acyl carrier protein, *ACX* acyl-CoA oxidase, *AAPT* aminoalcoholphosphotransferase (putatively dual substrate specificity producing PC and PE), *BTA* betaine lipid synthase, *CCT* CTP:phosphorylcholine cytidylyltransferase, *CDS* CDP-DAG synthase, *CK* choline kinase, *DGAT* diacylglycerol acyltransferase type 1, *DGDGS* digalactosyldiacylglycerol synthase, *DGTT* diacylglycerol acyltransferase type 2, *ECH* multifunctional protein containing a 2E-enoyl-CoA hydratase and a 3S-hydroxyacyl-CoA dehydrogenase, *EK* ethanolamine kinase, *ENR* enoyl-ACP reductase, *FAT* acyl-ACP thioesterase, *GPAT* glycerol-3-phosphate acyltransferase, *HAD* hydroxyacyl-ACP dehydrase, *KAR* ketoacyl-ACP reductase, *KAS* ketoacyl-ACP synthase, *KAT* 3-ketoacyl-CoA thiolase, *LACS* long-chain acyl-CoA synthetase, *LPAAT* lysophosphatidic acid acyltransferase, *LP-C/E-AT* lysophosphatidylcholine/ethanolamine acyltransferases, *m/c-ACCase* mitochondrial or cytosolic ACCase, *MCMT* malonyl-CoA:acyl carrier protein malonyltransferase, *MGDGS* monogalactosyldiacylglycerol synthase, *MLDP* major lipid droplet protein, *PAP* phosphatidic acid phosphatase, *PDAT* phospholipid:diacylglycerol acyltransferase, *PEAMT* phosphoethanolamine N-methyltransferase, *PECT* CTP:phosphorylethanolamine cytidylyltransferase, *PGD1* plastid galactoglycerolipid degradation lipase, *PGPP* phosphatidylglycerol phosphate, *PGPS* phosphatidylglycerophosphate synthase, *PIS* phosphatidylinositol synthase, *PSD* phosphatidylserine decarboxylase, *PSS* phosphatidylserine synthase, SAD, Δ^9^ stearoyl-ACP desaturase, *SLS* sulfolipid synthase, *TE* acyl-CoA thioesterase, *CDP* cytidine diphosphate, *CoA* coenzyme A, *DAG* diacylglycerol, *DGDG* digalactosyldiacylglycerol, *DGTS* diacylglycerol-N,N,N-trimethylhomoserine, *EA* ethanolamine, *ER* endoplasmic reticulum, *FA* fatty acid, *LPE* lysophosphatidylethanolamine, *MGDG* monogalactosyldiacylglycerol, *PA* phosphatidic acid, *PC* phosphatidylcholine, *PE* phosphatidylethanolamine, *PGP* phosphatidylglycerolphosphate, *PG* phosphatidylglycerol, *PI* phosphatidylinositol, *SQDG* sulfoquinovosyldiacylglycerol, *TAG* triacylglycerol

**Fig. 5:**
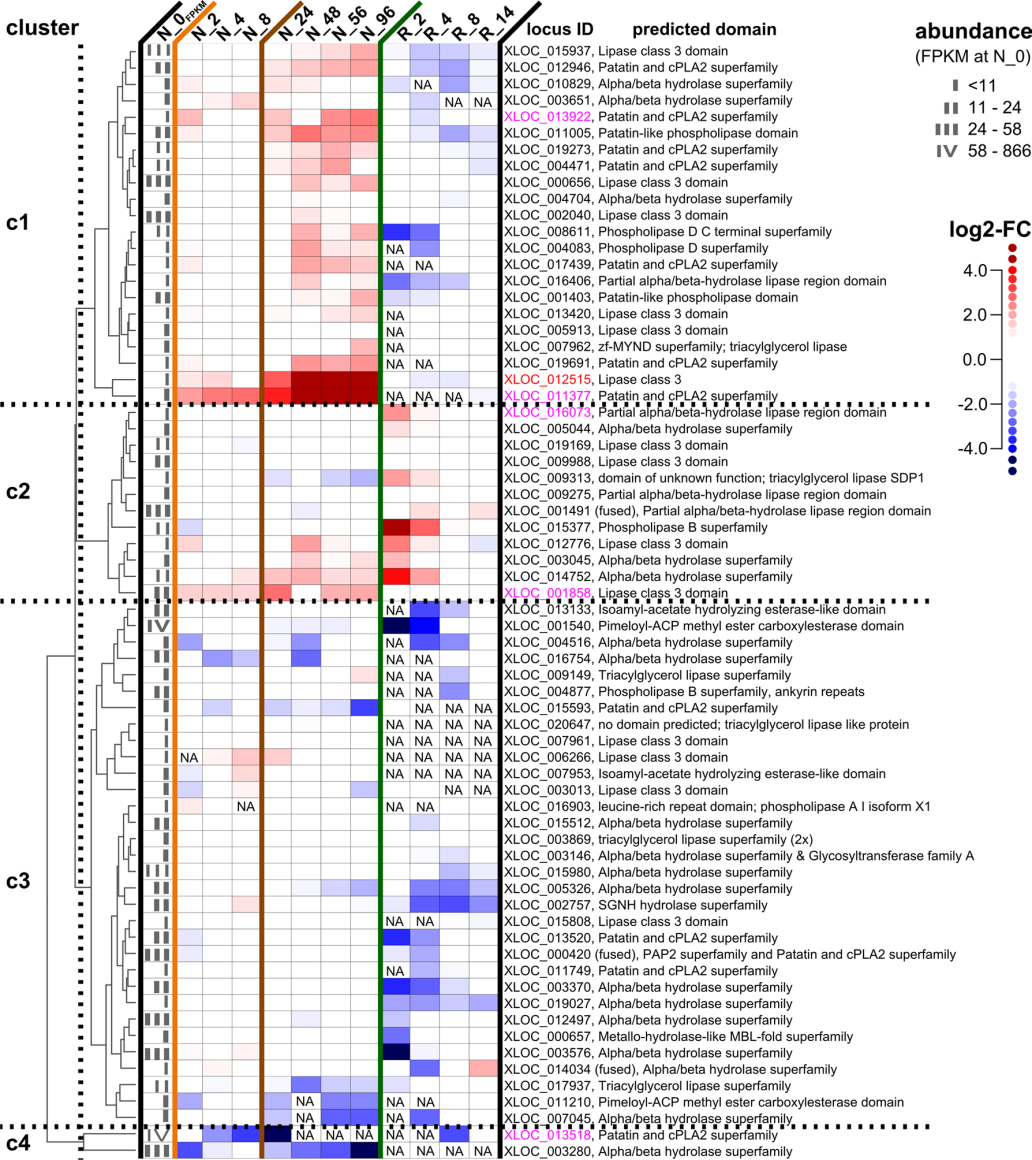
Identification of lipase candidates possibly implicated in lipid accumulation. The transcription profiles of putative lipases of *M. neglectum* were subjected to hierarchical clustering with complete linkage based on log2-FC values with distance metric defined as Euclidean distance. The resulting dendrogram is shown on the *left*. The dendrogram was divided into four clusters as indicated by the *dotted lines*. The *first column* of the heatmap (N_0_FPKM_) indicates the binned transcript abundance value (FPKM) at the reference time point N_0, i.e., the abundance category. The category “I” represents the range of FPKM values of 1 ≤ FPKM ≤ 11 (below median expression), category “II” the range 11 ≤ FPKM ≤ 24 (between 50 and 75% percentile expression), category “III” the range 24 ≤ FPKM ≤ 58 (between 75 and 90% percentile expression), and category “IV” the range 58 ≤ FPKM ≤ 866 (between 90 and 99% percentile expression); see Fig. 3a for the corresponding *box plot*. The remaining columns show the transcriptional regulation at the individual harvesting time points relative to time point zero (N_0), given as *color-coded boxes*. *Red color* represents higher transcript abundance and *blue* lower transcript abundance compared to time point N_0. The tag “NA” (not available) is used when the absolute transcript abundance (as FPKM) at the respective time point was less than 1.0. The three stages of N availability are separated by *vertical lines*, where *orange* represents the e−N stage, *brown* the l−N stage, and *green* the r+N stage, respectively. The locus ID and the predicted domain of each lipase transcript are given on the *right*. As an example for transcriptional regulation of lipase candidates possibly implicated in TAG accumulation rather than TAG degradation, the PGD1 candidate of *M. neglectum* is highlighted in *red color*. Other transcripts mentioned in the text are shown in *magenta*

**Fig. 6:**
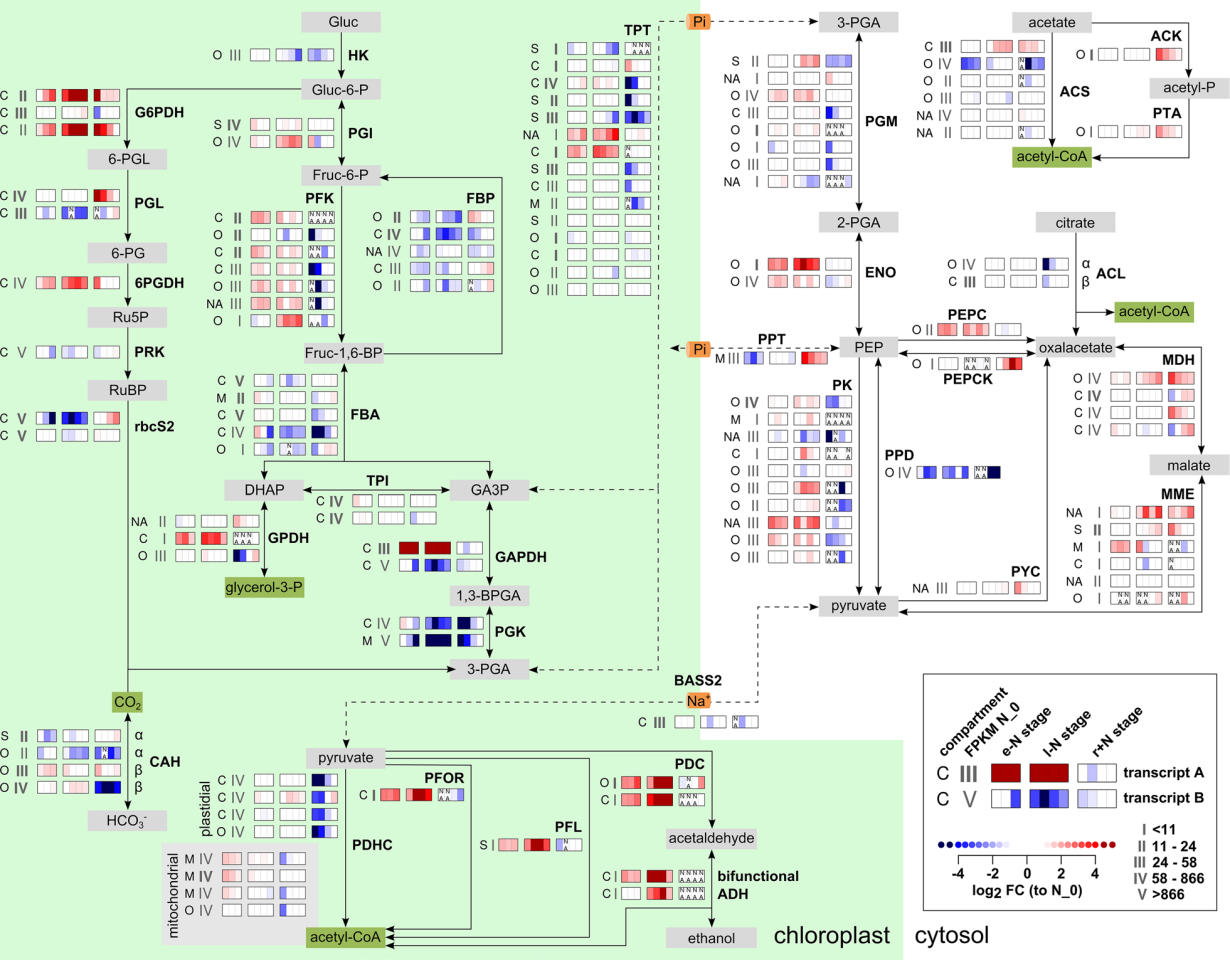
Schematic representation of the putative enzymatic steps of the central carbon metabolism in *M. neglectum*, including the transcriptional responses to N starvation (stages e−N and l−N) or N resupply (stage r+N). Enzymatic steps are represented by *solid arrows* and transport processes by *dashed lines*. The localization is drawn according to [32]. An expression pictogram plot for each enzymatic step is shown. It indicates the putative localization (*C* chloroplast, *M* mitochondrion, *O* other, *S* secretory pathway, *NA* not available due to truncation), transcript abundance at the reference time point N_0 (*gray Roman numerals*), and the transcriptional profile during the three stages of N availability. The three stages are represented by *three color boxes*, and individual time points by *vertical color-coded bars* in those boxes. The tag “NA” (not available) is added if the absolute transcript abundance (as FPKM) at that time point was less than 1.0. See Fig. 4 for a detailed description. The full annotations of the corresponding genes are given in Additional file 4. *1,3-BPA* 1,3-bisphosphoglycerate, *2-PG* 2-phosphoglycerate, *3-PG* 3-phosphoglycerate, *6-PG* 6-phosphogluconate, *6-PGL* 6-phosphogluconolactonase, *6PGDH* 6-phosphogluconate dehydrogenase, *ACL* ATP-citrate-lyase, *ACS*, acetyl-CoA synthetase, *ADH* bifunctional acetaldehyde-alcohol dehydrogenase, *BASS2* sodium/pyruvate cotransporter BASS2 (bile acid-sodium symporter), *CAH* carbonic anhydrase, *DHAP* dihydroxyacetone phosphate, *ENO* enolase, *FBA* fructose-bisphosphate aldolase, *FBP* fructose-bisphosphate phosphatase, *fruc* fructose, *G6PDH* glucose-6-phosphate dehydrogenase, *GA3P* glyceraldehyde-3-phosphate, *GAPDH* glyceraldehyde-3-phosphate dehydrogenase, *gluc* glucose, *GPDH*, glycerol-3-phosphate dehydrogenase, *HK* hexokinase, *MDH* malate dehydrogenase, *MME* malic enzyme, *PDC* pyruvate decarboxylase, *PDHC* pyruvate dehydrogenase complex, *PEP* phosphoenolpyruvate, *PEPC* phosphoenolpyruvate carboxylase, *PEPCK* phosphoenolpyruvate carboxykinase, *PFK* phosphofructokinase, *PFL* pyruvate-formate-lyase, *PFOR* pyruvate-ferredoxin-oxidoreductase, *PGI* glucose-6-phosphate isomerase, *PGK* phosphoglycerate kinase, *PGL* 6-phosphogluconolactonase, *PGM* phosphoglycerate mutase, *PK* pyruvate kinase, *PPT* phosphoenolpyruvate transporter, *PRK* phosphoribulokinase, *PYC* pyruvate carboxylase, *Ru5P* ribulose-5-phosphate, *RuBP* ribulose-1,5-bisphosphate, *TPI*, triose phosphate isomerase, *TPT* triose phosphate transporter

The range of FPKM values covered more than four orders of magnitude, and this range distribution was approximately similar in the different stages of N availability (Fig. 3a). The log2-FC values (relative to N_0) of most transcripts were between −1 and 1 at most time points (gray box in Fig. 3b). As noted above, this interval was accordingly defined as the “not-responsive range” (Additional file 1: Figures S2 and S10a). No alteration of the expression of the majority of genes by the experimental conditions is in accordance with the null hypothesis for the determination of significant differentially expressed genes [55]. The upper values of the 1.5-fold interquartile ranges of log2-FC values were for most time points less than four (dotted blue line in Fig. 3b). Therefore, transcripts with log2-FC values of more than four were summarized as highly regulated, and this was visually highlighted by a darker color (see Fig. 3c for the extent of up-regulation of MLDP in the l−N stage, which is indicated in Fig. 4 by a dark red color for the MLDP transcript at the time points from the l−N stage). In contrast, the range between absolute log2-FC values larger than one and less than four was linearly color-coded (see figure keys in Figs. 4, 5, 6).

### Effect of N resupply on gene expression of selected examples

Nitrogen resupply treatment was applied to reverse the phenotypic effects of nutrient starvation and the efficiency of the selected procedure can be exemplified by the profile of the transcript encoding for the major lipid droplet protein (MLDP, Fig. 3c). TAGs, which are accumulated in response to −N treatment, are stored as lipid droplets inside the cell [28, 57], of which MLDP is a major structural protein [30, 58]. Therefore, as expected, a strong increase of the MLDP transcript levels was detected during the two stages of N starvation (e−N and l−N), while relaxation of gene expression occurred immediately upon N resupply in the r+N stage (Fig. 3c).

The N resupply treatment facilitated the assignment of potential functions to genes whose gene products putatively catalyze similar enzymatic reactions. For instance, two transcripts were annotated as ferredoxin-NADP^+^-reductase, a central enzyme of photosynthesis. While the first transcript was strongly up-regulated in the l−N stage (putative fragment pair XLOC_015550 and XLOC_016383), the second transcript was strongly down-regulated in this stage (XLOC_001499) (Additional file 1: Figure S2c). Interestingly, this pattern was reversed when nitrogen was resupplied and expression reverted to pre-starvation levels of the reference time point (Additional file 1: Figure S2c). This indicates that during nutrient starvation the former enzyme might be involved in maintaining photosynthetic electron flow at reduced availability of NADP^+^ levels or in photoprotective release of excitation pressure when demand for NADPH is low, for instance by redistribution of electrons to various redox reactions, which has been described for one of two leaf ferredoxin-NADP^+^-reductase genes of *A. thaliana* [59]. The latter ferredoxin-NADP^+^-reductase of *M. neglectum* could be important under environmental conditions supporting fast cell growth when demand for photosynthetically provided NADPH is high. The opposing expression patterns might also be indicative of a modulation of photosynthetic electron flow towards increased cyclic electron flow under −N conditions. Cyclic electron flow generates ATP [60] and was found to be important for neutral lipid accumulation and to be increased under both autotrophic and mixotrophic −N conditions in *C. reinhardtii* [61]. A second example is the transcriptional regulation of two isoamylase genes. Both genes were expressed under −N conditions in a stable pattern, except for the first time point of −N conditions, at which a gentle transcriptional regulation (~threefold) in opposite directions was observed (Additional file 1: Figure S2d). In the r+N stage however, both genes were contrastingly up- or down-regulated at three of four time points, respectively (Additional file 1: Figure S2d). As the r+N stage was characteristic of storage compound degradation, it is tempting to speculate that the up-regulated isoamylase candidate is implicated in starch degradation (XLOC_001619), whereas the down-regulated one is implicated in starch synthesis (putative fragments XLOC_004804 and XLOC_012040).

It should be mentioned that the N resupply approach furthermore allowed better interpretation of some of the –N transcriptome data. As an example, the transcript abundances of the subunits of the plastidial pyruvate dehydrogenase complex (cpPDHC) were approximately constant in both the e−N and l−N stages (Additional file 1: Figure S2e). However, in the r+N stage, a transient strong down-regulation was observed (Additional file 1: Figure S2e). This shows that cpPDHC was subjected to transcriptional regulation, which in our setup would not have been detected without the r+N treatment. Therefore, the differential transcriptional response of cpPDHC under −N and N resupply conditions indicates that under −N conditions cpPDHC expression is actively maintained, which is reasonable, because cpPDHC converts pyruvate to acetyl-CoA (CoA, coenzyme A), the carbon precursor for FA synthesis (see “Discussion”).

### Global transcriptional responses in the three different stages of N availability

The differentiation into three distinct stages of N availability was the backbone for the interpretation of transcriptional patterns in this study. However, this differentiation was initially solely based on metabolic data, i.e., on the timing of starch and TAG accumulation, as well as of TAG degradation (Fig. 2b, c; Additional file 1: Figure S1). We therefore investigated whether the transcriptomes from the twelve time points would accordingly cluster into the three stages. Towards this end, we applied hierarchical clustering based on Jensen–Shannon distance [49, 62]. From visual evaluation of the resulting dendrogram, four clusters could be distinguished (Fig. 3d). The first cluster consisted of the transcriptomes from the time points of the e−N stage, whereas the second cluster consisted of those from the l−N stage (Fig. 3d). The third and the fourth clusters each contained two of the four transcriptomes from the time points of the r+N stage (Fig. 3d). The reason for the unexpected division of the r+N data into two separate clusters was likely of technical nature, most likely due to the higher magnitude of transcript abundance values at the R_2 and R_4 time points compared to R_8 and R_14 (larger box size and whiskers in Fig. 3a), because the distance metric relies on the extent of change in relative expression [49]. Therefore, the three different stages of nitrogen availability also manifested on the level of the transcriptome data sets.

In order to dissect the transcriptional responses into those being shared between the e−N and l−N stages, and those restricted to either the e−N or l−N stage, as well as those unique to the r+N stage, an analysis of shared responsive genes was performed (Fig. 3e; Additional file 1: Figure S7). As expected, more responsive genes were shared between the e−N and l−N stages, compared to the r+N stage, which was true for both up- and down-regulated genes (Fig. 3e). To subsequently identify the cellular processes in which the respective genes were involved, a gene ontology (GO) term enrichment analysis was performed (Additional file 1: Table ST1). GO terms represent unified vocabulary to annotate genes and gene products [63]. GO terms are organized in a hierarchical structure, with broader vocabulary at the higher level (e.g., “*signal transduction*”) and more specific vocabulary at the lower levels (e.g., “*cAMP biosynthesis*”) [63]. Three different categories (“roots”) of GO terms are defined, which are biological process, molecular function, and cellular component. An example for the category biological process is the GO term “*translation*,” which is a child of the GO term “*gene expression*,” and a parent to the GO terms “*translational initiation*,” “*translation elongation*,” and “*translation termination*” [63]. We restricted our analysis to the category biological process, because we were interested in the cellular processes that were subjected to transcriptional regulation in the three stages.

As a result, the enriched GO terms of the genes whose up-regulation was restricted to the e−N stage were indicative of an induction of cell division (Additional file 1: Table ST1). Furthermore, the GO term “*microtubule*-*based processes*” was enriched (Additional file 1: Table ST1). GO terms enriched among genes down-regulated specifically in the e−N stage included “*chlorophyll biosynthesis process*,” “*aromatic amino acid family biosynthetic process*,” and “*translation*” (Additional file 1: Table ST1).

Enriched GO terms of genes whose up-regulation was restricted to the l−N stage were “*protein phosphorylation*,” “*fatty acid biosynthetic process*,” “*chlorophyll catabolic process*,” and “*lipid catabolism process*” (Additional file 1: Table ST1). As the l−N stage was characterized by TAG and total lipid hyperaccumulation and thus central to this study, the top 100 genes showing the highest degree of up-regulation in this stage were additionally determined (Additional file 2). This was to identify the processes that were subjected to the highest transcriptional induction. These genes encoded almost the complete set of N assimilation proteins (Additional file 1: Results), including genes annotated as urea carboxylase. This indicates that *M. neglectum* is able to use urea as an external nitrogen source, which was also confirmed phenotypically in a separate experiment (Additional file 1: Figure S8). Interestingly, an MYB-domain containing transcription factor (XLOC_013389) was also found in this set, possibly implicated in core metabolic regulation (Additional file 1: Results). GO terms enriched among genes down-regulated specifically in the l−N stage included “*photosystem II assembly*” and “*proline cis*–*trans isomerization*” (Additional file 1: Table ST1).

The GO term enrichment profile of genes up-regulated in both the e−N and the l−N stages suggested an induction of the tricarboxylic acid cycle, glycolysis, and arginine biosynthesis (Additional file 1: Table ST1). Interestingly, the GO term “*ATP hydrolysis coupled proton transport*” was also enriched (Additional file 1: Table ST1), which was assigned to eleven genes that encoded putative vacuolar-type (V-type) ATPases. The GO term enrichment profile of genes down-regulated in both stages consisted mostly of photosynthesis-associated processes, such as light-harvesting and the non-oxidative pentose-phosphate shunt (Additional file 1: Table ST1). Interestingly, the GO term “*cysteine biosynthetic process*” was also found to be enriched in this set.

The resupplementation with N restored the unstressed cellular state allowing for exponential growth (Additional file 1: Figure S1). GO terms enriched among genes up-regulated specifically in the r+N stage were indicative of the respective processes, such as “*ribosome biogenesis*” and “*photosynthesis*” (Additional file 1: Table ST1). Enriched GO terms of genes whose down-regulation was restricted to the r+N stage included “*tricarboxylic acid cycle*,” “*fructose 6*-*phosphate metabolic process*,” and “*ATP hydrolysis coupled proton transport*” (Additional file 1: Table ST1). Since they were previously enriched in the set of genes up-regulated in both the e−N and l−N stages, it is tempting to speculate that these cellular processes are central to cope with −N conditions.

### Reconstruction of pathway maps and the glycerolipid metabolism of *M. neglectum*

For reconstruction of metabolic pathway maps, tBLASTx comparison of known enzymes from *C. reinhardtii* and *A. thaliana* was performed with the transcriptome of *M. neglectum*. After tBLASTx application, redundancy in the list of candidate genes was minimized by the identification of fragmented genes, which were the result of the assembly status of the genome of *M. neglectum* as 6739 scaffolds [27]. For instance, a protein AB might be encoded by two individual genes on two different scaffolds, such that part A is encoded as a first individual gene at the end of a first scaffold, while part B is encoded as a second individual gene at the beginning of a second scaffold. Importantly, however, as both genes are fragments of the same protein AB, their transcriptional profiles are identical. Approximately half of all gene models were located at a scaffold margin, and accordingly tagged as putatively truncated. For these genes, the transcript data acquired in this study allowed the assignment of gene fragments to fragment pairs. As an example, two candidate genes for the α-carboxyltransferase subunit of the acetyl-CoA carboxylase (ACCase) complex, a central enzyme of FA synthesis, were tagged as likely fragmented and had very similar expression patterns (XLOC_015237 and XLOC_12365, Additional file 1: Figure S9). In addition, their domain structures matched, because both had a predicted crotonase-like superfamily domain, appropriately truncated at the C- and N-terminus, respectively (Additional file 1: Figure S9). Accordingly, we considered these two sequences as a fragment pair. To minimize redundancy, only the fragment containing the N-terminus of the respective protein was retained for further analysis (XLOC_015237), because it optionally encodes the targeting peptide, hence allowing for localization prediction [32]. An obvious consequence of gene fragmentation was an over-estimation of the gene content of *M. neglectum* for a specific enzymatic step. For instance, two transcript loci were identified by tBLASTx search for phosphoribulokinase, which, however, were identified as a fragment pair, due to almost identical expression patterns (Additional file 3, XLOC_002711 and XLOC_006065, respectively) and matching domain structures (C- and N-terminal truncated Udk superfamily hit, respectively) [64]. Accordingly, *M. neglectum* most likely contains only one phosphoribulokinase enzyme, similar to *C. reinhardtii* [65], *N. oceanica* [66], and *P. tricornutum* [67]. A further consequence was over-estimation of the FPKM values of the individual parts of a fragment pair, because transcript length is taken into account for FPKM calculation [47]. This, however, is a systematic bias and cancels out during FC calculation, as long as transcript length remains unchanged during the time course experiment. Therefore, the over-estimation of FPKM values of fragment pairs did not affect the evaluation of relative patterns (intra-gene comparisons), which was the central element for subsequent pathway analysis.

Following this approach, the glycerolipid metabolism of *M. neglectum* was reconstructed (see “Discussion”). Briefly, FA synthesis in the chloroplast generates acyl chains, which are esterified to glycerol-3-phosphate in the Kennedy pathway to yield various membrane lipids or TAGs (Fig. 4). The direct precursor for most glycerolipids, including TAG, is diacylglycerol (DAG). DAG accordingly represents a central intermediate of the glycerolipid metabolism [8]. The acylation of DAG yields TAG, and an important group of enzymes catalyzing this reaction are the diacylglycerol acyltransferases, which use DAG and acyl-CoA as substrates [8]. Diacylglycerol acyltransferase enzymes are divided into several classes, and the first two are responsible for the bulk of TAG synthesis in plants [68], abbreviated as DGAT and DGTT in *C. reinhardtii*, respectively [10]. This route of TAG formation is also referred to as the acyl-CoA-dependent route. The alternative, the acyl-CoA independent route, refers to the transacylation of DAG and an acyl donor glycerolipid molecule (phospholipid, galactolipid or DAG [69]). This reaction produces a TAG and a lyso-lipid molecule, and is catalyzed by phospholipid:diacylglycerol acyltransferase (PDAT) [8].

### Transcriptional regulation of the glycerolipid metabolism of *M. neglectum* during the two stages of nitrogen starvation and the stage of nitrogen resupply

We developed a modified heat map visualization (Additional file 1: Results), which contains both the extent of differential transcriptional regulation at the individual time points of the three stages of N availability as well as the absolute transcript abundance at the reference time point (N_0), represented by one of five abundance categories (category I–V). As additional information, the putative protein localization as predicted by PredAlgo software [70] is indicated (Fig. 4). Using this modified heat map visualization, the transcriptional regulation of the glycerolipid metabolism of *M. neglectum* during the three stages of N availability was analyzed. In the e−N stage, 3- to 8-fold increased transcript abundances were observed for MLDP, one stearoyl-ACP (ACP, acyl carrier protein) desaturase (SAD) candidate and two long-chain acyl-CoA synthetase (LACS) candidates (Fig. 4, MLDP, SAD, and LACS, respectively). Genes down-regulated to a high extent (≥eightfold) were not found in the e−N stage, except for a hypothetical subunit of the ACCase complex (Fig. 4, additional β-CT). However, its transcript abundance under logarithmic growth conditions (N_0) was only 20% of a second candidate (Additional file 3, XLOC_016656 and XLOC_015237, respectively). Therefore, the overall contribution of the second gene’s product to the flux of FA synthesis might be negligible. Seven genes were subjected to more subtle decreases in transcript abundances in the e−N stage; those were implicated in FA synthesis, thylakoid membrane assembly, and FA degradation (Fig. 4, KASIII, KAR, ENR, ACP, and MGDGS as well as ECH, respectively). Regarding the latter, however, down-regulation of the respective gene was also observed in the r+N stage (Fig. 4, ECH), rendering interpretation difficult.

In the l−N stage, the highest increase in transcript abundance was noted for MLDP, correlating with the increasing number and diameter of lipid droplets under −N conditions [28]. Additionally strongly up-regulated (10- to >16-fold) were acyl-ACP thioesterase (FAT), SAD, and the putative lipase PGD1 (Fig. 4). More gentle up-regulation was noted for CTP:phosphocholine cytidylyltransferase (~fourfold) putatively involved in phosphatidylcholine (PtdCho) synthesis, as well as for three of four central subunits of the ACCase complex (~twofold) (Fig. 4, CCT and ACCase, respectively). Contrasting the transcriptional induction of PtdCho synthesis, reduced transcript abundances were noticed for the genes implicated in the synthesis of phosphatidylserine and phosphatidylglycerol in the l−N stage (Fig. 4, PSS and PGPS, respectively). Thylakoid membrane lipid synthesis was also differentially regulated, with monogalactosyldiacylglycerol (MGDG) synthesis being transcriptionally repressed and digalactosyldiacylglycerol (DGDG) synthesis induced (Fig. 4, MGDGS and DGDGS, respectively). This might be indicative of an alteration of the thylakoid membrane architecture, because MGDG has a conical shape, does not form bilayers, and its accumulation results in a negative membrane curvature, while DGDG has a cylindrical shape and forms bilayers [71]. Three steps of FA synthesis were down-regulated in the l−N stage (Fig. 4, MCMT, KASIII, and KAR). The remaining down-regulated genes had 5–40% lower transcript abundance values than other genes associated with the same function at the reference time point N_0 (Fig. 4, additional β-CT, BCCP, and SAD); therefore, the effect of their down-regulation in the l−N stage was considered to be negligible.

In the r+N stage, the synthesis of MGDG was transcriptionally induced, while DGDG and diacylglycerol-N,N,N-trimethylhomoserine (DGTS) synthesis was transcriptionally repressed (Fig. 4, MGDGS, DGDGS, and BTA, respectively). FA degradation was transiently sharply up-regulated (Fig. 4, ACX, ECH, and KAT), which is in accordance with the decreasing total lipid content upon N resupply (Additional file 1: Figure S1). Contrastingly, FA synthesis was transcriptionally repressed at almost all individual enzymatic steps during the first 4 to 8 h of N resupply (Fig. 4, ACCase, MCMT, KASII, KAR, HAD, ENR, SAD, FAT, and ACP). Two putative LACS transcripts were transiently induced in the r+N stage, and two of three phosphatidic acid phosphatase candidate genes were transiently down-regulated (Fig. 4, LACS and PAP, respectively).

### Transcriptional regulation of the committed step of TAG synthesis

The only committed reaction to TAG synthesis is the addition of a third acyl chain to DAG [8]. In respect of the acyl-CoA-dependent pathway, four of nine DGTT candidate genes showed a transcriptional induction under −N conditions, while approximately constant abundances were noted for the single putative DGAT transcript (Fig. 4). In the r+N stage, a transient down-regulation was observed for three of the aforementioned DGTT transcripts, as well as for two additional putative DGTT transcripts, and for the DGAT transcript (Fig. 4). In respect of the acyl-CoA independent pathway, the transcript level of the PDAT candidate gene was approximately constant in the three stages of N availability, except for a transient down-regulation in the r+N stage after 2 h of N resupply (Fig. 4). The transacylation reaction catalyzed by PDAT generates a lyso-lipid. Two enzymes putatively catalyzing the re-acylation of this lyso-lipid were identified in *M. neglectum*. The first candidate was transcriptionally induced (~twofold) in the e+N stage, whereas the second candidate was transiently repressed in the r+N stage (Fig. 4, LP-C/E-AT).

### Clustering of transcripts annotated as lipases reveals candidates likely involved in TAG accumulation and TAG degradation

Lipases hydrolyze the ester bond between the glycerol backbone and the acyl chain of TAG and other glycerolipid molecules, yielding a free FA and the corresponding lyso-lipid [8]. The released acyl chain can be subsequently incorporated into other glycerolipids after activation by CoA. By this process, acyl chains can be shuttled between the membrane lipid and TAG pool (another route is transacylation). Acyl chain recycling apparently contributed significantly to early TAG accumulation in *M. neglectum*, because the polar lipid content decreased during the first 2 days of −N conditions (Fig. 2c; see above).

We reasoned that lipases involved in the process of de novo TAG accumulation can be identified according to a transcriptional induction under −N conditions. In contrast, opposing TAG lipases that degrade storage lipids are expected to be down-regulated under −N conditions, but up-regulated upon N resupply. Accordingly in this work, the transcriptional pattern of a lipase candidate gene was used as an indicator for its putative function in storage lipid metabolism.

In *C. reinhardtii*, a correlation between transcriptional regulation and metabolic function was observed for the lipases CrLIP1 and PGD1 (plastid galactoglycerolipid degradation 1) [72, 73]. Whereas CrLIP1 was down-regulated under −N conditions and indirectly implicated in TAG turnover [72], PGD1 was up-regulated under −N conditions and implicated in de novo TAG accumulation [73]. For both genes, a putative homologue was identified in the transcriptome of *M. neglectum*. As expected, the CrLIP1 transcript exhibited a transient sharp increase in the r+N stage (~sixfold at R_2, Fig. 5, XLOC_016073), in agreement with a putative role in storage lipid degradation. Likewise, the PGD1 transcript in *M. neglectum* exhibited a strong induction in the l−N stage (>16-fold, Fig. 5, XLOC_012515), corroborating a putative role in de novo TAG accumulation. Although the expression patterns are clear indicators that these proteins indeed represent homologous enzymes of both microalgal species, biochemical characterization would be required to prove this model and to further determine substrate specificity in *M. neglectum*.

Additional putative lipases were identified according to the gene annotation or their GO term description. This revealed a total of 68 putative lipase transcripts in *M. neglectum*. In order to group these transcripts based on their transcriptional profiles, hierarchical clustering was performed [74], and the resulting dendrogram divided into four clusters, supported by values for the average silhouette width as cluster quality parameter [75, 76] (data not shown).

Most putative lipase genes of the first cluster were characterized by a constant or slightly up-regulated gene expression in the e−N stage, were most strongly up-regulated in the l−N stage, and induction relaxed when nitrogen was supplemented in the r+N stage (Fig. 5, c1). This profile is similar to the PGD1 gene expression pattern which was also part of this cluster (Fig. 5, c1, XLOC_012515), therefore indicating a putative involvement in the process of de novo TAG accumulation under −N conditions. The second cluster contained several lipase candidates which were strongly up-regulated in the r+N stage, including the aforementioned CrLIP1 transcript of *M. neglectum* (Fig. 5, c2, XLOC_016073). Since up-regulation was concomitant with the induction of putative FA degradation genes (beta-oxidation, Fig. 4), it seems likely that these lipase candidates are involved in the process of TAG degradation. The third cluster contained transcripts with approximately stable expression in the e−N and l−N stages, but decreased expression in the r+N stage (Fig. 5, c3). This down-regulation might indicate that the corresponding enzymes are not required for the process of thylakoid membrane reassembly occurring in the r+N stage (Additional file 1: Table ST1); alternatively, they might impair the process of thylakoid membrane reassembly, therefore necessitating down-regulation in the r+N stage. The fourth cluster consisted of only two genes, which were strongly down-regulated in the e−N and l−N stages (Fig. 5, c4). This suggests that the corresponding enzymes might act as suppressors of TAG accumulation under exponential growth conditions.

### Reconstruction and prediction of compartmentalization of the central carbon metabolism of *M. neglectum*

Central to the development of metabolic engineering strategies for improved TAG accumulation is not only the glycerolipid metabolism, but also the central carbon metabolism, as it determines the availability of acetyl-CoA for FA synthesis. This has been demonstrated for *C. reinhardtii*, for which carbon precursor supply was reported to be a key metabolic factor controlling oil biosynthesis under mixotrophic −N conditions [77]. Therefore, we next reconstructed the central carbon metabolism of *M. neglectum* (Additional file 1: Results). Supported by localization prediction, we propose a compartmentalization similar to *C. reinhardtii* [32]. Accordingly, the oxidative pentose-phosphate pathway (OPPP) is entirely plastidial, whereas glycolysis is highly compartmentalized, such that the initial steps of glycolysis take place in the chloroplast, while the later steps from 3-phosphoglycerate to pyruvate are located in the cytosol [32] (Fig. 6). However, *M. neglectum* might be able to perform the initial steps of glycolysis from glucose to the triose phosphates additionally in the cytosol, because *M. neglectum* can utilize glucose as a sole carbon source (Additional file 1: Figure S11), whereas *C. reinhardtii* cannot [78]; this model, however, requires further localization studies.

### Differentially regulated genes of the central carbon metabolism during the three stages of N availability

Most genes of the central carbon metabolism that were responsive in the e−N stage exhibited the same direction of transcriptional regulation also in the l−N stage (see below). Transcriptional induction that was restricted to the e−N stage was observed for the putative mitochondrial pyruvate dehydrogenase complex (Fig. 6, PDHC). A dependence of starch and lipid accumulation on mitochondrial respiration was shown for *C. reinhardtii*, because mitochondrial mutants exhibit impaired starch and lipid accumulation under −N and −S conditions, respectively [79, 80]. Down-regulation of genes specifically in the e−N stage was observed for a putative phosphoenolpyruvate (PEP) transporter, and for one of six acetyl-CoA synthetase candidates (Fig. 6, PPT and ACS, respectively). Up-regulation restricted to the l−N stage was observed for one of two glucose-6-phosphate isomerase candidates, for one of four malate dehydrogenase candidates, and for a second of six acetyl-CoA synthetase candidates (Fig. 6, PGI, MDH, and ACS, respectively). Transcripts with exclusively decreased abundances in the l−N stage were not found. For the two acetyl-CoA synthetase candidates with differential expression under −N conditions, enzymatic activity should be confirmed in future studies. This is because *M. neglectum* can use acetate only to a limited extent for growth (Additional file 1: Figure S11). However, a likely functional glyoxylate cycle is encoded, since both key enzymes, malate synthase and isocitrate lyase, had transcript support and responded to the N resupply treatment by a transient up-regulation (Additional file 3, XLOC_013435 and XLOC_002446, respectively).

Of the transcriptional responses shared between the e−N and the l−N stages, the most pronounced up-regulation (>16-fold) was observed for the OPPP (Fig. 6, G6PDH and 6PGDH). A similarly strong up-regulation was noted for fermentative reactions, which have the production of acetyl-CoA from pyruvate in common (Fig. 6, PFL, PFOR, PDC, and bifunctional ADH). Furthermore, the conversion of 2-phosphoglycerate to PEP, catalyzed by enolase, was transcriptionally strongly induced (4- to 16-fold) (Fig. 6, ENO). 4- to 8-fold up-regulation was detected for one of three glycerol-3-phosphate dehydrogenase candidate genes (Fig. 6, GPDH); the respective enzyme generates glycerol-3-phosphate, which is a substrate for the Kennedy pathway and the backbone of TAG. A less pronounced induction (2- to 5-fold) was noted for PEP carboxylase (Fig. 6, PEPC). This enzyme is characteristic for C4 plants and catalyzes the fixation of CO_2_ by the generation of oxaloacetate from PEP [81].

For glycolysis and gluconeogenesis, an opposing transcriptional regulation was observed under −N conditions. While two committed steps of glycolysis were both up-regulated, one committed step of gluconeogenesis was down-regulated (Fig. 6, PFK, PK, and FBP, respectively). Another transcript implicated in gluconeogenesis, a putative PEP carboxykinase, was also decreased in abundance in the l−N stage, although absolute transcript abundance values were already low at the reference time point N_0 (Fig. 6, PEPCK).

Interestingly, opposite transcriptional patterns within the same enzymatic step under −N conditions were also observed. This applied to both glyceraldehyde-3-phosphate dehydrogenase candidates, as well as to all four carbonic anhydrase candidates (Fig. 6, GAPDH and CAH, respectively). Each putative carbonic anhydrase was specifically responsive in either the e−N or the l−N stage (Fig. 6, CAH), suggesting a tight transcriptional regulation of the carbon concentration mechanism under −N conditions in *M. neglectum*.

Upon N resupply in the r+N stage, only a few enzymatic steps of the central carbon metabolism were transcriptionally induced, while most others were repressed. Interestingly, the aforementioned transcriptional induction of the OPPP under −N conditions was maintained during the first 4 h of N resupply (Fig. 6, G6PDH and PGL). Furthermore, one putative PEP transporter, one of two putative small subunits of RuBisCo, two of four putative malate dehydrogenase enzymes, as well as two of six putative malic enzyme proteins were transcriptionally up-regulated in the r+N stage (Fig. 6, PPT, rbcS2, MDH, and MME, respectively). The transcriptional repression of both putative phosphoglycerate kinase enzymes that had been noticed for the −N phase was continued for the first 4 h in the r+N stage, after which transcript levels normalized to pre-starvation levels (Fig. 6, PGK). A transient sharp down-regulation in the r+N stage was observed for cpPDHC, as well as for four of 15 putative triose phosphate transporters (Fig. 6, PDHC and TPT, respectively). Glycolysis was down-regulated upon N resupply at several enzymatic steps, contrasting the transcriptional induction under −N conditions (Fig. 6, HK, PFK, FBA, PGM, and PK).

### Transcriptional regulation of starch metabolism

Finally, the starch metabolism of *M. neglectum* was reconstructed (Additional file 1: Results). Starch is the major carbon storage molecule in green microalgae and plants, and can amount up to 50% of the dry biomass in *C. reinhardtii* under −N conditions [82]. In *M. neglectum*, the cellular starch content after 1 day of −N conditions amounted to ~35 pg cell^−1^, which was similar to the cellular neutral lipid content at day 8 with ~33 pg cell^−1^ (Fig. 2b, c). We analyzed the transcriptional regulation of both pathways in order to understand the interplay of both storage compound production processes. In summary, most genes assigned to the starch metabolism were subjected to transcriptional regulation (Additional file 1: Figure S10b). However, the changes in transcript abundances did not yield a completely conclusive picture. For instance, genes indicative of starch synthesis and starch degradation were consistently up-regulated during the −N treatment (Additional file 1: Figure S10b), yet cellular net starch levels decreased slightly (Fig. 2b). This discrepancy might be attributed to currently unknown post-transcriptional or post-translational regulation steps of the corresponding catabolic enzymes. Interestingly, the most strongly induced transcripts in the l−N stage were annotated as putative starch phosphorylases, and these transcripts were transiently sharply repressed in the r+N stage (Additional file 1: Figure S10b). This indicates that starch phosphorylase might play a key role in starch metabolism in *M. neglectum*.

## Discussion

In this study, the transcriptional changes in different pathways in the context of microalgal lipid accumulation were investigated in *M. neglectum* under three different stages of N availability. The first two stages represented cellular acclimation processes from nitrogen replete to nitrogen-free conditions. Removal of nitrogen resulted in a cessation of cell doubling (Additional file 1: Figure S1), while biomass concentrations continuously increased in total 6.7-fold until day 11 of the −N treatment. This is close to the reported 7.8-fold change of *Acutodesmus obliquus* (UTEX 393) after 13–14 days of −N conditions with 5% CO_2_ [38], highlighting the strong biomass productivity of *M. neglectum* [27]. The two stages were the early, starch accumulation stage (e−N stage) and the later, TAG and total lipid hyperaccumulation stage (l−N stage) (Figs. 1 and 2). The third stage was selected to allow investigation of the reverse reactions, when nitrogen-limited cells (48 h starvation period) were resupplied with the essential nutrient (r+N stage; Fig. 1). This alternating treatment allowed the analysis of both storage compound accumulation and subsequent degradation, respectively, which significantly increased the reliability of the interpretation of gene expression changes (see above and Additional file 1: Figure S2c–f). This is not only the first time that the transcriptome of *M. neglectum* has been sequenced, but also, to our knowledge, the first time that a microalgal transcriptome has been analyzed by mRNA-seq under both −N and N resupply conditions in a time course experiment to elucidate the molecular mechanisms of TAG accumulation. Although a previous study with the dinoflagellate *Karenia brevis* analyzed the effect of N re-addition using microarrays, this study had a different focus, because the authors were interested in determining if the transcriptome of *K. brevis* is responsive to nitrogen and phosphorus concentrations due to the prevalence of post-transcriptional regulation in dinoflagellates [83]. Accordingly, the effects of nutrient re-addition on individual pathways, such as TAG and lipid metabolism, were not investigated [83]. Furthermore, the transcriptome changes under −N conditions were not investigated in great detail [83].

We used the standard criterion of absolute log2-FC > 1 to define a gene as responsive to our treatment. This was supported by the expression profiles of housekeeping genes, which were classified as not-responsive under –N conditions according to this definition (Additional file 1: Fig. 2a). However, it should be noted that the importance of a gene with regard to a phenotypic effect is not necessarily directly correlated with the factor of up- or down-regulation, but can strongly depend on potential post-transcriptional regulation and on the function of the corresponding protein. In this context, protein kinases were specifically up-regulated in the l−N stage in *M. neglectum* (GO term “*protein phosphorylation*” in Additional file 1: Table ST1), which has also been reported for *C. reinhardtii* after 48 h of mixotrophic −N conditions [10], indicating that protein phosphorylation might be a factor for post-transcriptional regulation under −N conditions. With respect to protein function, a pronounced transcriptional regulation can be expected for structural proteins such as MLDP (Fig. 3c), because these are abundantly required in the cell to exert a specific function, in this case to determine the size of lipid droplets [58]. In contrast, enzymes involved, e.g., in the distribution of carbon flow can be expected to react more moderately, but still to an extent that allows sufficient protein synthesis to direct metabolic flux. Regulatory proteins such as transcription factors, however, might only show a low degree of transcriptional regulation, which could still be metabolically extremely important. Therefore, the FC-based threshold has the limitation that the classification of individual genes might not in all cases represent physiological importance. However, this classification was chosen because this study primarily focused on the interpretation of transcriptional regulation of enzymatic reactions to identify gene targets for subsequent genetic engineering approaches.

### Global characterization of the three stages of N availability on the level of the transcriptome by GO term enrichment analysis

To first characterize the individual stages of N availability on the transcriptome level, a GO term enrichment analysis of shared responsive genes was conducted (Fig. 3e, Additional file 1: Table ST1). As a result, biological processes were identified, which were subjected to transcriptional regulation in the three stages. The GO term enrichment profile of genes up-regulated continuously during the e−N and l−N stages is indicative of a transcriptional induction of the tricarboxylic acid cycle and glycolysis pathways. This has also been reported for other microalgae as a response to −N conditions, such as *N. oleoabundans* [17], *N. oceanica* [20], and *P. tricornutum* [19]. The induction of the tricarboxylic acid cycle under −N conditions might be bolstered by the consistent up-regulation of PEP carboxylase generating oxaloacetate from PEP (Fig. 6, PEPC), which might help regulate the flux through the tricarboxylic acid cycle by replenishing oxaloacetate. Alternatively, the up-regulation of PEP carboxylase could indicate that this route of CO_2_ fixation became increasingly important under −N conditions. The opposing transcriptional regulation of glycolysis (up-regulation) and gluconeogenesis (down-regulation) indicates that *M. neglectum* switched from a primarily gluconeogenetic to glycolytic state under −N conditions (Fig. 6), which has also been described for *C. reinhardtii*, *P. tricornutum*, and *N. oceanica* [10, 19, 20]. The enrichment profile of down-regulated genes in both the e−N and l−N stages consisted mostly of photosynthesis-associated processes, which has also been observed in *C. reinhardtii*, *N. oleoabundans*, *B. sudeticus*, *P. tricornutum*, and *N. oceanica* under −N conditions [11, 17, 19, 20, 23].

According to the GO term analysis, a biphasic response of the tetrapyrrole pathway for *M. neglectum* becomes apparent, because the transcriptional repression of chlorophyll biosynthesis preceded the transcriptional induction of chlorophyll catabolism in the e−N and the l−N stages, respectively (Additional file 1: Table ST1). A biphasic response of the tetrapyrrole pathway was also described for *C. reinhardtii* under mixotrophic –N conditions [11]. The enrichment profile of the e−N stage is indicative of a transcriptional induction of cell division (Additional file 1: Table ST1). This might simply be seen in accordance with the increase in cell concentration during the first 2 days of −N treatment (Additional file 1: Figure S1). Alternatively, in combination with the aforementioned down-regulation of chlorophyll biosynthesis in the e−N stage, this might reveal a transcriptional program that aims at diluting the cellular chlorophyll pool. This scenario has been described for *C. reinhardtii*, in which the decrease of cellular chlorophyll contents under mixotrophic −N conditions was not only due to cessation in chlorophyll synthesis, but also due to dilution by cellular growth [84].

The GO term “*microtubule*-*based processes*” was enriched among genes specifically up-regulated in the e−N stage (Additional file 1: Table ST1), which was attributed to seven genes in this set. Six of these encoded structural components of microtubules (tubulin), while the seventh encoded the microtubule motor protein dynein. The up-regulation of these seven genes might either be put into the aforementioned context of cell division. Alternatively, it might be interpreted as a hint towards a putative remodeling of the cytoskeleton as a preparatory step for subsequent lipid droplet formation for TAG storage. Microtubules play a role in directing MLDP to lipid droplets in *C. reinhardtii* [30]. Furthermore, cytoskeleton remodeling was required to elicit the obesity phenotype of high lipid transformants of the oleaginous yeast *Yarrowia lipolytica* [85].

The −N treatment aimed at the cessation of cell doubling as a result of inhibited protein biosynthesis. Therefore, we expected a transcriptional repression of cellular processes involved in protein biosynthesis, e.g., synthesis of ribosomal proteins. In accordance with this, we found that the GO term “*translation*” was enriched among genes down-regulated in the e−N stage (Additional file 1: Table ST1). Transcriptional repression of ribosomal proteins in response to –N treatment has also been noted in *C. reinhardtii* [11], *B. sudeticus* [23], *N. oleoabundans* [17], *P. tricornutum* [19], *N. oceanica* [20], and *N. gaditana* [21]. Interestingly, despite the general transcriptional repression of ribosomal proteins, the GO term analysis revealed that arginine biosynthesis was up-regulated in both the e−N and the l−N stages, whereas cysteine biosynthesis was down-regulated in both stages; in contrast, aromatic amino acid biosynthesis was specifically down-regulated in the e−N stage (Additional file 1: Table ST1). However, this might not necessarily translate into a metabolic effect, because no increased flux through arginine biosynthesis was detected under −N conditions in *C. reinhardtii*, despite a transcriptional up-regulation [22].

### Putative glycerolipid metabolism of *M. neglectum*

FA synthesis generates the acyl chains that can be used for glycerolipid synthesis (Fig. 4). The synthesis of C16 and C18 FAs is presumed to be exclusively plastidial in *C. reinhardtii* [8], which most likely also applies to *M. neglectum*. This was supported by localization prediction (Fig. 4), and by a comparison of overall transcript abundance values. Transcript levels were 13- to 46-fold higher for the heteromeric ACCase, compared to the homomeric ACCase (Additional files 3 and 4), which are characteristic for plastidial and cytosolic/mitochondrial FA synthesis, respectively. ACCase catalyzes the first committed step of FA synthesis and is considered a key rate-limiting enzyme [86]. The product of FA synthesis is acyl-ACP, which can directly be used for the synthesis of chloroplast membrane glycerolipids by sequential acylation of glycerol-3-phosphate [8]. Alternatively, acyl-ACP can be separated into the ACP moiety and a free FA by FAT [8]. It is postulated that the free FA is transported across the chloroplast membrane into the cytosol, where it is activated by CoA to acyl-CoA, catalyzed by LACS [8]. The activated acyl-CoA moiety can then be used for the synthesis of, for instance, ER membrane lipids [8]. This pathway could be completely reconstructed for *M. neglectum* (Fig. 4).

In higher plants, TAG synthesis is restricted to the ER [87], while in microalgae TAG synthesis does likely not only take place in the ER, but also in the chloroplast [8]. However, none of the putative DGAT, DGTT, and PGAT enzymes of *M. neglectum* were predicted to be chloroplast localized (Fig. 4). Therefore, localization studies are required to confirm whether TAG synthesis in *M. neglectum* is both chloroplast- and ER-localized, analogous to other microalgae.

For higher plants, PtdCho is a key intermediate for TAG synthesis [87]. For developing soybean embryos, it has been shown that 60% of newly synthesized FAs are incorporated directly into the *sn*-2 position of lyso-PtdCho yielding PtdCho, rather than being used for sequential acylation of glycerol-3-phosphate in the Kennedy pathway [88]. This reaction is catalyzed by lysophosphatidylcholine acyltransferase. An acyl chain can subsequently be released from PtdCho by different lipases, which can then be used for sequential acylation of glycerol-3-phosphate and TAG synthesis [88]. This pathway does not apply for the model green alga *C. reinhardtii*, because it lacks PtdCho [8]. However, it might apply to other microalgae to some extent, because genomic evidence for PtdCho synthesis was reported for *Chlorella pyrenoidosa* [42] and PtdCho has been detected in *N. oceanica* [89]. Transcript evidence for PtdCho was also detected for *M. neglectum*, because one CTP:phosphocholine cytidylyltransferase and two phosphatidylethanolamine N-methyltransferase homologues could be identified (Fig. 4, CCT and PEAMT, respectively), which are both absent from the *C. reinhardtii* genome [65]. CTP:phosphocholine cytidylyltransferase is considered as a key rate-limiting enzyme for de novo synthesis of PthCho, and phosphatidylethanolamine N-methyltransferase catalyzes an alternative route of PthCho synthesis, which is by methylation of phosphatidylethanolamine [90]. In *C. reinhardtii*, PthCho is replaced by DGTS [8]. Interestingly, the enzyme required for DGTS synthesis was also identified in the transcriptome of *M. neglectum* (Fig. 4, BTA). Accordingly, similarly to what has been recently suggested for *C. pyrenoidosa* [42], *M. neglectum* might be capable of both PthCho and DGTS syntheses, a hypothesis that has to be confirmed biochemically.

### The transcriptional regulation of the glycerolipid metabolism reveals several endogenous factors for TAG and total lipid hyperaccumulation

A first factor underlying TAG and total lipid hyperaccumulation in the l−N stage becomes evident from the transcriptional regulation of FA synthesis. Its regulation was not uniform in the l−N stage, in contrast to the r+N stage, in which almost all enzymatic steps of FA synthesis were uniformly transiently down-regulated (Fig. 4, ACCase, MCMT, KASI, KASII, KAR, HAD, ENR, ACP, and FAT). In the l−N stage, a strong transcriptional induction of FAT and a gentle induction of ACCase were observed (Fig. 4), while several intermediate steps of FA synthesis were repressed, although the extent was moderate compared to the r+N stage (Fig. 4, MCMT, KASIII, and KAR). At the same time, the transcript levels of ACP were maintained in the l−N stage, which were among the most abundant transcripts (Additional file 2). ACP prevents the growing acyl chain from degradation [91]. High expression of ACP is in accordance with data for *E. coli* showing that ACP belongs to the most abundant proteins [91]. As an abundant protein, however, a strong transcriptional down-regulation of ACP would be expected, if FA synthesis was significantly inhibited under −N conditions. Since this was not observed, but ACP transcript levels were maintained (Fig. 4), it is tempting to speculate that the down-regulation of intermediate enzymatic steps of FA synthesis (e.g., KASIII) results in a modulation of FA synthesis towards increased production of oleic acid (C18:1). Oleic acid is the most abundant FA in the neutral lipid fraction of *M. neglectum* [27, 28]. This is supported by studies for *E. coli* that reported increased and decreased levels of C18 species upon deletion and over-expression of 3-ketoacyl synthase III (KASIII, *fabH*), respectively, at the expense of C16 FAs [92, 93]. In accordance with this, the gentle transcriptional induction of ACCase might aim at replenishing the pool of malonyl-CoA required for FA elongation. The transcriptional repression of the immediate downstream step might seem contradictory (Fig. 4, MCMT); however, both genes had approximately equal expression levels after 56 h of −N conditions (Additional file 3, MCMT, XLOC_016566, 175 FPKM and β-CT, XLOC_015237, 177 FPKM, respectively). Accordingly, the gentle transcriptional repression of MCMT in the l−N stage might aim at balancing both enzyme levels.

A second factor can be deduced from the transcriptional regulation of FAT and SAD. FAT cleaves ACP from acyl-ACP yielding a free FA, and SAD catalyzes the desaturation of stearic acid (C18:0) to oleic acid (C18:1). The transcriptional up-regulation of both genes might result in a deregulation of FA synthesis. This is because in plants and bacteria acyl-ACP has an inhibitory effect on ACCase [91, 94]. Accordingly, up-regulation of FAT in *M. neglectum* might decrease the pool of inhibitory acyl-ACP, thereby relieving feedback inhibition and in turn keeping FA synthesis in an active state. SAD was postulated to be a metabolic lipid regulator in mammals and shown to co-limit lipid production in oleaginous yeast [85]. In *C. reinhardtii*, SAD over-expression was reported to increase the total lipid content by approximately 28% [95].

A third factor becomes evident from the transcriptional regulation of the putative PGD1 lipase in *M. neglectum* (Fig. 4). For *C. reinhardtii*, it was shown that de novo TAG accumulation involves a significant flux through the membrane lipid pool [73]. In this scenario, rather than direct channeling of de novo synthesized FAs into TAG synthesis, at least some are first incorporated into membrane lipids, from which free FAs are released by the action of PGD1, which are in turn incorporated into TAGs [36, 73]. This indirect channeling of FAs into TAGs through an intermediate membrane lipid step likely also applies to *M. neglectum*, because PGD1 and other lipases were strongly up-regulated (Fig. 5), and the GO term “*lipid catabolic process*” was enriched among genes up-regulated specifically in the l−N stage (Additional file 1: Table ST1). Interestingly, the pronounced transcriptional up-regulation of the PGD1 lipase was reserved for the l−N stage (Fig. 4). This suggests that PGD1 has a specific role in de novo TAG accumulation, because acyl chain recycling from membrane lipid degradation was finished after two days (Fig. 2c). The transcriptional regulation of PGD1 furthermore indicates that some acyl-shuttling processes, for instance those involving PGD1, are less relevant in N-containing media, because PGD1 induction was immediately relaxed in the r+N stage (Fig. 4). In accordance with a possibly diminished pool of lyso-glycerolipids during the first hours of N resupply, one lysophosphatidylcholine acyltransferase candidate was transiently down-regulated in the r+N stage (XLOC_009408, LP-C/E-AT in Fig. 4).

The strong transcriptional regulation of PGD1 led us to explore the transcriptional profiles of further lipase candidate genes (Fig. 5). Notable examples of lipases that were also up-regulated in the l−N stage are XLOC_011377, XLOC_001858, and XLOC_013922 (Table 1; Fig. 5). Interestingly, the homologues of XLOC_001858 and XLOC_013922 were also up-regulated in *C. reinhardtii* under −N conditions, indicating potentially conserved functions (Cre09.g388763, 6e−65; Cre03.g195500, 1e−36; transcript data in [11]). A highly interesting lipase candidate is XLOC_013518, a putative patatin-like phospholipase (Table 1). Under exponential growth conditions, its transcript levels were the highest among all lipases (Fig. 5, XLOC_013518, category IV). Under −N conditions in contrast, this gene was down-regulated to an extent that expression was hardly detectable (Fig. 5, XLOC_013518, tag “NA” at time points N_48, N_56, and N_96). In the r+N stage, initial transcript abundance values were restored after 14 h of N resupply (Fig. 5, XLOC_013518, R_14). This relatively late relaxation of repression in the r+N stage indicates that this lipase is unlikely a TAG lipase, which otherwise should be transcriptionally induced early in the r+N stage. Therefore, this protein could be involved in preserving membrane lipid integrity under exponential growth conditions, although postulation of its function is complicated, because homologues could be identified neither in *C. reinhardtii*, nor in *A. thaliana*.

Interestingly, the putative CTP:phosphocholine cytidylyltransferase enzyme characteristic for PthCho synthesis was up-regulated in the l−N stage in *M. neglectum* (Fig. 4, CCT). Considering that in developing soybean embryos DAG moieties used for TAG synthesis were mostly derived from PthCho [88], this transcriptional induction might be indicative of a similar flux pattern in *M. neglectum*. Consequently, a considerable amount of TAGs would be obtained via the PthCho route, rather than via sequential acylation of glycerol-3-phosphate. However, flux analyses are required to investigate how far this model applies to *M. neglectum*.

The strong transcriptional induction of FAT, PGD1, and other lipases under −N conditions likely yields an elevated pool of free FAs, which need to be activated by CoA to acyl-CoA, before they can be incorporated into glycerolipids, such as TAGs. This activation is catalyzed by LACS enzymes, and a central role of LACS in TAG accumulation became recently evident for *C. reinhardtii*, because loss of LACS2 decreased TAG content by 50% [96]. In *M. neglectum*, eleven LACS transcripts were identified, and those LACS enzymes with increased transcript abundances under −N conditions might play a role in FA activation for subsequent incorporation into TAGs (e.g., XLOC_003478), whereas those with increased abundances upon N resupply might activate FAs for subsequent β-oxidation (e.g., XLOC_002550) (Fig. 4).

### Transcriptional regulation of the acylation of DAG, the committed step of TAG synthesis

The committed step in the acyl-CoA-dependent pathway of TAG formation is catalyzed by DGAT and DGTT. Although transcriptional induction of DGTT transcripts was observed under −N conditions in *M. neglectum*, induction was moderate compared to *C. reinhardtii* (>100-fold induction for DGTT1 [10]) and *N. oceanica* (5.7-fold induction for DGAT-2B [20]). The only putative DGTT gene that was strongly up-regulated in −N conditions surprisingly was also strongly induced under N resupply, a profile that is not expected for an enzyme involved in TAG accumulation (Fig. 4, sixth DGTT transcript from top). A gentle transcriptional induction of DGAT/DGTT genes under −N conditions was reported for *N. oleoabundans* and *P. tricornutum* [17, 19]. Accordingly, other regulation levels than transcriptional control might be more relevant for DGAT/DGTT-mediated TAG accumulation in *M. neglectum*. This might also be an explanation of the limited success of DGTT over-expression to increase TAG accumulation in *C. reinhardtii* [97, 98]. Interestingly, transcript levels of PDAT, characteristic for the acyl-CoA independent route of TAG formation, were approximately constant under −N conditions (Fig. 4). A gentle induction of ~twofold has been reported for *C. reinhardtii* [12] and *P. tricornutum* [19] under −N conditions, and a marginal up-regulation of ~0.5-fold for *N. oceanica* [20]. Accordingly, the extent of transcriptional regulation of PDAT was similar for *M. neglectum*, indicating that PDAT may contribute to a similar extent to TAG formation in *M. neglectum* as has been reported for *C. reinhardtii* [12]. In summary, it seems likely that the predominant way of TAG formation in *M. neglectum* is by the acyl-CoA-dependent pathway, rather than by the acyl-CoA independent pathway, as indicated by the strong transcriptional induction of putative FAT, PGD1, and lipase enzymes, as well as by the gentle induction of putative LACS and DGTT enzymes under −N conditions, while PDAT was approximately constantly expressed (Fig. 4).

### Sources of acetyl-CoA for FA synthesis in the l−N stage

Acetyl-CoA is the carbon precursor for FA synthesis. There are several routes for acetyl-CoA formation, and two are considered the most important in chloroplasts of higher plants [87]. Those are the oxidative decarboxylation of pyruvate catalyzed by the multisubunit cpPDHC (plastidial pyruvate dehydrogenase complex) and, to a lesser extent, the activation of free acetate to acetyl-CoA by acetyl-CoA synthetase [87]. Acetate can be obtained by fermentative decarboxylation of pyruvate catalyzed by pyruvate decarboxylase. Alternative fermentative reactions directly yield acetyl-CoA from pyruvate, such as those catalyzed by pyruvate-formate-lyase and pyruvate-ferredoxin-oxidoreductase. In microalgae, the preferred route of acetyl-CoA generation for FA synthesis under −N conditions has not yet been determined, but is presumed to be via cpPDHC [99], analogous to higher plants [87].

The transcriptional regulation of the putative cpPDHC subunits in *M. neglectum* was outlined in the Results section (Additional file 1: Figure S2e) and suggests that cpPDHC activity might be approximately maintained under −N conditions (Fig. 6). High transcript abundances for cpPDHC were noted (Fig. 6, category IV), indicating that cpPDHC might contribute significantly to the plastidial acetyl-CoA pool. A strong transcriptional induction in the l−N stage was observed for fermentative reactions, which was immediately relaxed upon N resupply (Fig. 6, PFOR, PFL, PDC, and ADH). This might have two effects. First, the corresponding enzymes might increase acetyl-CoA supply in the l−N stage, yet likely to a lesser extent than cpPDHC, because transcript levels in the l−N stage of the former were lower than those of the latter (Additional file 3). Second, the transcriptional induction might aim to optimize the flux of pyruvate reaching cpPDHC, in order to maintain optimal acetyl-CoA production rates by cpPDHC to ensure sufficient precursor supply for FA synthesis, as has been postulated for *Chlorella desiccata* [100, 101].

### Transcriptional regulation of two enzymatic steps in the l−N stage of the central carbon metabolism might result in a re-routing that fuels into fatty acid synthesis

The most abundant FA of the neutral lipid fraction in *M. neglectum* is oleic acid (18:1) [27, 28], and its synthesis requires 19 NADPH molecules (18 for the C18 acyl chain, and an additional molecule for the subsequent desaturation of stearic acid to oleic acid). Accordingly, FA synthesis requires a high supply of NADPH. A first pathway for NADPH regeneration exists with the linear electron flow in photosynthesis [102]. The OPPP provides two NADPH molecules per glucose 6-phosphate molecule and was predicted to be localized to the chloroplast of *M. neglectum*, as has been described for *C. reinhardtii* [32]. Our data, obtained on the transcriptional level, are feasible to postulate that under −N conditions the transcriptional induction of the OPPP (Fig. 6) offers the opportunity to provide an additional pathway for the delivery of NADPH. Transcriptional induction of the OPPP under −N conditions has also been reported for *C. reinhardtii* and *N. oleoabundans*, which correlated with increased protein abundances in *C. reinhardtii* [103], and has been interpreted similarly [13, 14, 17, 103]. In this context, by analyzing the response of the starchless mutant *sta6* of *C. reinhardtii* under mixotrophic −N conditions, it was proposed that increasing the reductant pool is a promising strategy to increase lipid productivity [14]. This would be contradictory to the overflow hypothesis, according to which TAGs are “overflow products,” i.e., to serve as a sink for excess photosynthetic energy [104]. However, it was recently elaborated that this hypothesis is insufficient for carbon compound accumulation [36, 84].

Phosphoglycerate kinase catalyzes the interconversion between 3-phosphoglycerate and 1,3-bisphosphoglycerate. Both phosphoglycerate kinase candidate genes of *M. neglectum* were strongly down-regulated in the l−N stage (Fig. 6, PGK). This was also observed in *C. reinhardtii* [11], which correlated with decreased protein abundances in [103] but not in [11]. Assuming that the transcriptional repression of phosphoglycerate kinase under −N conditions in *M. neglectum* manifests on the metabolic level, a redirection of the carbon flow would be obtained. Accordingly, carbon flow might be shifted away from the formation of 1,3-bisphosphoglycerate for replenishment of the Calvin cycle, towards the production of 2-phosphoglycerate, such that carbon flow is directed to pyruvate generation by subsequent glycolytic reactions. The down-regulation of phosphoglycerate kinase on the transcript level might thus represent an important determinant to redirect fixed carbon towards pyruvate synthesis, and was also observed in other microalgae under −N conditions (Additional file 1: Results). However, although the expression patterns clearly indicate this alternative metabolic route, labeling studies would be required to investigate how far they reflect the metabolic flux in *M. neglectum* under −N conditions.

### Transcriptional regulation of glycerol-3-phosphate supply for TAG biosynthesis

Glycerol is the backbone of TAG and other glycerolipids. Glycerol-3-phosphate is obtained from the reduction of dihydroxyacetone phosphate, catalyzed by glycerol-3-phosphate dehydrogenase (Fig. 6). Three glycerol-3-phosphate dehydrogenase candidate transcripts were identified in the transcriptome of *M. neglectum* (Additional file 4). One of these had predicted chloroplast localization and was highly up-regulated in response to N starvation (Fig. 6, GPDH). A strong transcriptional induction of a subset of glycerol-3-phosphate dehydrogenase enzymes has also been noted for *C. reinhardtii* [13]. In rape (*Brassica napus*), glycerol-3-phosphate supply was proposed to co-limit oil accumulation, because over-expression of a yeast glycerol-3-phosphate dehydrogenase increased seed oil content [105]. In the diatom *P. tricornutum*, over-expression of glycerol-3-phosphate dehydrogenase increased the neutral lipid content by 60% [106]. Therefore, the respective up-regulated enzyme of *M. neglectum* (XLOC_014979) might have a central role in TAG accumulation and represents a promising target for genetic engineering approaches (Table 1). However, over-expression of glycerol kinase to increase glycerol-3-phosphate supply in the diatom *Fistulifera solaris* was of mixed success, because the total lipid content was both increased and decreased in a first and a second transformant, respectively, under autotrophic as well as mixotrophic conditions (external glycerol supply) [107].

### Transcriptional regulation of malic enzyme in *M. neglectum* suggests that this reaction likely does not have a central role in lipid hyperaccumulation in the l−N stage

A central component of lipid accumulation in oleaginous fungi is the induction of ATP-citrate lyase and of malic enzyme. ATP-citrate-lyase converts citrate into acetyl-CoA and oxaloacetate. Oxalacetate can be converted to malate by malate dehydrogenase. Malic enzyme decarboxylates malate to the central intermediate pyruvate, and this reaction additionally provides NADPH [108–110]. We note that there is a significant difference between oleaginous fungi and microalgae, because the former are obligate heterotrophs, thus lacking chloroplasts, while most microalgae are capable of growing photoautotrophically. A key difference therefore is the localization of FA synthesis, which generally takes place in the cytosol in non-photosynthetic species and in the chloroplast in eukaryotic photosynthetic species.

Interestingly, citrate was shown to accumulate under −N conditions in *C. reinhardtii,* and its exogenous supply increased lipid accumulation [111]. In *M. neglectum* however, transcript levels of ATP-citrate lyase were approximately constant under −N conditions (Fig. 6, ACL), suggesting that this reaction is not a preferred route for acetyl-CoA generation in *M. neglectum*.

In the diatom *P. tricornutum*, one of the most strongly up-regulated genes under −N conditions was malic enzyme [19], and its subsequent over-expression in both *P. tricornutum* (homologous expression) and the chlorophyceae *C. pyrenoidosa* (heterologous expression) was claimed to increase total lipid contents by 230–250 and 240–322% in the stationary phase, respectively [112, 113]. However, transcriptional regulation and absolute abundances of malic enzyme in *M. neglectum* in the l−N stage were modest (Fig. 6, MME), suggesting that malic enzyme does not play a major role during lipid accumulation in this stage. This is in accordance with a metabolome study of *Chlorella protothecoides* concluding that malic enzyme had little to no activity under both +N and −N conditions despite robust lipid accumulation under −N conditions [114].

### Enolase and C3 carbon transporters are subjected to differential transcriptional regulation in response to alternating nitrogen availability

Enolase catalyzes the reversible conversion of 2-phosphoglycerate to PEP and was recently postulated to represent an important determinant for carbon flux in chlorophyceae [115]. Two putative enolase transcripts were identified in the transcriptome of *M. neglectum*, which had both increased abundances under −N conditions (Fig. 6, ENO). Interestingly, a gentle up-regulation (~twofold) of enolase could also be found in the oleaginous chlorophyceae *N. oleoabundans* after 11 days of −N conditions (transcript data in [17]), whereas transcript levels in the non-oleaginous chlorophyceae *C. reinhardtii* and *B. sudeticus* remained approximately constant during 48 and 72 h of −N conditions, respectively (transcript data in [11, 23]). Accordingly, the transcriptional induction of enolase in *M. neglectum* could potentially be of importance for its oleaginous phenotype, which would be interesting to investigate in future studies.

Besides, two of 15 putative triose phosphate transporters were up-regulated under −N conditions, of which one was predicted to localize to the chloroplast (Fig. 6, TPT). In contrast, five different triose phosphate transporters were transiently down-regulated in the r+N stage, of which one with high expression under initial exponential growth conditions was predicted to be chloroplast localized (Fig. 6, TPT). Furthermore, a putative PEP transporter was strongly down-regulated in the e −N stage, while this down-regulation relaxed in the l −N stage, and a strong up-regulation was subsequently noted for the r+N stage (Fig. 6, PPT). This indicates a differential transporter composition of, for instance, the chloroplast membrane under both N regimes and suggests a possibly important role of carbon transport in the response to alternating N availability.

### Thylakoid membrane lipid synthesis is likely stimulated on two different levels during the N resupply treatment

A central characteristic of the r+N stage was the reestablishment of photosynthesis, including thylakoid membrane reassembly (Additional file 1: Table ST1, Figure S1). The major thylakoid membrane lipid of *C. reinhardtii* is MGDG [8]. MGDG is derived from DAG by galactosylation, catalyzed by MGDG synthase [8]. MGDG synthesis in the r+N stage in *M. neglectum* might be stimulated on two different levels: first on the transcript level, because the respective gene was up-regulated in the r+N stage (Fig. 4, MGDGS); second, the transient transcriptional repression of two of three phosphatidic acid phosphatase candidate genes in the r+N stage might reduce the turnover of phosphatidic acid to DAG (Fig. 4, PAP), which would result in a transient accumulation of phosphatidic acid in this stage. Accumulation of phosphatidic acid was postulated to hyperstimulate MGDG synthase activity in *C. reinhardtii* [116]. Therefore, the second level of stimulation of MGDG synthesis in the r+N stage in *M. neglectum* might be on the metabolic level by the putative transient accumulation of phosphatidic acid. Although reduced turnover would also decrease the availability of de novo DAG for MGDG synthesis, it seems unlikely that this limits MGDG synthesis, since the overall DAG pool in the r+N stage should be saturated due to TAG hydrolysis yielding DAG moieties.

### The restoration of photosynthetic efficiency upon N resupply involves several steps with different timings

The immediate transcriptional responses of *M. neglectum* in the r+N stage were the up-regulation of TAG hydrolysis, membrane lipid synthesis, and FA degradation, as well as the concomitant down-regulation of FA synthesis, which were most pronounced after 2 h of N resupply (Fig. 4, ACX, MGDGS, and ACCase, respectively, Fig. 5). Next, the transcriptional repression of light-harvesting protein genes that was detected during the complete −N phase relaxed after 4 h of N resupply (Additional file 3, XLOC_000814, XLOC_006556, XLOC_016372, and XLOC_003245). Finally, the transcriptional repression of the Calvin cycle under −N conditions relaxed after 8 to 14 h of N resupply (Fig. 6, rbcS2 and PGK). Taken together, this indicates that during N resupplementation the reestablishment of full photosynthetic capacity takes place in a coordinated manner and in temporally different patterns.

### Metabolic engineering strategies to optimize TAG production in *M. neglectum*

A major goal of this differential transcriptome study was to understand the transcriptional basis for TAG accumulation and TAG degradation in *M. neglectum*, in order to identify potential gene targets for future metabolic engineering approaches. The most promising candidates are summarized in Table 1. One option is to relieve feedback inhibition of FA synthesis by the over-expression of chloroplast-targeted FAT (acyl-ACP thioesterase) genes, a strategy that has been successfully applied before in *P. tricornutum* [117], *C. reinhardtii* [118], and other photosynthetic [119] and heterotrophic species [91, 120]. Since in this work a clear correlation between transcriptional up-regulation of both putative FAT enzymes and lipid hyperaccumulation was observed (Figs. 2c and 4), over-expression of these genes represents a prime target. Heterologous expression of FAT from a closely related species, such as *C. reinhardtii* (Additional file 1: Figure S12), might also be of interest, in order to circumvent possible species-specific regulations of the endogenous enzymes.

**Table 1:** List of potential gene targets for genetic engineering approaches to increase lipid production in *M. neglectum*

Annotation	Locus ID	Putative enzyme function	Transcript profile	Approach	Postulated effect	References in other microalgae
Acyl-ACP thioesterase (FAT, Fig. 4)	XLOC_007529 or XLOC_011123	Cleaves acyl-ACP into a free FA and ACP, thus relieving feedback inhibition of FA synthesis; requires functional interaction with ACP [148]	Up-regulated in the l−N stage, down-regulated in the r+N stage	Over-expression	Deregulation of FA synthesis allowing for total lipid hyperaccumulation under +N conditions	[91, 117–120]
Lipases, such as PGD1 (Fig. 5)	XLOC_12515 (PGD1) or XLOC_011377 or XLOC_013518	XLOC_12515: lipase that may act on membrane lipids such as de novo MGDG XLOC_011377: unknown function and no domain predicted; XLOC_013518: unknown function with a predicted patatin_cPLA2 superfamily domain	XLOC_12515 and XLOC_011377: both are strongly up-regulated in the l−N stage; expression of XLOC_011377 was highly correlated to MLDP (>0.99) XLOC_013518: abundant transcript; strongly repressed in the l−N stage, and repression was continued during the first 8 h of N resupply	Over-expression (XLOC_12515 and XLOC_011377) Knock-down (XLOC_013518)	XLOC_12515 and XLOC_011377: membrane lipid turnover putatively triggering TAG accumulation under +N conditions XLOC_01351: might be involved in maintaining membrane lipid integrity; down-regulation could lead to an increased TAG content under +N conditions	[73, 121]
Acyl-CoA oxidase (ACX2, Fig. 4)	XLOC_015398	Implicated in FA degradation by oxidizing acyl-CoA	Up-regulated in the r+N stage	Knock-down, deletion	Increased TAG content by reducing the rate of FA degradation (repression of catabolic pathways)	[31]
Phosphoglycerate kinase (PGK, Fig. 6)	XLOC_018937	Converts 3-phosphoglycerate to 1,3-bisphosphoglycerate and vice versa	Strongly repressed in the l−N stage, which was continued for the first 4 h in the r+N stage	Knock-down	Redirecting carbon flow towards pyruvate generation, away from glyceraldehyde-3-phosphate for replenishment of the Calvin cycle	NA
PEP carboxylase (PEPC, Fig. 6)	XLOC_004101	Carboxylation of PEP to oxalacetate	Up-regulated under −N conditions; approximately unaltered transcript levels in the r+N stage	Knock-down	PEP can be increasingly used for pyruvate generation, rather than for replenishment of the tricarboxylic acid cycle	[149–151]
Glycerol-3-phosphate dehydrogenase (GPDH, Fig. 6)	XLOC_014979	Converts dihydroxyacetone phosphate into glycerol-3-phosphate	Up-regulated under −N conditions	Over-expression	Increased TAG accumulation due to increased supply of glycerol-3-phosphate under −N conditions	[106]
Enolase (ENO, Fig. 6)	XLOC_012275	Converts 2-phosphoglycerate to PEP	Up-regulated under −N conditions	Over-expression	Possibly altered carbon partitioning towards increased PEP generation under +N conditions	[115]
Transcription factor (Additional file 5)	XLOC_013389 or XLOC_005581	XLOC_013389: transcription factor of MYB family XLOC_005581: transcription factor of GATA family	XLOC_013389: strongly up-regulated under –N conditions; XLOC_005581: strongly repressed under –N conditions	Over-expression (XLOC_013389), knock-down (XLOC_005581)	Mimicking parts of the transcriptional regulation from −N conditions under +N conditions	[125, 126]
Small subunit of RuBisCo (rbcS2) or elongation factor or ribosomal protein (RPL7aE) (Additional file 1: Figure S2b)	XLOC_007679 or XLOC_005939 or XLOC_000987	XLOC_007679: rbcS2, central enzyme of photosynthesis catalyzing carbon fixation XLOC_005939 and XLOC_000987: implicated in protein biosynthesis	XLOC_007679: the third highest rate of expression under +N conditions, and only moderately affected by the −N treatment; the first intron could putatively contain an enhancer motif as in [152] XLOC_005939 and XLOC_000987: very high and stable expression under −N conditions	Cloning	Strong constitutive promoter for both +N and −N conditions	[152, 153]

The table is sorted according to pathways, starting with the glycerolipid metabolism, followed by the central carbon metabolism, finishing with other targets

Furthermore, lipases represent promising targets, because they are implicated in different processes of the glycerolipid metabolism, such as membrane lipid turnover and TAG degradation under −N and N resupply conditions, respectively [72, 73]. In accordance with a central role of lipases in the glycerolipid metabolism, the lipid content of the diatom *Thalassiosira pseudonana* could be increased 2.4- to 4.1-fold upon *knock*-*down* of a multifunctional lipase/phospholipase/acyltransferase enzyme under both nutrient replete and nutrient starvation conditions [121]. Interestingly, to our knowledge, over-expression approaches of lipases were not yet explored for microalgae. Therefore, both *knock*-*down* and over-expression approaches seem highly interesting for *M. neglectum*. Several putative lipase genes were transcriptionally regulated in opposite directions under −N and N resupply conditions in *M. neglectum* (Fig. 5), characterizing them as highly promising targets.

An alternative approach for increased TAG accumulation would be the repression of competitive catabolic pathways such as FA degradation, i.e., β-oxidation. In *C. reinhardtii*, disruption of one acyl-CoA oxidase gene greatly impaired the rate of β-oxidation, resulting in a 20% increased TAG content under −N conditions [31]. A putative homologue of the respective gene was identified in the transcriptome of *M. neglectum* (XLOC_015398). It exhibited a sharp transient up-regulation in the r+N stage (Fig. 4, ACX), suggesting that it is actively involved in FA degradation, therefore representing another prime target for gene *knock*-*down*/*knock*-*out* strategies. Alternatively, complete loss of peroxisomes by deletion of a peroxisome biogenesis factor was demonstrated to exhibit a cooperative effect on lipid hyperaccumulation in the heterotrophic host *Y. lipolytica* [122].

Investigations of the starchless *C. reinhardtii* strain *sta6* [123] have indicated that carbon precursor supply is not a limiting factor that prevents TAG accumulation under +N conditions. Compared to the parental strain, 18- and 27-fold increased intracellular malonyl-CoA levels were reported for the *sta6* strain under photoautotrophic +N conditions with low and high light intensities, respectively [124]. Cellular lipid levels, however, were similar to the parental strain [124]. Therefore, a currently unknown “−N signal” (or multiple “−N signals”) seems to be decisive for TAG accumulation, while carbon precursor supply augments TAG accumulation, once the “−N signal” is established. Accordingly, transcription factors are additional gene targets. The effectiveness of transcription factor over-expression was recently demonstrated with PSR1 (phosphorus starvation response 1) in *C. reinhardtii*, resulting in a “liporotund phenotype,” which exhibited lipid levels under +N conditions similar to the parental strain under −N conditions [125]. A *knock*-*down* strategy targeting a transcription factor was similarly effective, which doubled lipid content and volumetric lipid productivity under autotrophic +N conditions in *N. gaditana* [126]. Several transcription factors of *M. neglectum* showed clear transcriptional responses to alternating N availability, defining them as promising gene targets (Additional file 1: Results, Additional file 5). Although their efficient over-expression might be challenging because of the large size of transcription factor genes (e.g., 5191 bp for XLOC_013389), a strategy has been recently described that allows high expression of large transgenes [127]. It includes an even distribution of regulatory introns into the transgene [127], and this approach might be also effective for *M. neglectum*, because *M. neglectum* has a similarly intron-rich CDS composition as *C. reinhardtii* (Additional file 1: Results).

An interesting further gene target would be a chloroplast-targeted phosphoketolase [128]. This enzyme is absent from *M. neglectum* and other microalgae, except for *P. tricornutum* [129]. The xylulose-5-phosphate type of phosphoketolase catalyzes the cleavage of xylulose-5-phosphate into acetyl-phosphate and glyceraldehyde-3-phosphate [130]. Xylulose-5-phosphate is an intermediate of the Calvin cycle, which has a high flux under autotrophic conditions, and was reported to accumulate under mixotrophic −N conditions in *C. reinhardtii* [11]. Of the two products, acetyl-phosphate is the substrate for phosphotransacetylase, which generates acetyl-CoA that can be used for FA synthesis. Compared to the classical route of pyruvate-to-acetyl-CoA conversion, the phosphoketolase–phosphotransacetylase route does not involve a decarboxylation step, which increases the efficiency of carbon use. The over-expression of phosphoketolase and phosphotransacetylase in an engineered strain of the oleaginous yeast *Y. lipolytica* did not only increase lipid content to >60% of dry biomass, but importantly also uncoupled lipid accumulation from nutrient deprivation [131]. Although one candidate for phosphotransacetylase was identified in the transcriptome of *M. neglectum* (putative fragments XLOC_019320, XLOC_019178, and XLOC_011051), which was transcriptionally coherently regulated with β-oxidation in the r+N stage (Fig. 6, PTA), its transcript levels were low (FPKM = 4–14). Therefore, a two-gene approach, i.e., combined over-expression of both phosphoketolase and phosphotransacetylase, is likely necessary to establish an efficient metabolic shortcut for acetyl-CoA formation from xylulose-5-phosphate in *M. neglectum*.

## Conclusions

This study for the first time provides a comprehensive overview of the transcriptome responses to three different stages of N availability in the oleaginous chlorophyceae *M. neglectum*. The three stages early −N, late −N, and N resupply (e−N, l−N, and r+N stage; Fig. 1) were characterized by net starch accumulation, net lipid and TAG accumulation, and subsequent storage compound degradation, respectively (Fig. 2b, c; Additional file 1: Figure S1). The explorative analysis of transcriptional profiles was focused on the lipid and central carbon metabolism, and the starch metabolism was included as an associated pathway (Figs. 4 and 6; Additional file 1: Figure S10b). The transcript data were first used to refine the structural genome annotation of *M. neglectum* (Additional file 1: Results). Next, the quantitative evaluation of transcript data revealed that distinct putative lipases exhibited contrastingly different expression patterns (Fig. 5). Accordingly, the transcript profiles of lipases made it possible to distinguish candidates putatively involved in TAG accumulation from those likely involved in TAG degradation, which were up-regulated under −N and N resupply conditions, respectively (Fig. 5). The GO term enrichment analysis revealed a transcriptional induction of the tricarboxylic acid cycle and of glycolysis under −N conditions in the e−N and l−N stages (Additional file 1: Table ST1). Three main factors for lipid hyperaccumulation in the l−N stage could be deduced in *M. neglectum*. Those were (a) increasing carbon precursor supply, (b) relieving feedback inhibition of FA synthesis, and (c) remodeling of membrane lipid homeostasis, suggested by transcriptional induction of heteromeric ACCase, acyl-ACP thioesterase, and lipases such as PGD1, respectively (Fig. 4). A major goal of this work was the identification of target genes for future metabolic engineering approaches, and the detailed transcriptome analysis of *M. neglectum* cells during lipid accumulation and subsequent lipid remobilization stages allowed identification of several potential targets, which are summarized in Table 1. Reverse genetic approaches can now be performed to test the concept that altered expression of these target genes indeed leads to improved lipid accumulation phenotypes in *M. neglectum*.

## Methods

### Cultivation conditions

To define the timing of starch and lipid accumulation in *Monoraphidium neglectum* (SAG 48.87) and accordingly subdivide the −N phase in the e−N and l−N stages for subsequent transcriptome analysis, a long-term −N experiment was performed (Fig. 1, exp1). For this purpose, 900 mL ProF medium was inoculated with 10 mL of a mixotrophic growing pre-culture and grown at room temperature (24 °C) under autotrophic conditions with 3% CO_2_ bubbling with gentle stirring under 350–400 µmol m^−2^ s^−1^ constant illumination with white light from both the front and back sides until early mid-logarithmic phase was reached (~10 × 10^6^ cells mL^−1^). Cells were washed twice with N-free ProF and adjusted to ~3 × 10^6^ cells mL^−1^ with N-free ProF medium in a total volume of 2.8 in 3-L vertical Schott bottles. Cultivation was performed for 17 days under autotrophic conditions as above. After 1, 2, 4, 6, 8, 11, 14, and 17 days, 200 mL was removed for sample analysis. Three biological replicates were performed. Cell concentration was determined with a Z-series Coulter Counter cell and particle counter (Beckmann Coulter) and dry biomass according to [28].

For the transcriptome experiment (Fig. 1, exp2), *M. neglectum* was inoculated from agar plates and grown in 2.5 L Provasoli-based minimal media (ProF) [132] in 3-L vertical Schott bottles (Schott, USA) in two biological replicates. Cultivation was performed at room temperature (24 °C) under autotrophic conditions with 3% CO_2_ bubbling with gentle stirring under 350–400 µmol m^−2^ s^−1^ constant illumination with white light from only the front side until late exponential phase (40 × 10^6^ cells mL^−1^) was reached. Both cultures were diluted to ~4 × 10^6^ cells mL^−1^ with fresh ProF medium in a total volume of 2.5 L. Cultivation was continued for 2 days at 350–400 µmol m^−2^ s^−1^ constant illumination with white light from both the front and back sides, until a cell concentration of ~10 × 10^6^ cells mL^−1^ was reached.

From these cultures, samples for the time point zero (N_0) were taken to represent the untreated exponential growth phase. For N starvation treatment (“N”), the cells were centrifuged and washed twice with N-free ProF medium, resuspended in this medium to a cell concentration of ~4 × 10^6^ cells mL^−1^ in a total volume of 2.5 L, and cultivated under the same conditions for 4 days. Samples for RNA isolation were taken after 2, 4, 8, 24, 48, 56, and 96 h of −N conditions constituting the transcriptome sampling time points “N_0, N_2, N_4, N_8, N_24, N_48, N_56, and N_96,” respectively. Samples of 50–150 mL for lipid isolation were taken after 14, 24, 48, and 96 h of −N treatment.

For N resupply treatment (“R”), an aliquot of the starved cultures was removed after 48 h of −N treatment, washed with ProF, and adjusted to ~4 × 10^6^ cells mL^−1^ with fresh ProF medium (containing N), and cultivation was continued for an additional 2 days. Samples for RNA isolation were taken after 2, 4, 8, and 14 h, yielding the transcriptome sampling time points “R_2, R_4, R_8, and R_14,” respectively. Additional samples for lipid isolation were taken after 14, 24, and 48 h of N resupply. Cell concentration was determined microscopically with a hemocytometer and cell dry biomass according to [28].

Cell weight was obtained by dividing the biomass concentration by cell concentration.

### Lipid extractions and chromatography

Lipid extractions and chromatography were performed from 30 to 50 mg lyophilized biomass according to [28]. For calculation of the net lipid production rate, the volumetric lipid content at day 0 was subtracted from the volumetric lipid content at day X and this value was divided by the cultivation time, yielding the volumetric lipid productivity. These values were normalized to day 1 to retrieve the relative volumetric lipid productivity.

### Isolation of total RNA and DNase digest

For RNA isolation, the biomass was resuspended in 2 mL RNA extraction buffer (1:1 mix of aqua-phenol and buffer L [0.5% SDS, 10 mM EDTA, 0.2 M sodium acetate (pH 5), and 1:100 β-mercaptoethanol)]. Samples were frozen in liquid nitrogen and subsequently lysed with a Ribolyzer (PRECELLYS 24, Precellys, France) with three cycles of 45 s at 6500 rpm with 15-s break and 0.3 mL silica beads (diameter 0.1 mm). The lysate was incubated on ice for 10 min and 0.5 volumes of chloroform added. For phase separation, samples were centrifuged at 3000×*g* for ≥30 min and 16 °C. The upper phase was transferred to a new tube and treated with DNase I to remove contaminating DNA according to the manufacturer’s instructions (Promega, USA). The solution was extracted with equal volumes of aqua-phenol and chloroform for a second time, and a third time with an equal volume of chloroform only. RNA in the upper phase was precipitated by the addition of 0.1 volumes of 3 M sodium acetate and 1 volume of isopropanol. After overnight incubation at −20 °C, RNA was pelleted by centrifugation at 16,000×*g* for 30 min at 16 °C. The RNA washed once with 70% ethanol, resuspended with DEPC-water, and stored at −80 °C until use.

### Starch determination

50 mL culture was centrifuged at 500×*g* for 5 min, vortexed with 5 mL methanol, and centrifuged again at 3000×*g* for 10 min. The pellet was resuspended with methanol to a final concentration of approximately 20 × 10^6^ cells mL^−1^, amounting to 0.4–0.7 g L^−1^ starch. 500 µL of this solution was lysed with a Ribolyzer as above. Samples were cooled on ice and the methanol was evaporated under N_2_ flow. Next, 500 µL of 50 mM sodium acetate (pH 5.2) was added, the mixture was transferred to a new 2-mL tube, and the ribolyze tube was washed with 500 µL sodium acetate (this introduced a 1:2 dilution). The tube was wrapped in an aluminum foil and autoclaved at 121 °C for 15 min to solubilize starch. The tubes were shaken vigorously and centrifuged at 20,000×*g* for 2 min. Starch was enzymatically determined with a starch assay kit (Roche, Germany) using a down-scaled protocol: 10 µL supernatant was mixed with 20 µL solution 1 and incubated at 60 °C for 15 min. 200 µL of 1:2 diluted solution 2 was added and incubated at room temperature for 3 min. Absorbance was measured at 340 nm with a Tecan plate reader (infinite M200, TECAN) yielding the values for *E0*. Absorbance was again measured after incubation for 15 min at room temperature upon the addition of 2 µL solution 3, yielding the values for *E1*. The difference in absorbance (*E1* *−* *E0*) was indicative of the starch content (NADPH synthesis). The starch concentration was calculated according to a simultaneously performed calibration curve with known amounts of starch treated similarly as the cell samples (starting with the ribolyze step).

### Preparation of cDNA libraries and Illumina sequencing

For each time point, 1 µg total RNA from each of the two biological replicates (thus 2 µg in total) was mixed in equal amounts to mitigate biological variance. The resulting 12 pools were converted into cDNA libraries by poly-A fishing utilizing a TruSeq Stranded mRNA Library Prep Kit (Illumina, USA) according to the manufacturer’s instructions. RNA quality and RNA concentrations were determined using an Agilent RNA Nano 6000 kit on an Agilent 2100 Bioanalyzer (Agilent Technologies, Germany) and Trinean Xpose (Gentbrugge, Belgium), respectively. The library pool was sequenced 2× 100 nt paired-end on Illumina’s HiSeq 1500 (Illumina, USA) using rapid run mode. For each time point, approximately 33 million 2× 100 nt fragments were obtained (Additional file 1: Table ST2).

### Read mapping and transcript quantification

The quality of the reads was inspected with FastQC [133] (Additional file 1: Figure S13). Trimming was performed with Trimmomatic (version 0.32) requesting a phred33 score of 30 for the leading and trailing bases, and only trimmed reads with at least 75 bp were retained. This length was chosen because TopHat2, which was used for mapping, has been developed with 75-bp reads [48]. For read mapping, transcriptome assembly, and transcript quantification, the TopHat2-Cufflinks-protocol published in [47] was followed. As reference annotation, the refined annotation (BRAKER1-annotation, see Additional file 1: Results) was used. TopHat2 ([48], version 2.1.0) was used with default setting except for min-intron-length = 5, max-intron-length = 1418, mate-inter-dist = 53, mate-std-dev = 124, and library-type = fr-firststrand. The mapping rate was consistently approximately 90% for each time point. Cufflinks ([49, 134], version 2.2.1) was executed with default settings except for library-type = fr-firststrand, intron-overhand-tolerance = 5, min-intron-length = 5, 3-overhang-tolerance = 100, overlap-radius = 5, and max-intron-length = 1418. Parameters adjusted for Cuffquant were library-type = fr-firststrand, multi-read-correct = True, and frag-bias-correct = refined annotation file. The parameter adjusted for Cuffnorm was library-type = fr-firststrand. For quantitative analysis, evaluation was performed on the level of loci, thus not on the level of isoforms, CDS, or transcription starts sites, i.e., the FPKM values for each locus were used.

The full table of locus FPKM values, log2 fold changes relative to the pre-starvation reference time point N_0, and the respective annotations are given in Additional file 3.

### Functional annotation of the transcriptome

Annotation for the longest isoform of each locus was obtained by Blast2GO ([135], version 4.0.2) searching the “nr” database at NCBI using the algorithm “blastx-fast” with an expectation threshold of 10^−10^ reporting the 20 best blast hits. For all other settings, default values were used. For each locus, the obtained one-sentence annotation (defline), associated GO terms, and EC numbers were used for subsequent analyses. Furthermore, the best BLASTx hit for the longest isoform of each locus in the proteome of *C. reinhardtii* (Phytozome 11 [136], Creinhardtii_281_v5.5.protein.fasta) and *A. thaliana* (TAIR [137], TAIR10_pep_20101214.fasta) was obtained.

For subcellular localization prediction, the software PredAlgo [70] was used, because it was developed specifically for microalgae. As it only accepts amino acid sequences, transcripts were first translated into proteins, which was performed for only the isoform starting with a start codon (“ATG”); if multiple isoforms of a locus had a start codon as their first base triplet, only the longest of those isoforms was translated; if no isoform contained a start codon, the respective locus was excluded from analysis and the tag “NA” added. Three compartments were predicted: chloroplast, mitochondrion, and secretory pathway; if the resulting localization score was below the threshold defined by PredAlgo, the value “other” was used.

### Determination of gene sets and their GO term enrichment analysis

To dissect the transcriptional responses into those restricted to one of the three stages of alternating N availability and those shared between individual stages, an analysis of shared genes was performed. Towards this end, the mean transcript abundance in a specific stage, *A*_*stage*_, was calculated as the mean of FPKM values of the time points attributed to the respective stage. *A*_*stage*_ was related to the reference time point N_0, yielding the relative mean transcript abundance, *R*_*stage*_. Its log2-transformed variant was used to classify responsiveness of a gene in the respective stage, which was given if absolute log2-*R*_*stage*_ > 1. Consequently, for each stage, a first set with up-regulated genes (log2-*R*_*stage*_ > 1) was obtained, and a second one with down-regulated genes (log2-*R*_*stage*_ < 1), thus in total six sets.

GO terms were retrieved by Blast2GO (see above). For enrichment analysis, the GNU R (version 3.3.1) [138] package “topGO” (version2.26.0) [139] was used. To initialize the object, the function “GOdata” < −new(“topGOdata,”…) was called with default settings, except for ontology = BP. To derive enriched biological processes, the function “resultWeigthFis < −runTest(GOdata,…)” was called with default settings, except for algorithm = weight01 and statistic = fisher. Correction for multiple testing was not performed, as suggested in [139]. Significantly enriched GO terms (p < 10^−4^) were obtained by the function “GenTable(resultWeigthFis,…)” with default settings.

### Pathway reconstruction

The starch, lipid, and central carbon metabolism of *M. neglectum* was reconstructed based on pathways described for *A. thaliana* [87, 140, 141] and *C. reinhardtii* [8, 25, 32, 142, 143]. Several filters were applied to ensure a high-quality pathway reconstruction for subsequent analysis of transcriptional regulation.

For each enzymatic step, a tBLASTx search against the *M. neglectum* transcriptome was performed, yielding a list of candidate transcript loci *L_transcripts*_*enzymatic_step*_. If multiple isoforms were attributed to a transcript locus, only the longest of those isoforms was considered for subsequent validation. For validation, a reverse tBLASTx search was performed in the two model organisms *A. thaliana* and *C. reinhardtii* for each transcript in *L_transcripts*_*enzymatic_step*_, and only if the best tBLASTx hits matched the respective enzymatic step, the transcript was retained. Additionally, the predicted domain structure had to match the respective enzymatic step; domain prediction was performed by the NCBI conserved domain search web interface [64]. For instance, if the template sequence encoded an UDK (uridine kinase) superfamily domain (phosphoribulokinase, Cre12.g554800), the candidate transcripts of *M. neglectum* accordingly had to encode the UDK superfamily domain. Next, transcripts likely constituting fragment pairs were identified in a three-step approach. Towards this end, *L_transcripts*_*enzymatic_step*_ was first searched for transcripts with very similar regulation patterns (Pearson correlation >0.9). Second, only those highly correlated transcripts were retained, whose corresponding genes were classified as putatively fragmented. A gene was defined as fragmented, if it was located within 500 nt distance to the scaffold margin. The value of 500 nt was chosen, because this was the upper size of most introns of *M. neglectum* (Additional file 1: Figure S14c). Third, the predicted domain structures were inspected visually to determine whether both matched each other. This was given, for instance, when the same truncated domain was predicted, such that two transcripts had appropriately C- and N-terminal truncated domains, respectively (Additional file 1: Figure S9). If these requirements were met, the respective transcripts were considered as a fragment pair. Of those, only the leftmost fragment, i.e., containing the start codon, was retained in *L_transcripts*_*enzymatic_step*_, because this fragment encodes the putative targeting sequence for chloroplast, mitochondrion, or secretory pathway [70].

Fold changes were only calculated if the FPKM values from the reference time point N_0 and the time point of interest were both at least 1.0. Values below this threshold were highlighted by “NA” in Figs. 4, 5, and 6 and Additional file 1: Figure S10 indicating too low read support to allow for reliable transcript quantification.

### Identification of putative lipases and clustering of their transcriptional profiles

Lipase candidates were identified by searching the annotation of transcripts for either containing the keyword “lipase” or the keywords “hydrolase” and “beta” in their name, or if attributed GO terms included the term “GO:0016298” (lipase activity) or the terms “GO:0016787” and “GO:0016042” (hydrolase activity and lipid catabolic process, respectively). Hierarchical clustering with Euclidean distance and complete linkage of the log2-transformed fold change values relative to the pre-starvation time point N_0 were performed with R ([138], version 3.3.1; function “hcluster” in package “amap” [144], version 0.8.14). Euclidean distance has been recommended for log ratio data [145], and complete linkage was shown to outperform average linkage in gene expression studies [146]. The average silhouette width as a cluster quality control parameter [75] was determined for *k* = 3–7 clusters. The heatmap visualization of the dendrogram was plotted by the function “heatmap.2” of the R package “gplots” ([147], version 3.0.1), saved as SVG file, and the colors replaced according to the color scheme used in this publication.

## Additional files

**Additional file 1.** Additional Methods, Results, Tables ST1–ST3 and Figures S1–S17.

**Additional file 2.** The 100 most induced genes in the l-N stage, sorted by their log2 mean-FC (*R*_*l-N*_) values in the l-N stage in descending order; The 100 most repressed genes in the l-N stage, sorted according to their log2 mean-FC (*R*_*l-N*_) values in ascending order; The top 100 most expressed genes at the time point N_0 that were classified as not fragmented.

**Additional file 3.** The annotation and additional information for each locus; FPKM values of each locus; Log2-FC values in respect to N_0 of each locus.

**Additional file 4.** All transcripts considered for the pathway analysis of starch metabolism (Additional file 1: Figure S10b); All transcripts considered for the pathway analysis of glycerolipid metabolism (Figure 4); All transcripts considered for the pathway analysis of central carbon metabolism (Figure 6).

**Additional file 5.** The annotation and additional information of all putative transcription factors, sorted by their log2 mean-FC (*R*_*l-N*_) values in the l-N stage in ascending order.

## Abbreviations

ACCase: heteromeric acetyl-CoA carboxylase complex
ACP: acyl carrier protein
CDS: coding DNA sequence
CoA: coenzyme A
DAG: diacylglycerol
DGAT: diacylglycerol acyltransferase type 1
DGDG: digalactosyldiacylglycerol
DGTS: diacylglycerol-N,N,N-trimethylhomoserine
DGTT: diacylglycerol acyltransferase type 2
ER: endoplasmic reticulum
FA: fatty acid
FAT: acyl-ACP thioesterase
FC: fold change
FPKM: fragments per kilobase of exon per million fragments mapped
GO: gene ontology
LACS: long-chain acyl-CoA synthetase
MGDG: monogalactosyldiacylglycerol
MLDP: major lipid droplet protein
NA: not available
OPPP: oxidative pentose-phosphate pathway
PDAT: phospholipid:diacylglycerol acyltransferase
cpPDHC: plastidial pyruvate dehydrogenase complex
PEP: phosphoenolpyruvate
PGD1: plastid galactoglycerolipid degradation 1
PS: photosystem
PthCho: phosphatidylcholine
SAD: stearoyl-ACP desaturase
SE: standard error of the mean
TAG: triacylglycerol
UTR: untranslated region
