# Supplementary material for: Time-resolved transcriptome analysis and lipid pathway reconstruction of the oleaginous green microalga Monoraphidium neglectum reveal a model for triacylglycerol and lipid hyperaccumulation
Source: Biotechnol Biofuels. 2017 Aug 14;10:197. doi: 10.1186/s13068-017-0882-1 (PMC5556983; doi:10.1186/s13068-017-0882-1)
Supplement: Supplementary file 1 — Additional file 1. Additional Methods, Results, Tables ST1–ST3 and Figures S1–S17. [file 13068_2017_882_MOESM1_ESM.docx]

**Additional File 1 / Supplementary Material**

**Title**

Time-resolved transcriptome analysis and lipid pathway reconstruction of the oleaginous green microalga *Monoraphidium neglectum* reveal a model for triacylglycerol and lipid hyper-accumulation

**Authors**

Daniel Jaeger^1^, Anika Winkler^2^, Jan H. Mussgnug^1^, Jörn Kalinowski^2^, Alexander Goesmann^3^, Olaf Kruse^1^

^1^Algae Biotechnology and Bioenergy, Faculty of Biology, Center for Biotechnology (CeBiTec), Bielefeld University, 33615 Bielefeld, Germany

^2^Microbial Genomics and Biotechnology, Center for Biotechnology (CeBiTec), Bielefeld University, 33615 Bielefeld, Germany

^3^Bioinformatics and Systems Biology, Justus-Liebig-Universität, 35392 Gießen, Germany

**Additional File 1: Methods**

**Re-annotation of the genome based on BRAKER1 by incorporating mRNA-seq data**

The mRNA-seq reads from nine of the twelve time points were aligned to the genome of *M. neglectum* [[1](#_ENREF_1)] using TopHat2 (version 2.1.0) with default parameters except for: min-intron-length = 5, max-intron-len = 1418, mate-inner-dist = 53, mate-std-dev = 124. The nine time points were N_0, N_2, N_4, N_24, N_48, N_96, R_2, R_4 and R_14; the full set of time points was not chosen due to memory limitations. The resulting BAM file was used by BRAKER1 [[2](#_ENREF_2)] to obtain intron hints, which was executed with default settings (version 1.6 from May 11th, 2015). BRAKER1 internally calls AUGUSTUS (version 3.2.1), GeneMark-ET (version 4.21) and bamtools (version 2.4.0) [[3-5](#_ENREF_3)]. To ensure that the exclusion of the remaining three time points did not result in a significant loss of transcript information, the read data was re-mapped to the transcriptome encoded by the BRAKER1 annotation. Towards this end, the GFF file obtained by BRAKER1 was converted to a transcriptome FASTA file (BRAKER1-transcriptome) using the gffread utility from Cufflinks [[6](#_ENREF_6)]. Read pairs that were retained after trimming were re-aligned individually for each time point to the BRAKER1-transcriptome using Bowtie2 (version 2.3.1) with default settings [[7](#_ENREF_7)], and the overall alignment rates were recorded (Table ST2).

**Comparison of the previous annotation with the refined version obtained in this study by BRAKER1**

The structural annotation stored in the GFF file obtained by BRAKER1 was compared to the structural annotation stored in the GBF genbank file obtained in a previous study [[1](#_ENREF_1)] with the following algorithm (“braker_gene” refers to a gene from the refined annotation, whereas “gbf_gene” refers to a gene from the previous annotation of [[1](#_ENREF_1)]):

| For each gbf_gene  go through each braker_gene on the same scaffold  is there some intersection between the genomic region of the gbf_gene and braker_gene?  (genomic region = start of gene to end of gene, anywhere there)  yes -> mark as not unique; determine shared exons, defined as exons that share both  start and end coordinates between the two annotations*:  overlap = 0.0 -> 0 shared exons = only genomic coordinates equal, no shared exon  overlap = 0.01-0.99 -> ≥1 shared exons = partly refined annotation  overlap = 1.0 -> all exons shared = annotation was already perfect  no -> mark as unique_ gbf_gene  *to calculate the relative proportion of shared exons, the number of shared exons was divided by the number of exons of the longer gene variant from both annotations. |
| --- |

**Comparative CDS composition analysis**

The genome annotation GFF files for *Chlamydomonas reinhardtii* [[8](#_ENREF_8)] were downloaded from http://genome.jgi.doe.gov/pages/dynamicOrganismDownload.jsf?organism=PhytozomeV9 (file = Creinhardtii_236_gene.gff3), for *Nannochloropsis gaditana* [[9](#_ENREF_9)] from http://nannochloropsis.genomeprojectsolutions-databases.com/ (file = Nanno_annotation_v1.1.gff) and for *Phaeodactylum tricornutum* [[10](#_ENREF_10)] from http://genome.jgi.doe.gov/Phatr2/Phatr2.home.html (file = Phatr2_geneModels_FilteredModels2.gff), while the one for *M. neglectum* was obtained in this study by BRAKER1 (see above).

UTRs were only consistently annotated for *C. reinhardtii*. To enable unbiased comparison between all four species, only those exons coding for the translated sequence (coding sequence exons, CDS-exons) were considered. CDS-exons were identified by the tag “CDS” in the third column of the GFF file, which contains the feature’s type . CDS-exons were attributed to their gene locus according to the ninth column, which contains the feature’s attributes. This column contained a unique locus identifier named “pacid”, “ID” and “name” for *C. reinhardtii*, *N. gaditana* and *P. tricornutum*, respectively. The coordinates of the CDS-exons were parsed, yielding an exon coordinate list for each gene. This list was sorted in ascending order. Intron coordinates were subsequently obtained by adding one to the end position of a previous exon, and subtracting one from the start position of a following exon. The length of the CDS of a gene was given by the sum of lengths of all its CDS-exons.

For a minor fraction of the genes (< 10 %) from the *C. reinhardtii* GFF file, alternatively spliced transcripts were annotated. In order to avoid bias in that those transcripts contribute to the analysis several-fold according to their number of alternatively spliced isoforms, only the longest isoform was retained, tagged as “longest=1” in the attribute column.

For *M. neglectum*, putatively fragmented genes were discarded to avoid a bias towards smaller, truncated exon or intron sizes. These were identified by the absence of start and / or stop codon, which were explicitly given in the GFF file obtained by BRAKER1.

The data was visualized with GNU R ([[11](#_ENREF_11)], version 2.14.1), and the magnitude of difference in CDS compositions quantified by effect sizes analysis according to [[12](#_ENREF_12)].

**Identification of putative dominant isoform switching events**

To filter gene loci which showed a switching of the dominant isoform under –N conditions compared to the N_0 time point, the dominant isoform at time point N_0 was compared to the dominant isoform from each time point of –N conditions. Candidates were retained if at least three of the eight time points of –N conditions had a different dominant isoform than the one from the N_0 time point. To retain only candidates with a clear switching pattern, candidates were filtered for cases where the relative abundance of the dominant isoform from the N_0 time point dropped by at least 50 % at the respective time point of –N. To further retain only candidates with a high confidence in respect to correct assembly, a filter based on FPKM values was applied. According to this, only candidates were retained for which the FPKM value of the dominant isoform at N_0 was at least 7, and for which the FPKM values of the two most abundant isoforms at a specific –N time point was at least 7 and 3.5, respectively. To exclude cases in which uncertainty of annotation was the reason for dominant isoform switching, the length of the dominant isoform at N_0 had to differ by at least 30 nucleotides (i.e. 10 amino acids) from the dominant isoform at the respective time point of –N. Finally, it was requested that all isoforms attributed to a single locus shared at least one exon.

**Construction of a transcriptome database for *M. neglectum* incorporating also transcript information of other microalgae**

The transcriptome data from *M. neglectum* has been integrated into a MYSQL database, which can be queried via a web interface based on CGI scripts. It is available at https://tdbmn.CeBiTec.Uni-Bielefeld.DE/. The CGI scripts require python (version 2.7.3), R (version 2.14.1) and rpy2 (version 2.2.5) for data management and visualization. The structural annotation of loci, isoforms and transcription start sites was obtained from the files provided by Cufflinks (version 2.2.1) [[13](#_ENREF_13)], from which also the FPKM values for transcript loci and isoforms were retrieved. The functional annotation of the transcripts (description, GO terms, EC numbers) was obtained from BLAST2GO (version 4.0.2) [[14](#_ENREF_14)]; see also methods of the main article. Putative domain structures were predicted by the NCBI conserved domain search (version 3.16) using the “Batch CD search” option [[15](#_ENREF_15)]. For this, protein sequences were required, which were obtained from the GFF file storing the BRAKER1 [[2](#_ENREF_2)] reference annotation obtained (see above), because this file also contained the translated amino acid sequences of the predicted genes. Localization prediction was performed by PredAlgo [[16](#_ENREF_16)] as described in the main article.

Published mRNA-seq datasets of other microalgae also subjected to –N treatment were additionally integrated into the database [[17-21](#_ENREF_17)]. The respective transcript data was obtained from the supplementary materials of [[17-21](#_ENREF_17)]. Therefore, the database contains transcript data from –N conditions for *M. neglectum*, *Chlamydomonas reinhardtii*, *Nannochloropsis oceanica*, *Phaeodactylum tricornutum*, *Neochloris oleoabundans* and *Botryosphaerella sudeticus*. To enable BLAST search, transcript nucleotide sequence data was stored for *M. neglectum*, *C. reinhardtii* [[8](#_ENREF_8)], *N.* *oceanica* [[22](#_ENREF_22)] and *P. tricornutum* [[10](#_ENREF_10)]. The cultivation conditions were mixotrophic for *C. reinhardtii* (TAP –N medium), and autotrophic for the other microalgae. Enriched levels of CO_2_ were provided to *M. neglectum* (3 %), *B. sudeticus* (2 %) and *N. oceanica* (1.5 %), while ambient levels of CO_2_ were used for the cultivation of *P. tricornutum* and *N. oleoabundans* [[17-21](#_ENREF_17)]. Time-course experiments were performed for *M. neglectum* (0 – 96 h of –N), *C. reinhardtii* (0 – 48 h of –N) and *N. oceanica* (3 – 48 h of –N), while single time point analysis were conducted for *B. sudeticus* (72 h of –N), *N. oleoabundans* (11 d of –N) and *P. tricornutum* (48 h of –N) [[17-21](#_ENREF_17)].

**Additional File 1: Results and Discussion**

**Incorporating transcript information for genome annotation refines gene structures and improves prediction of exon boundaries for *M. neglectum***

In order to validate the previously *ab initio* predicted gene models of *M. neglectum* [[1](#_ENREF_1)], the unprocessed mRNA-seq data was incorporated into the process of gene prediction, for which the software BRAKER1 was used [[2](#_ENREF_2)]. BRAKER1 was shown to achieve high gene prediction accuracies when mRNA-seq data was used as the sole source of evidence [[2](#_ENREF_2)]. Read data from nine of the twelve time points were pooled, which were from +N, -N and N resupply conditions. They represented approximately 300 million fragments (600 million reads) of ~100 bp length. Assuming a transcriptome size of 32 mbp based on the length of the longest isoform attributed to each transcript locus, this pool of reads represented a theoretical transcriptome coverage of more than 1800-fold. It has been recommended for experiments, whose purpose is the discovery of novel transcripted elements, that a minimum of 100 – 200 million 2x 76 bp reads should be acquired [[23](#_ENREF_23)]. Accordingly, more reads (~300 million) were used for the annotation refinement purpose in this study. As furthermore this recommendation refers to cells from mammalian tissues, whose transcriptomes are likely more complex than that of the unicellular eukaryote *M. neglectum*, it was assumed that practically all transcripts expressed under the setup conditions should be represented in this dataset.

To nevertheless ensure that the three excluded time points (N_8, N_56, R_8) did not contain surplus transcript information, the read pairs from all twelve time points were individually aligned to the transcriptome encoded by the BRAKER1-annotation. The overall alignment rates revealed that these three time points had similar alignment rates as adjacent time points (Table ST2). Therefore, if these three time points were included into the pool of reads used as input for BRAKER1, they would likely not increase sensitivity further; accordingly, their exclusion from the read pool seemed reasonable.

Prediction by BRAKER1 resulted in 19,667 nuclear genes, which were ~18 % more compared to the previous annotation (16,735 nuclear genes) from [[1](#_ENREF_1)]. Less than 1 % (n=157) of the previously predicted genes were not contained in the refined annotation, highlighting the low rate of false gene prediction by the previous annotation approach [[1](#_ENREF_1)]. Approximately 3 % (n=558) of the previously predicted genes were found to likely be gene fusions, indicated by mapping of a single locus to multiple loci in the refined annotation. Another 10 % (n=1,640) of the previously predicted genes were putative gene fragments, indicated by mapping of multiple loci to a single locus in the refined annotation. The remaining 85 % (n=14,442) mapped uniquely and were used to assess the prediction accuracy of the previous annotation from [[1](#_ENREF_1)]. A perfect match of gene structures was found for 21 % (n=3,070) of the previously predicted genes, which is slightly lower than the reported rate of 40 % for the human sag178 dataset in [[3](#_ENREF_3)]. This could be due to the larger dataset for *M. neglectum*, or alternatively possibly due to the higher GC content of this microalga. A partial match for the previously predicted genes, given if only a subset of exons mapped perfectly, was found for 65 % (n=9,400). Finally, no exon match despite overlap of genomic coordinates was found for 14 % (n=1,972), indicating that exon structures of those genes necessitated complete refinement. When the sets of all exons without the gene context (i.e. ignoring their ordering) were compared, it became apparent that 75 % of the previously annotated exons matched perfectly to a counterpart in the refined annotation. This is in good accordance to [[3](#_ENREF_3)] in which a rate of approximately 80 % correctly predicted exons was reported. Conversely, 57 % of the exons from the refined annotation were also found in the previous annotation, highlighting that more than half of the coding information was already perfectly recovered by the *ab initio* approach from [[1](#_ENREF_1)].

***M. neglectum* has an intron-rich composition of coding DNA sequences**

The natural gene architecture of the host organism is an important feature that can affect transgene expression levels. For example, it was shown for the chlorophyceae *Chlamydomonas reinhardtii* that incorporation of introns into a recombinant gene of interest facilitates its expression in genetic engineering approaches [[24-28](#_ENREF_24)], likely being due to mimicking the native intron-rich [[29](#_ENREF_29)] gene structure of *C. reinhardtii*. In order to investigate if a similar scenario might apply for the chlorophyceae *M. neglectum*, we used the improved annotation obtained in this study to analyze the composition of coding DNA sequences (CDS) of *M. neglectum* and to compare it with the three other microalgae *C. reinhardtii*, *Nannochloropsis gaditana* and *Phaeodactylum tricornutum*.

The genome of *M. neglectum* currently is available as 6,739 scaffolds [[1](#_ENREF_1)]. This translates into more than 13,000 scaffold margins. Each CDS model that is located at a scaffold margin has a certain risk of fragmentation, in that the first part of the corresponding protein might be encoded at the end of a first scaffold, whereas the second part might be encoded at the start of a second scaffold. In such a case, the exon directly adjacent to the scaffold margin might be cropped, which could potentially introduce a bias towards smaller feature sizes in *M. neglectum*. Therefore, only putative full-length CDS models were considered for the quantification of CDS features. Those were identified by the presence of both a start and stop codon, which was given for 63 % of the CDS models (n=12,435). We note that this definition is different from the more conservative definition of truncation used for the pathway analysis (within 500 bp of a scaffold margin); the latter was chosen because start and stop codons were not annotated for transcripts assembled by the Cufflinks software.

In order to evaluate the magnitude of observed difference in CDS features, we applied effect size analysis based on Cohen’s d [[12](#_ENREF_12)] and not P value-based evaluation. This was due to the large sample size of the datasets, such as 73,486 exons in *M. neglectum*. Otherwise, differences with negligible absolute distances might achieve a very high statistical significance, because the sample size is taken into account for classical tests such as for the Student’s T-Test. In contrast, effect size is independent of sample size and indicates the magnitude of the difference between groups [[30](#_ENREF_30)].

First, the median intron content of CDS of *M. neglectum* was found to be more similar to *C. reinhardtii* (4 and 6 introns per CDS, respectively), compared to the distantly related eustigmatophyceae *N. gaditana* and the diatom *P. tricornutum* (1 and 0 introns per CDS, respectively) (Figure S14a). This was supported by values for Cohen’s d (Table ST3). In order to analyze whether the amount of introns per CDS had an effect on transcript abundances in *M. neglectum*, both factors were correlated (Figure S14b). Although no dependence was found, the majority of the most highly expressed genes contained between two and six introns (Figure S14b, grey dots). This indicated that introns were also not an impediment for high gene expression.

In order to investigate whether the increased amount of introns in *C. reinhardtii* and *M. neglectum* compared to *N. gaditana* and *P. tricornutum* translated into shorter exons, we next determined the distances between individual exons and introns. As a result, the median intron sizes were very similar between the two chlorophyceae *M. neglectum* and *C. reinhardtii* (243 bp and 227 bp, respectively), as also were the median exon sizes (125 bp and 132 bp, respectively), but they were generally shorter compared to *N. gaditana* and *P. tricornutum* (Figure S14c, d; Table ST3). In regard that the median exon sizes between *M. neglectum* and *C. reinhardtii* were similar, but the median intron content being reduced by 40 %, it was hypothesized that the median CDS length was shorter in *M. neglectum*. Indeed, its median CDS length was more similar to *N. gaditana* and *P. tricornutum*, which was supported by values for Cohen’s d (Figure S14e; Table ST3). A possible explanation for the shorter CDS length in *M. neglectum* could be the hypothesized smaller regulatory repertoire of *M. neglectum* compared to *C. reinhardtii* [[1](#_ENREF_1)]. In this scenario, additional regulatory domains might be less frequent in *M. neglectum*, thereby reducing CDS length. Alternatively, the filtering procedure, during which full-length CDS models were requested, might potentially have resulted in an underrepresentation of longer CDS, because those intrinsically have a higher probability of being classified as truncated. To confirm whether CDS lengths are generally shorter in *M. neglectum* compared to *C. reinhardtii*, a chromosome-scale genome assembly of *M. neglectum* would be required.

Interestingly, correlating CDS length and transcript abundance in *M. neglectum* showed a slightly decreasing trend towards lower transcript abundance with increasing CDS length (Figure S14f). The CDS of the majority of the most highly expressed genes was shorter than 1000 bp (Figure S14f, grey dots), although exceptions were found, such as the 60s ribosomal protein l4 (XLOC_000605) with a CDS length of 3,321 bp. This general trend is in accordance with the described constraint of gene expression on its length [[31](#_ENREF_31)], and was also observed in mice [[32](#_ENREF_32)] and a simulated study [[33](#_ENREF_33)].

In summary, the comparative CDS composition analysis suggests that similar genetic engineering strategies as applied for *C. reinhardtii* [[34](#_ENREF_34)] might also be applicable for *M. neglectum*, such as identification of cis-acting genetic elements (e.g. regulatory introns [[25](#_ENREF_25)]) promoting foreign gene expression and their regular distribution into the recombinant transgene of interest. This, however, remains to be confirmed in systematic genetic transformation studies.

**Evidence for alternative splicing and dominant isoform switching under –N conditions in *M. neglectum***

Using Cufflinks for transcriptome assembly as part of the Tuxedo pipeline [[6](#_ENREF_6)], 20,751 loci and 35,146 isoforms were obtained. The higher number of isoforms was due to the presence of untranslated regions (UTRs) in the transcripts assembled by Cufflinks, whereas only the CDS-version of the respective gene was predicted by BRAKER1. As a result, 85 % of all transcript loci had either a single or two isoforms attributed, in the latter case the provided “UTR-free CDS isoform” obtained by BRAKER1 and the fully annotated version including UTRs obtained by Cufflinks.

For the remaining 15 %, it was checked whether a switch in dominant isoforms during the time course of –N treatment could be detected. Switching of dominant isoforms was shown for mouse myoblasts during differentiation [[35](#_ENREF_35)]. For this purpose, the dominant isoform at time point N_0 was determined, and compared to the dominant isoform from each time point of –N conditions. Applying several filter steps, ten candidate loci were obtained. Of those, three were likely false positives, because the pattern of relative isoform abundances at N_0 was unique compared to all other time points (data not shown). For the remaining seven loci, a switch in the dominant isoform during –N conditions could be visually confirmed, and this switch was reversed upon N resupply. Three different effects were observed: (i) unaltered domain structure but different transcript lengths (Figure S3), (ii) altered domain structures by presence or absence of additional domains (Figure S4), and (iii) completely different proteins (Figure S5). In all cases, the switch in the dominant isoform was due to an alternative transcription start site, and not due to alternative splicing (Figures S3-S5). Nonetheless, evidence for alternative splicing was found (Figure S6); however, a more detailed analysis is required to address the extent of alternative splicing in *M. neglectum*, as has been performed for *C. reinhardtii* [[29](#_ENREF_29)].

**Development of a transcriptome database for *M. neglectum***

For convenient web page-based access of the transcript data of *M. neglectum*, a database was constructed. The purpose was to visualize all information in regard to gene annotation and gene expression of *M. neglectum*. Furthermore, published mRNA-seq datasets from other microalgae were included. This was to enable the comparison of transcriptional regulation of individual genes between different microalgae, and to thus determine putatively conserved transcript responses under –N conditions.

In regard to the first purpose, two different levels of gene annotation were integrated. These were structural annotation of the gene locus, and functional annotation of the gene product. The former refers to the visualization of all assembled isoforms and predicted transcription start sites attributed to a transcript locus. Isoforms and transcription start sites have the prefixes “TCONS_...” and “TSS_”, respectively, while transcript loci have the prefix “XLOC_...”. Furthermore included was the respective gene name in the BRAKER1 reference annotation; it has the prefix “g…”. To provide information about the functional annotation of the corresponding gene product, a tabular summary and a visualization of the predicted domain structure are displayed. The tabular summary contains transcript description, predicted subcellular localization, as well as attributed GO terms and EC numbers. It additionally contains structural information such as the transcript locus length and if the locus has been tagged as putatively truncated (located within 500 bp of the scaffold margin).

For visualization of gene expression, the expression on the level of isoforms (isoform-FPKM) and on the level of transcript loci (locus-FPKM) has been integrated. While the locus-FPKM values were the basis for this study, the isoform-FPKM values were additionally integrated, because the latter can be used to retrieve transcript information of gene fusions. As an example, we were interested in the expression of phosphoglycerate kinase (PGK) genes of *M. neglectum*. The locus XLOC_012520 is annotated as PGK; however, it most likely represents a fusion of two individual genes (Figure S15a). This was indicated by the respective name from the BRAKER1-reference annotation, which in this case was not a single entry such as “g12298”, but rather a comma-separated list of several names, i.e. “g12298,g12299”. Analysis of the individual isoform abundances revealed that the PGK transcript (g12298, TCONS_00022968) was markedly more abundant than the transcript of the second gene (g12299, TCONS_00022969) (Figure S15a). Therefore, the higher-level locus-FPKM values, which are the sum of the isoform-FPKM values [[6](#_ENREF_6)], are a reliable approximation of the transcript abundances of the putative PGK gene, despite transcript fusion during transcriptome assembly. We also noted the reverse, i.e. the locus-FPKM values were not accurately representing the transcript abundances of the gene of interest. This applied for instance to a second PGK transcript locus (XLOC_004946), likely also representing a fusion of two genes (g4930,g4931) (Figure S15b). In this case, the transcript levels of the second gene (g4931, TCONS_00009452, a putative DNA binding protein) were markedly higher than these of the PGK gene (g4930, TCONS_00009451) (Figure S15b). Therefore, the higher-level locus-FPKM values did not accurately reflect the transcript levels of this second PGK gene, and hence this locus was ignored in further analysis. To visualize expression on the level of transcript loci, a plot of the absolute and log10-transformed locus-FPKM values is generated, as well as of the absolute and log2-fold change values relative to the reference time point N_0. Furthermore, an expression pictogram is drawn, which was used to visualize transcriptional regulation of the glycerolipid, central carbon and starch metabolism in Figures 4 and 6 and in Figure S10b, respectively. It is also possible to search for loci whose transcript patterns are most strongly correlated with the present locus. This allows identification of similarly regulated genes, thus possibly being part of the same transcriptional module, or being different components of the same cellular pathway.

Two search strategies are implemented to query the database. The first requires *a priori* knowledge of transcript locus identifier (XLOC_...). It might for instance be retrieved from Additional files 2 – 5. This search function displays all gene annotation and gene expression information of the queried locus. Optionally, a list of locus identifiers might be submitted, and as a result, plots are created that display the expression of all queried loci; detailed information for each individual locus can be subsequently retrieved. The purpose of the second search strategy is to identify transcripts sharing a similar annotation, and thus to identify all genes with a specific function. Towards this end, two approaches are implemented. The first approach is BLAST search (tBLASTx, tBLASTn). The second approach is to search by name or keyword (e.g. phosphoglycerate kinase), by GO term (GO:0004618, phosphoglycerate kinase activity), or by EC number (2.7.2.3, phosphoglycerate kinase).

Importantly, the second search strategy, i.e. the identification of transcripts with the same predicted function, additionally integrates mRNA-seq datasets from other microalgal species, which were also subjected to –N conditions. BLAST search can thus not only be performed against the transcriptome of *M. neglectum*, but also of *Chlamydomonas reinhardtii* [[8](#_ENREF_8)], *Nannochloropsis oceanica* [[22](#_ENREF_22)] and *Phaeodactylum tricornutum* [[10](#_ENREF_10)]. The other search mode (name/keyword, GO term, EC number) is available for *M. neglectum*, *C. reinhardtii*, *N. oceanica*, *P. tricornutum*, *Neochloris oleoabundans* and *Botryosphaerella sudeticus*.. BLAST search of the latter two species was not implemented, because transcript sequence data was not deposited as supplementary materials in those studies. Note that the dataset for *C. reinhardtii* contains transcript information for different strains, which are a wild-type like strain (CC4532, WT), a cell wall-less strain (CC4349, *cw15*), and a cell wall-less and starch-less strain (CC4348, *sta6*) [[17](#_ENREF_17)].

**Identification of a conserved transcript response of microalgae to nitrogen starvation**

Two enzymatic steps of the central carbon metabolism of *M. neglectum* were subjected to strong transcriptional regulation in the l-N stage. These were the oxidation of glucose 6-phosphate to 6-phospho-D-glucono-1,5-lactone, and the phosphorylation of 3-phosphoglycerate to 1,3-bisphosphoglycerate. The reactions are catalyzed by glucose 6-phosphate dehydrogenase (G6PDH) and PGK, respectively. While G6PDH was strongly up-regulated, PGK was strongly down-regulated in the l-N stage in *M. neglectum* (Fig. 6). This likely results in a re-routing of glucose 6-phosphate through the OPPP (oxidative pentose phosphate pathway) to 3-phosphoglycerate via carboxylation of ribulose-1,5-bisphosphate, catalyzed by RuBisCo, finally towards pyruvate production for subsequent acetyl-CoA generation (see discussion of the main article). The two enzymes G6PDH and PGK might exert key functions in this process, because the up-regulation of G6PDH might “open the gate” for the OPPP route, while the down-regulation of PGK might “close the gate” for the Calvin cycle. The down-regulation of PGK likely results in a reduced rate of 1,3-bisphosphoglycerate production (Calvin cycle direction), and as a result, 3-phosphoglycerate can be increasingly isomerized to 2-phosphoglycerate (glycolytic direction).

To investigate whether this transcript response could also be observed in other microalgae under –N conditions, we queried our database to retrieve transcript information for the G6PDH and PGK genes. We used the BLAST search approach (tBLASTx) to identify putative G6PDH and PGK transcripts in *C. reinhardtii*, *N. oceanica* and *P. tricornutum*. For *N. oleoabundans* and *B. sudeticus*, we applied EC number search with the search terms 1.1.1.49 (glucose-6-phosphate dehydrogenase) and 2.7.2.3 (phosphoglycerate kinase), respectively. For all species except *B. sudeticus*, we found several putative G6PDH and PGK transcripts; for *B. sudeticus*, one additional G6PDH and one additional PGK transcript could be identified by GO term search (GO:0004345 and GO:0004618, respectively). For each species, we retained only the transcript with the highest expression at the reference time point for further analysis.

We found that PGK was down-regulated in all other microalgae under –N conditions (Figure S16). In contrast, G6PDH was up-regulated in the chlorophyceae *M. neglectum* (selenastraceae), *C. reinhardtii* (chlamydomonadaceae) and *N. oleoabundans* (neochloridaceae), but neither in the chlorophyceae *B. sudeticus* (neochloridaceae) nor the diatom *P. tricornutum* (phaeodactylaceae) (Figure S16). Transcript data for the eustigmatophyceae *N. oceanica* (monodopsidaceae) were not available (Figure S16). This was because only genes with significant differentially expression were included in the supplementary material of [[18](#_ENREF_18)]. The absence of significant differential expression, however, indicates that G6PDH is likely not up-regulated in *N. oceanica* under –N conditions. To conclude, the down-regulation of PGK under –N conditions might thus be central to re-direct the flow of triose phosphates towards pyruvate generation by glycolytic reactions in microalgae. The transcriptional induction of the OPPP, however, might either be not conserved to the same extent, or alternatively be due to different culture conditions in those studies, such as light intensity and CO_2_ availability.

**Transcripts implicated in nitrogen assimilation belong to the strongly induced genes of *M. neglectum*, while photosynthetic genes were strongly repressed in the l-N stage**

In order to identify the pathways that were subjected to the most pronounced transcriptional changes in the l-N stage, transcripts were sorted according to their mean-FC of this stage (*R_stage_* values, see methods in the main article). The set of the most strongly up-regulated genes encoded almost the complete set of N assimilation proteins, including a putative nitrate reductase, nitrite reductase, and glutamate synthetase (Additional file 3). Additionally, putative N transporters for ammonium, urea, amino acids and peptides, as well as putative acetamidase, formamidase and urea carboxylase transcripts were among the transcripts most strongly increased in the l-N stage (Additional file 3). This suggested that *M. neglectum* might be able to use external organic N sources, which was confirmed for urea (Figure S8).

The set of the strongest down-regulated genes consisted mostly of light harvesting protein homologs, subunits of photosystem I and components of chlorophyll biosynthesis, as well as phosphoglycerate kinase and one RuBisCo subunit (Additional file 3). This indicated a coordinated down-regulation of photosynthesis and carbon fixation as major responses to prolonged nitrogen starvation as has been reported before for other microalgae [[17](#_ENREF_17), [21](#_ENREF_21), [36](#_ENREF_36), [37](#_ENREF_37)].

**Transcriptional regulation of putative transcription factors of *M. neglectum* in the l-N stage**

The Plant Transcription Factor Database [[38](#_ENREF_38)] was used to predict 193 putative transcription factors in the transcriptome of *M. neglectum*. 40 putative transcription factors were up-regulated and another 40 down-regulated in the l-N stage (Additional file 4). Interestingly, the family bZIP was more abundant in the set of up-regulated transcription factor genes, whereas the family GATA was more abundant among those down-regulated (Figure S17). As expected, the putative transcription factor NIT2 likely implicated in N metabolism [[39](#_ENREF_39)] was included in the set of up-regulated transcription factors (putative fragment pair XLOC_000425 and XLOC_017762, Additional file 4). The most strongly up-regulated putative transcription factor belongs to the MYB family, and was also part of the set of the most strongly up-regulated genes in the l-N stage (XLOC_013389, Additional file 3). Its two closest hits in *C. reinhardtii* (Cre03.g197100 [[40](#_ENREF_40)]; Cre01.g034350 [[41](#_ENREF_41)]) suggested that it might be implicated in core metabolic regulation. Interestingly, the candidate gene for the transcription factor NRR1 (Nitrogen Responsive Regulator) in *M. neglectum* also appeared in the list of up-regulated transcription factors, although induction was moderate (XLOC_008254, Additional file 4). In *C. reinhardtii*, NRR1 was mapped to lipid accumulation, and *knock-out* reduced lipid content by approximately 50 % [[42](#_ENREF_42)]. This might indicate that part of the transcriptional regulation of lipid accumulation could potentially be conserved between *C. reinhardtii* and *M. neglectum*. The most strongly down-regulated putative transcript factor (XLOC_005581, Additional file 4) belongs to the GATA family, and is an interesting candidate for down-regulation under +N conditions to mimic transcriptional regulation of –N conditions (Tab. 1).

**Development of a modified heat map representation to analyze the transcriptional regulation in the two stages of nitrogen starvation and the stage of nitrogen resupply**

For comprehensive analysis of transcriptional regulation, it is important to consider both, the absolute transcript levels at the reference time point as well as the extent of up- or down-regulation during cultivation changes (x-fold changes or log2-FC). As an example, a >fourfold increase (from 11 to 49) was observed for the FPKM values of the putative DGTT enzyme transcript XLOC_007983 after 48h of –N conditions (Figure 4, second DGTT transcript from top; Additional file 2). In contrast, the FPKM value of the putative DGTT enzyme transcript XLOC_016096 only increased by a factor of three (from 36 to 111) in the same time frame (Figure 4, seventh DGTT transcript from top; Additional file 2). Although the x-fold change of the FPKM values for XLOC_016096 is lower than the x-fold change of XLOC_007983, the absolute transcript abundance of XLOC_016096 in the l-N stage is far higher (>two-fold) than the abundance of the XLOC_007983 transcripts. Therefore, despite the lower x-fold change value, XLOC_016096 might be more relevant than XLOC_007983 for neutral lipid accumulation, especially if both enzymes have similar catalytic properties.

We therefore decided to visualize both aspects of transcript abundances in a modified heat map representation (Figure S10a). This map contains the extent of differential transcriptional regulation at the individual time points of the three stages of N availability, as well as the absolute transcript abundance at the reference time point (N_0), represented by one of five abundance categories (category I – V). The abundance categories were defined according to percentiles, of which the 50 %, 75 %, 90 % and 99 % percentiles of the N_0 time point were chosen to delineate the five abundance categories, representing FPKM values of 11, 24, 58 and 866, respectively (Figure 3a). Accordingly, transcripts that had FPKM values < 11 were from genes with below-median expression (category I), those with FPKM values < 24 showed moderate expression (category II), those with FPKM values < 58 were considered to be highly expressed (category III), those with FPKM values < 866 defined as very highly expressed (category IV), and finally high-expression outliers with FPKM values > 866 (category V) (see figure keys of Figures 4 – 6). As an additional information in the modified heat map representation, the putative protein localization as predicted by PredAlgo software [[16](#_ENREF_16)] is indicated.

**Reconstruction and prediction of compartmentalization of the central carbon metabolism of *M. neglectum***

As the central carbon metabolism determines the availability of acetyl-CoA for FA synthesis, we reconstructed the central carbon metabolism of *M. neglectum*. In plants, glycolysis and the oxidative pentose-phosphate pathway (OPPP) are duplicated between the chloroplast and the cytosol [87]. This is different in *C. reinhardtii*, for which the OPPP is reported to be entirely plastidial, whereas glycolysis is highly compartmentalized [32]. The initial steps of glycolysis take place in the chloroplast, while the later steps from 3-phosphoglycerate to pyruvate are located in the cytosol [32]. Based on localization prediction by PredAlgo [77], a similar compartmentalization is proposed for *M. neglectum*, including the metabolic constrains reported for *C. reinhardtii* [32]. This was because the oxidative branch of the OPPP was predicted to be exclusively chloroplast localized in *M. neglectum* (Figure 6, G6P-DH, PGL, 6PG-DH). In addition, at least one protein with putative chloroplast localization was assigned to each step in glycolysis from fructose-6-phosphate to 3-phosphoglycerate (Figure 6, PFK, FBA, TPI, GAPDH, PGK). One important difference between *M. neglectum* and *C. reinhardtii* in regard to compartmentalization of glycolysis is that *M. neglectum* can utilize glucose as a sole carbon source (Additional file 1: Figure S11), whereas *C. reinhardtii* cannot [88]. It is therefore tempting to speculate that *M. neglectum* can perform the initial steps of glycolysis from glucose to the triose phosphates (dihydroxyacetone phosphate and glyceraldehyde 3-phosphate) additionally in the cytosol and further localization studies will be required to clarify this issue. According to the compartmentalization of glycolysis in *C. reinhardtii*, the last three enzymatic steps from 3-phosphoglycerate to pyruvate should take place in the cytosol [32]. This could be confirmed for both enolase candidates of *M. neglectum*, because both were predicted to be cytosolic proteins (Figure 6, ENO). However, one of eight phosphoglycerate mutase candidates and one of ten pyruvate kinase candidates had a weak chloroplast targeting sequence (Figure 6, PGM and PK, respectively). Nevertheless, it is postulated that the bulk flow of 3-phosphoglycerate to pyruvate takes place in the cytosol in *M. neglectum*, because the transcript levels of both putatively chloroplast localized enzymes were modest compared to the other cytosolic candidates for these two enzymatic steps (Figure 6, PGM and PK, category III and I, respectively).

**Transcriptional regulation of starch metabolism in *M. neglectum***

Synthesis of starch and lipids under autotrophic conditions relies both on fixed carbon, therefore it is feasible to suggest that starch and lipid accumulation are at least two inter-linked, if not competing, processes [[43-46](#_ENREF_43)]. The extent of this inter-linking, is not clear, since starch-less mutants had FA contents similar to the controls on a cellular level (as pg cell^-1^, as opposed to the relative fraction of the biomass, expressed as %) [[47-49](#_ENREF_47)], at least until 48 hours of mixotrophic –N conditions [[50](#_ENREF_50)]. Additionally, no correlation between starch and TAG content was found between different laboratory strains of *C. reinhardtii* [[47](#_ENREF_47)]. Furthermore, a *C. reinhardtii* mutant defective in a plant-specific DYRK kinase exhibited increased levels of both, starch and lipids under –N [[51](#_ENREF_51)]. Finally, the starch metabolism is central for the performance of the photosynthetic host [[52](#_ENREF_52)]. This is because blocking starch synthesis in *C. reinhardtii* resulted in sensitivity to photoinhibition and decreased carbon fixation rates due to reduced NADPH re-oxidation [[52](#_ENREF_52)]. Furthermore, carbon precursors for lipids (malonyl-CoA), amino acids and sugars accumulated in the *sta6* strain under exponential growth (+N) conditions, which were interestingly not channeled into the respective pathways [[52](#_ENREF_52)]. Therefore, the putative starch metabolism of *M. neglectum* was reconstructed from genomic and transcriptomic data.

As a result, candidates for all enzymatic steps of the putative starch metabolism were identified, although localization prediction did not match the putative plastidial localization for most proteins (Figure S10b).

Massive net starch synthesis was observed in the e-N stage (Fig. 1b), yet a transcriptionally only transient induction of no more than 2.5-fold of the small catalytic subunit of the heteromeric ADP-glucose pyrophosphorylase (AGPP) complex was found after two hours of –N treatment (small AGPP in Figure S10b). A transient increase in transcript abundance at this time point was noted for the majority of transcripts putatively implicated in starch metabolism (Figure S10b). The transient and furthermore gentle up-regulation of the small AGPP subunit was surprising, because it was supposed to control the flux of carbon into starch [[53](#_ENREF_53)]. An explanation could be that its pre-starvation levels were already capable of massive starch accumulation. In accordance with the observed net starch accumulation in the e-N stage, a subset of putative starch synthases was up-regulated (GBSS / SSS in Figure S10b). Interestingly, their induction was more pronounced in the l-N stage (Figure S10b), where net starch accumulation was no longer observed (Figure 1b). Furthermore in this stage, a transcriptional up-regulation of the large AGPP subunit was observed (large AGPP in Figure S10b).

Up-regulated in a continuous manner in the e-N stage were all four starch phosphorylase candidates, as well as one of three glucan-water dikinase candidates (SP and GWD in Figure S10b). Their up-regulation was maintained throughout the l-N stage (Figure S10b). In the l-N stage, the remaining two glucan-water dikinase candidates and several amylase candidates were furthermore transcriptionally up-regulated (GWD and AMY in Figure S10b). These transcripts were supposed to be implicated in starch degradation [[53](#_ENREF_53)], yet the cellular net starch levels were only slightly decreasing in this stage (Fig. 1b). This discrepancy might be attributed to currently unknown posttranscriptional or posttranslational regulation steps of the corresponding catabolic enzymes. Additionally or alternatively, some candidates could potentially be implicated in starch synthesis. This has been proposed for plastidial starch phosphorylase in rice endosperm [[54](#_ENREF_54)], and is supported by the reduced starch content under –N conditions of a *C. reinhardtii* mutant which is defective in a plastidial starch phosphorylase (*sta4* strain) [[55](#_ENREF_55)].

None of the transcripts attributed to starch metabolism was found to be continuously decreased in abundance in the e-N stage (Figure S10b). A decrease in abundance was first noted in the l-N stage for the putative maltose excess transporter (MEX) transcript after 48 hours of N starvation (Figure S10b). The corresponding protein was implicated in starch breakdown in *A. thaliana* [[53](#_ENREF_53)]. A starch hyper-accumulation phenotype was observed by loss of MEX in *C. reinhardtii* [[56](#_ENREF_56), [57](#_ENREF_57)] and *A. thaliana* [[58](#_ENREF_58)], and starch levels of the *C. reinhardtii* mutant were approximately doubled compared to its parental strain under –N conditions [[56](#_ENREF_56)]. The observed down-regulation of MEX in *M. neglectum* could thus limit the extent of starch degradation in the l-N stage and thus aid in maintaining high starch levels in this stage. Alternatively, the down-regulation of MEX might be due to limited maltose concentrations provided that starch degradation is inhibited under –N conditions.

In the r+N stage, opposite transcriptional patterns for enzyme attributed to the same enzymatic step were observed (Figure S10b). Accordingly, those enzymes with increased transcript levels in the r+N stage might be implicated in starch degradation. This might apply to the putative disproportionating enzyme 2 (DPE2 in Figure S10b). DPE1 was in contrast transiently repressed in the r+N stage (Figure S10b). The specific up-regulation of DPE2 but not DPE1 in the r+N stage in *M. neglectum* might be in accordance with the likely role of DPE1 in starch synthesis in *C. reinhardtii* [[59](#_ENREF_59)]. In *C. reinhardtii*, starch levels were reduced by 90 % upon deletion of DPE1 (*sta11* mutant) [[59](#_ENREF_59)]. Opposite transcriptional regulation in the r+N stage was also noticed for two of three putative isoamylase genes, of which the first was up-regulated, while the second was down-regulated (Figure S10b). In *A. thaliana*, two out of four ISA enzymes were implicated in starch degradation [[53](#_ENREF_53)], which supports that the first putative ISA enzyme of *M. neglectum* might be implicated in starch degradation.

In summary, the majority of genes putatively implicated in the starch metabolism of *M. neglectum* were subjected to pronounced transcriptional changes in response to N availability. An interesting exception from this observation was the small catalytic AGPP subunit, which was found to be only transiently induced after two hours of –N conditions. Furthermore, it was proposed that DPE2 (putative fragment pair XLOC_019557 and XLOC_020099) and one putative isoamylase (XLOC_001619) might be implicated in starch degradation according to their transcript profiles.

**Additional File 1: Tables**

**Additional file 1: Table ST1: Significantly enriched GO terms in the set of genes highlighted in Figure 2e of the main article.**

See Figure S7 for expression profile plots of the respective sets.

Note that the number of genes which contributed to the analysis (first column) is smaller than the total number of genes shown in Figure 3e, because not all genes had GO terms associated and some were removed due to the filtering process of the “topGO” package [[60](#_ENREF_60)].

| **Part of set** | **GO term ID** | **description** | **# transcripts in universe with this term** | **# transcripts in the set with this term** | **p-value** |
| --- | --- | --- | --- | --- | --- |
| **up-regulated**  **e-N stage only**  **(n = 180)** | GO:0006270 | DNA replication initiation | 19 | 7 | 3e-6 |
|  | GO:0006457 | protein folding | 147 | 15 | 7e-6 |
|  | GO:0051258 | protein polymerization | 24 | 6 | 5e-5 |
|  | GO:0007017 | microtubule-based process | 151 | 7 | 2e-4 |
|  | GO:0010020 | chloroplast fission | 6 | 3 | 3e-4 |
|  | GO:0032955 | regulation of barrier septum assembly | 2 | 2 | 6e-4 |
|  | GO:0000918 | barrier septum site selection | 2 | 2 | 6e-4 |
|  | GO:0042542 | response to hydrogen peroxide | 18 | 4 | 8e-4 |
| **down-regulated**  **e-N stage only**  **(n = 270)** | GO:0006364 | rRNA processing | 139 | 33 | 9e-13 |
|  | GO:0015995 | chlorophyll biosynthetic process | 59 | 17 | 2e-11 |
|  | GO:0019288 | isopentenyl diphosphate biosynthetic process | 72 | 14 | 1e-6 |
|  | GO:0009220 | pyrimidine ribonucleotide biosynthetic process | 55 | 10 | 1e-5 |
|  | GO:0009073 | aromatic amino acid family biosynthetic process | 35 | 8 | 3e-5 |
|  | GO:0006164 | purine nucleotide biosynthetic process | 83 | 9 | 6e-5 |
|  | GO:0010501 | RNA secondary structure unwinding | 24 | 6 | 2e-4 |
|  | GO:0006412 | translation | 452 | 38 | 2e-4 |
|  | GO:0009423 | chorismate biosynthetic process | 4 | 3 | 2e-4 |
|  | GO:0009965 | leaf morphogenesis | 26 | 6 | 3e-4 |
|  | GO:0010027 | thylakoid membrane organization | 53 | 8 | 6e-4 |
|  | GO:0010027 | protein refolding | 12 | 4 | 7e-4 |
|  | GO:0009658 | chloroplast organization | 63 | 12 | 7e-4 |
|  | GO:0009902 | chloroplast relocation | 32 | 6 | 9e-4 |
|  | GO:0009902 | RNA methylation | 77 | 12 | 9e-4 |
| **up-regul.**  **l-N stage (n = 436)** | GO:0006468 | protein phosphorylation | 538 | 53 | 3e-5 |
|  | GO:0006633 | fatty acid biosynthetic process | 105 | 17 | 3e-5 |
|  | GO:0016042 | lipid catabolic process | 48 | 7 | 4e-4 |
|  | GO:0015996 | chlorophyll catabolic process | 11 | 4 | 3e-3 |
| **down-regul.**  **l-N stage only**  **(n = 584)** | GO:0000413 | protein peptidyl-prolyl isomerization | 51 | 13 | 1e-4 |
|  | GO:0010207 | photosystem II assembly | 41 | 11 | 2e-4 |
|  | GO:0010020 | chloroplast fission | 6 | 4 | 5e-4 |
|  | GO:0009926 | auxin polar transport | 10 | 5 | 5e-4 |
|  | GO:0090305 | nucleic acid phosphodiester bond hydrolysis | 117 | 17 | 6e-4 |
|  | GO:0016458 | gene silencing | 67 | 11 | 9e-4 |
| **up-regulated in both**  **e-N and l-N stages**  **(n = 412)** | GO:0015991 | ATP hydrolysis coupled proton transport | 35 | 11 | 2e-6 |
|  | GO:0006002 | fructose 6-phosphate metabolic process | 8 | 5 | 3e-5 |
|  | GO:0006526 | arginine biosynthetic process | 12 | 6 | 6e-5 |
|  | GO:0006096 | glycolytic process | 85 | 15 | 6e-5 |
|  | GO:0006099 | tricarboxylic acid cycle | 41 | 10 | 6e-5 |
|  | GO:0006863 | purine nucleobase transport | 6 | 4 | 1e-4 |
|  | GO:0005975 | carbohydrate metabolic process | 510 | 61 | 2e-4 |
|  | GO:0005980 | glycogen catabolic process | 4 | 3 | 7e-4 |
|  | GO:0046835 | carbohydrate phosphorylation | 21 | 6 | 8e-4 |
| **down-regulated in both**  **e-N and l-N stages**  **(n = 412)** | GO:0018298 | protein-chromophore linkage | 42 | 24 | 4e-24 |
|  | GO:0009765 | photosynthesis, light harvesting | 40 | 24 | 2e-22 |
|  | GO:0015979 | photosynthesis | 224 | 69 | 5e-14 |
|  | GO:0019344 | cysteine biosynthetic process | 71 | 18 | 2e-11 |
|  | GO:0009657 | plastid organization | 115 | 27 | 6e-10 |
|  | GO:0044085 | cellular component biogenesis | 433 | 41 | 8e-9 |
|  | GO:0010207 | photosystem II assembly | 41 | 12 | 2e-8 |
|  | GO:0006364 | rRNA processing | 139 | 21 | 1e-7 |
|  | GO:0019288 | isopentenyldiphosphate biosynthetic process | 72 | 12 | 1e-5 |
|  | GO:0019684 | photosynthesis, light reaction | 134 | 46 | 2e-5 |
|  | GO:0010027 | thylakoid membrane organization | 53 | 10 | 2e-5 |
|  | GO:0042549 | photosystem II stabilization | 6 | 4 | 3e-5 |
|  | GO:0009965 | leaf morphogenesis | 26 | 7 | 3e-5 |
|  | GO:0006782 | protoporphyrinogen IX biosynthetic process | 8 | 4 | 1e-4 |
|  | GO:0030003 | cellular cation homeostasis | 28 | 7 | 2e-4 |
|  | GO:0045038 | protein import into chloroplast thylakoid membrane | 4 | 3 | 2e-4 |
|  | GO:0009052 | pentose-phosphate shunt, non-oxidative branch | 4 | 3 | 2e-4 |
|  | GO:0009926 | auxin polar transport | 10 | 4 | 3e-4 |
|  | GO:0006655 | phosphatidylglycerol biosynthetic process | 19 | 5 | 5e-4 |
|  | GO:0000413 | protein peptidyl-prolyl isomerization | 51 | 8 | 5e-4 |
|  | GO:0006636 | unsaturated fatty acid biosynthetic process | 20 | 5 | 7e-4 |
|  | GO:0061077 | chaperone-mediated protein folding | 6 | 3 | 9e-4 |
| **up-regulated**  **r+N stage only**  **(n = 1809)** | GO:0006412 | translation | 452 | 320 | <e-30 |
|  | GO:0006364 | rRNA processing | 139 | 102 | 4e-24 |
|  | GO:0010027 | thylakoid membrane organization | 53 | 40 | 7e-15 |
|  | GO:0001510 | RNA methylation | 77 | 54 | 3e-13 |
|  | GO:0009220 | pyrimidine ribonucleotide biosynthetic process | 55 | 40 | 5e-13 |
|  | GO:0009073 | aromatic amino acid family biosynthetic process | 35 | 30 | 1e-10 |
|  | GO:0009902 | chloroplast relocation | 32 | 24 | 2e-9 |
|  | GO:0042254 | ribosome biogenesis | 172 | 131 | 1e-8 |
|  | GO:0006413 | translational initiation | 75 | 50 | 2e-8 |
|  | GO:0019344 | cysteine biosynthetic process | 71 | 38 | 1e-7 |
|  | GO:0006606 | protein import into nucleus | 26 | 19 | 2e-7 |
|  | GO:0006414 | translational elongation | 70 | 46 | 3e-7 |
|  | GO:0042793 | transcription from plastid promoter | 22 | 17 | 3e-7 |
|  | GO:0006446 | regulation of translational initiation | 15 | 14 | 4e-7 |
|  | GO:0006397 | mRNA processing | 86 | 47 | 6e-7 |
|  | GO:0001731 | formation of translation preinitiation complex | 10 | 10 | 6e-7 |
|  | GO:0006457 | protein folding | 147 | 68 | 2e-6 |
|  | GO:0019288 | isopentenyldiphosphate biosynthetic process | 72 | 37 | 2e-6 |
|  | GO:0015995 | chlorophyll biosynthetic process | 59 | 33 | 2e-6 |
|  | GO:0000105 | histidine biosynthetic process | 12 | 11 | 2e-6 |
|  | GO:0006189 | 'de novo' IMP biosynthetic process | 14 | 12 | 3e-6 |
|  | GO:0009765 | photosynthesis, light harvesting | 40 | 24 | 4e-6 |
|  | GO:0009793 | embryo development ending in seed dormancy | 50 | 28 | 4e-6 |
|  | GO:0015979 | photosynthesis | 224 | 99 | 5e-6 |
|  | GO:0006450 | regulation of translational fidelity | 14 | 12 | 9e-6 |
|  | GO:0034660 | ncRNA metabolic process | 291 | 209 | 1e-5 |
|  | GO:0016117 | carotenoid biosynthetic process | 47 | 27 | 1e-5 |
|  | GO:0010501 | RNA secondary structure unwinding | 24 | 16 | 1e-5 |
|  | GO:0006396 | RNA processing | 374 | 222 | 2e-5 |
|  | GO:0008033 | tRNA processing | 63 | 38 | 2e-5 |
|  | GO:0018298 | protein-chromophore linkage | 42 | 23 | 2e-5 |
|  | GO:0006626 | protein targeting to mitochondrion | 17 | 15 | 3e-5 |
|  | GO:0042026 | protein refolding | 12 | 10 | 3e-5 |
|  | GO:0006418 | tRNA amino acylation for protein translation | 79 | 63 | 3e-5 |
|  | GO:0044085 | cellular component biogenesis | 433 | 201 | 4e-5 |
|  | GO:0000398 | mRNA splicing, via spliceosome | 53 | 27 | 4e-5 |
|  | GO:0000462 | maturation of SSU-rRNA from tricistronic rRNA transcript | 11 | 9 | 9e-5 |
|  | GO:0006164 | purine nucleotide biosynthetic process | 83 | 40 | 1e-4 |
|  | GO:0030488 | tRNA methylation | 13 | 10 | 1e-4 |
|  | GO:0000413 | protein peptidyl-prolyl isomerization | 51 | 25 | 1e-4 |
|  | GO:0006094 | gluconeogenesis | 60 | 28 | 2e-4 |
|  | GO:0001522 | pseudouridine synthesis | 29 | 19 | 2e-4 |
|  | GO:0009965 | leaf morphogenesis | 26 | 16 | 2e-4 |
|  | GO:0042273 | ribosomal large subunit biogenesis | 13 | 11 | 2e-4 |
|  | GO:0006415 | translational termination | 10 | 9 | 2e-4 |
|  | GO:0016226 | iron-sulfur cluster assembly | 37 | 20 | 2e-4 |
|  | GO:0009658 | chloroplast organization | 63 | 40 | 3e-4 |
|  | GO:0006351 | transcription, DNA-templated | 407 | 120 | 3e-4 |
|  | GO:0006782 | protoporphyrinogen IX biosynthetic process | 8 | 7 | 3e-4 |
|  | GO:0008295 | spermidine biosynthetic process | 10 | 8 | 4e-4 |
|  | GO:0032259 | methylation | 320 | 126 | 4e-4 |
|  | GO:0010498 | proteasomal protein catabolic process | 53 | 18 | 6e-4 |
|  | GO:0030154 | cell differentiation | 91 | 27 | 6e-4 |
|  | GO:0009097 | isoleucine biosynthetic process | 13 | 9 | 7e-4 |
|  | GO:0044205 | 'de novo' UMP biosynthetic process | 5 | 5 | 8e-4 |
|  | GO:0006428 | isoleucyl-tRNA aminoacylation | 5 | 5 | 8e-4 |
|  | GO:0006434 | seryl-tRNA aminoacylation | 5 | 5 | 8e-4 |
|  | GO:0006438 | valyl-tRNA aminoacylation | 5 | 5 | 9e-4 |
|  | GO:0045893 | positive regulation of transcription, DNA-templated | 69 | 29 | 1e-3 |
| **down-regul.**  **r+N stage**  **(n = 473)** | GO:0015991 | ATP hydrolysis coupled proton transport | 35 | 13 | 2e-5 |
|  | GO:0006002 | fructose 6-phosphate metabolic process | 8 | 6 | 2e-5 |
|  | GO:0007033 | vacuole organization | 22 | 9 | 3e-4 |
|  | GO:0006099 | tricarboxylic acid cycle | 41 | 12 | 5e-4 |
|  | GO:0046835 | carbohydrate phosphorylation | 21 | 8 | 6e-4 |

| **Additional file 1: Table ST2: Overall alignment rates.**  Read pairs from the individual time points were aligned against the transcriptome encoded by the BRAKER1-annotation, in order to investigate whether surplus transcript information was contained in the three time points that were not used for annotation refinement by BRAKER1. | | | |
| --- | --- | --- | --- |
| **time point** | **million untrimmed fragments à 2x 100 nt** | **alignment rate of read pairs after trimming against the BRAKER1- transcriptome [%]** | **time point used for re-annotation by BRAKER1** |
| N_0 | 37.83 | 57.44 | yes |
| N_2 | 33.57 | 55.34 | yes |
| N_4 | 27.18 | 54.93 | yes |
| N_8 | 28.98 | 51.48 | no |
| N_24 | 28.15 | 52.30 | yes |
| N_48 | 30.47 | 50.76 | yes |
| N_56 | 37.01 | 49.43 | no |
| N_96 | 32.18 | 44.31 | yes |
| R_2 | 32.63 | 59.31 | yes |
| R_4 | 38.54 | 59.13 | yes |
| R_8 | 42.34 | 58.20 | no |
| R_14 | 29.23 | 56.16 | yes |

| **Additional file 1: Table ST3: Effect size values expressed as Cohen’s d of pairwise comparisons.**  The values for Cohen’s d were classified according to [[30](#_ENREF_30)], i.e. a small difference was given if Cohen’s d < 0.2, medium < 0.5, large < 0.8, very large < 1.3. | | | | |
| --- | --- | --- | --- | --- |
| **CDS length** | *C. reinhardtii* | *M. neglectum* | *N. gaditana* | *P. tricornutum* |
| *C. reinhardtii* | 0.00 | 0.48 | 0.55 | 0.41 |
| *M. neglectum* |  | 0.00 | 0.17 | 0.11 |
| *N. gaditana* |  |  | 0.00 | 0.30 |
| *P. tricornutum* |  |  |  | 0.00 |
| **exon length** | *C. reinhardtii* | *M. neglectum* | *N. gaditana* | *P. tricornutum* |
| *C. reinhardtii* | 0.00 | 0.11 | 0.34 | 1.02 |
| *M. neglectum* |  | 0.00 | 0.53 | 1.32 |
| *N. gaditana* |  |  | 0.00 | 0.51 |
| *P. tricornutum* |  |  |  | 0.00 |
| **intron length** | *C. reinhardtii* | *M. neglectum* | *N. gaditana* | *P. tricornutum* |
| *C. reinhardtii* | 0.00 | 0.04 | 0.21 | 0.55 |
| *M. neglectum* |  | 0.00 | 0.35 | 0.88 |
| *N. gaditana* |  |  | 0.00 | 0.30 |
| *P. tricornutum* |  |  |  | 0.00 |
| **# introns gene^-1^** | *C. reinhardtii* | *M. neglectum* | *N. gaditana* | *P. tricornutum* |
| *C. reinhardtii* | 0.00 | 0.39 | 0.94 | 1.10 |
| *M. neglectum* |  | 0.00 | 0.89 | 1.18 |
| *N. gaditana* |  |  | 0.00 | 0.44 |
| *P. tricornutum* |  |  |  | 0.00 |

**Additional File 1: Figures**

| **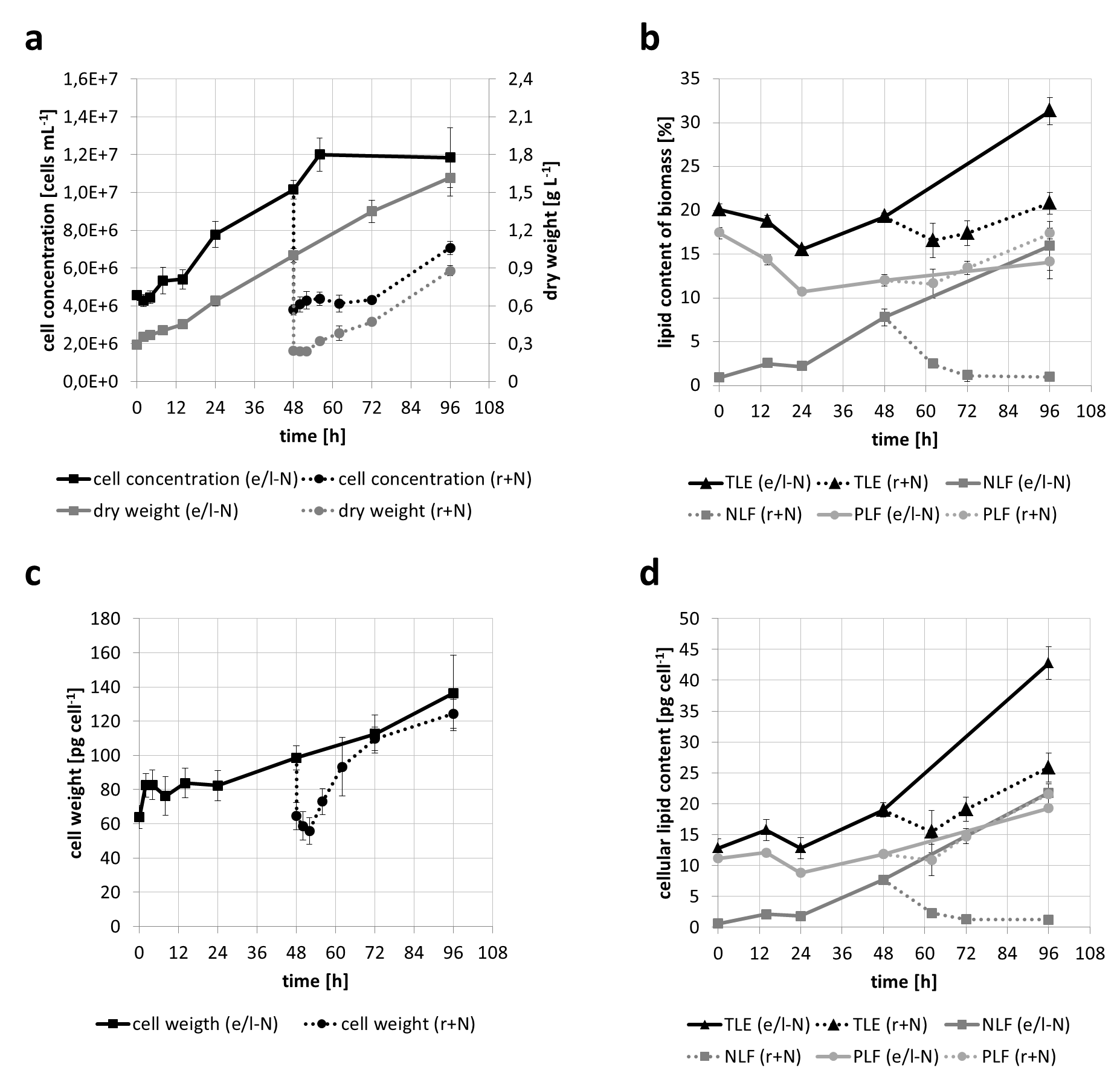** | | |
| --- | --- | --- |
| **Additional file 1: Figure S1: Cell growth parameters and dynamics of lipid accumulation of the transcriptome experiment.**  **(a)** Cell concentration (black, left y-axis) and dry biomass concentration (grey, right y-axis) for the transcriptome experiment (Figure 1, exp2), i.e. during four days of –N conditions (solid lines, the e-N and l-N stages), and from N resupply conditions after a 48 hour –N starvation period (dotted lines, the r+N stage).  **(b)** Gravimetrically determined total (TLE, total lipid extract, black), neutral (NLF, neutral lipid fraction, dark grey) and polar (PLF, polar lipid fraction, light grey) lipid content expressed as percent of biomass during the two nitrogen starvation stages (e/l-N, solid lines) and the N resupply stage (r+N, dotted lines).  **(c)** Cell weight obtained by dividing biomass concentration by cell concentration from (b).  **(d)** Similar to (b), except that the cellular lipid contents are shown.  In (b) – (e), mean values and standard errors (n = 2) are shown. The error bars in (c) and (d) were obtained by Gaussian error propagation, i.e. the squared relative standard deviations were summed, the square root of this value taken, and the obtained relative “error propagated” standard deviation multiplied with the mean to obtain the absolute standard deviation; this value was finally divided by square root of two to obtain the standard error. Relative standard deviations considered for (c) were from cell concentration and biomass concentration, and for (d) from cell concentration, biomass concentration and percent lipid content. |  |  |

| **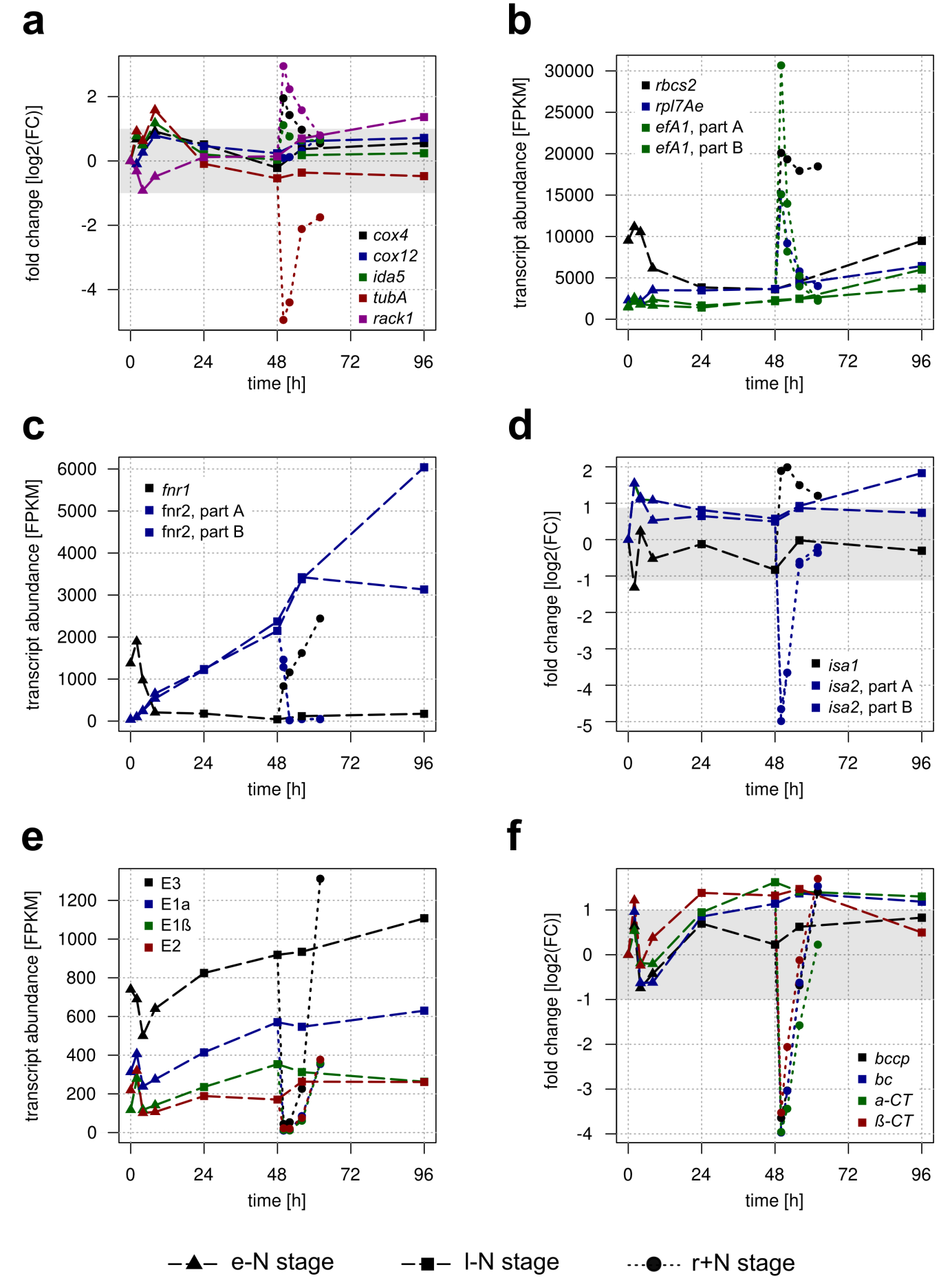** |
| --- |
| **Additional file 1: Figure S2: Detailed expression profiles of selected genes under nitrogen starvation (e-N and l-N stage) and nitrogen resupply (r+N stage).**  The y-axis either shows absolute transcript abundance as FPKM values, or relative transcript changes as log2-FC values.  **(a)** Relative transcript abundances of five housekeeping genes under –N (dashed line) and N resupply (dotted line) conditions, normalized to the reference time point N_0 from exponential growth conditions. The grey-shaded area indicates the threshold below which a gene was considered as not responsive at the respective time point (-1 ≤ log2 fold change (FC) ≤ 1). The locus identifiers are: *cox4* (cytochrome c oxidase 4), XLOC_003872; *cox12* (cytochrome c oxidase 12), XLOC_003091; *ida5* (actin), XLOC_003886; *tubA* (α-tubulin), XLOC_005203; *rack1* (receptor for activated C kinase 1), XLOC_011157.  **(b)** Absolute transcript levels of three highly expressed genes, which additionally exhibited a stable expression pattern under –N conditions. The *efA1* gene is likely present as a fragment pair. The locus identifiers are: *rbcS2* (RuBisCo small subunit 2), XLOC_007679; *rpl7Ae* (large subunit of the 60S cytosolic ribosome), XLOC_000987; *efA1* part A (elongation factor A), XLOC_012699; *efA1* part B, XLOC_005939.  **(c)** Absolute transcript levels of two putative ferredoxin-NADP^+^-reductase (FNR) genes. The second fnr gene is likely present as a fragment pair. The locus identifiers are: *fnr1*, XLOC_001499; *fnr2* part A, XLOC_015550; *fnr2* part B, XLOC_016383.  **(d)** Relative transcript abundances of two putative isoamylase (ISA) genes. The second *isa* gene is likely present as a fragment pair. The locus identifiers are: *isa1*, XLOC_001619; *isa2* part A, XLOC_004804; *isa2* part B, XLOC_012040.  **(e)** Absolute transcript levels of the putative subunits of the plastidial pyruvate dehydrogenase complex. The locus identifiers are: E3 (dihydrolipoyl dehydrogenase), XLOC_012332; E1α (α subunit of pyruvate dehydrogenase), XLOC_010047; E1β, XLOC_005527; E2 (dihydrolipoyl transacetylase), XLOC_010276.  **(f)** Relative transcript abundances of the putative subunits of the acetyl-CoA carboxylase complex. Except for biotin carboxylase (BC), all genes are likely present as fragment pairs; of those, only one fragment is shown. The locus identifiers are: *bccp* (biotin carboxylase carrier protein), XLOC_017895; *bc*, XLOC_000889; *α-CT* (α subunit of carboxyltransferase), XLOC_017485; *β-CT*, XLOC_015237. |

| 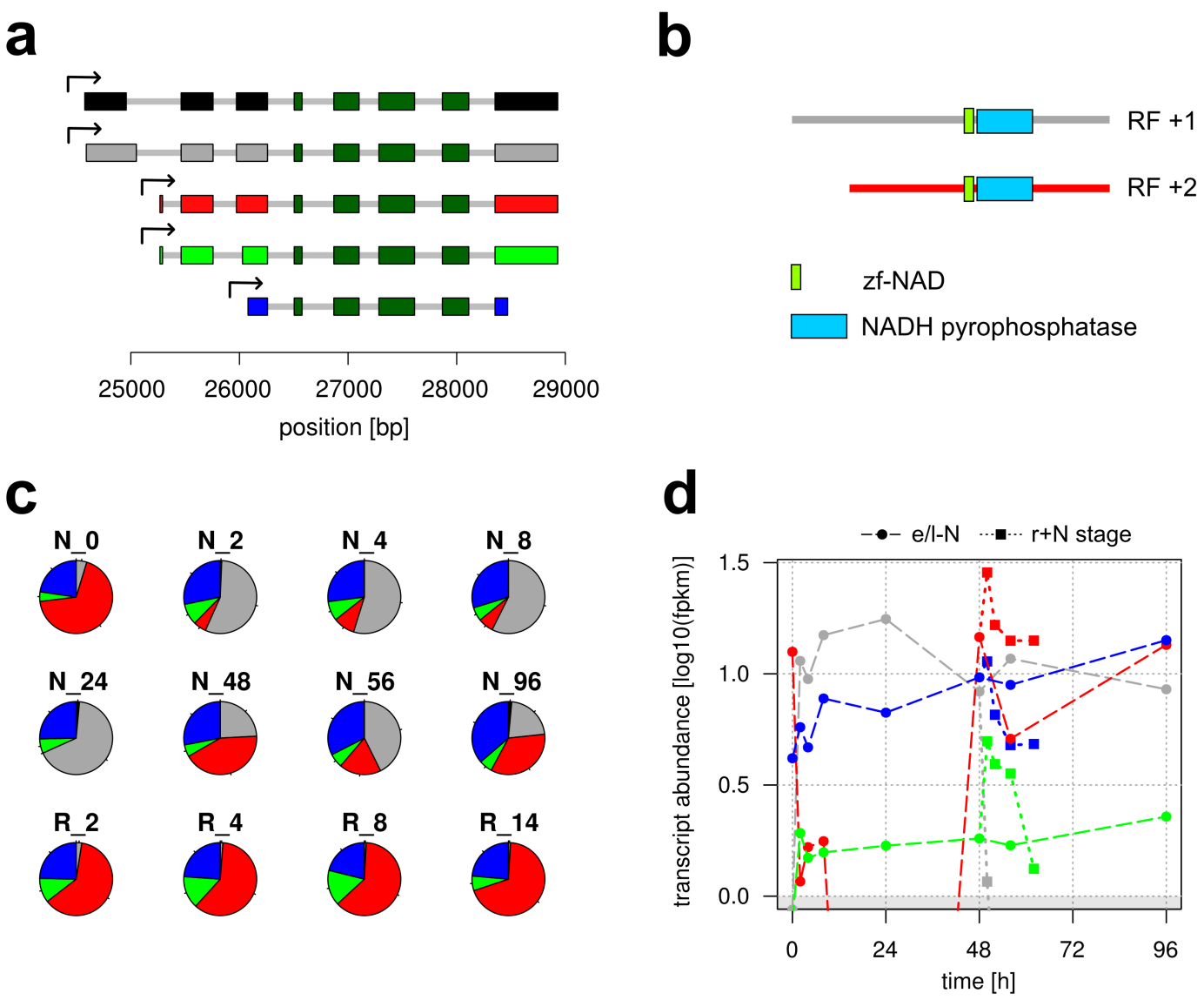 |
| --- |
| **Additional file 1: Figure S3: Evidence for dominant isoform switching during nitrogen starvation resulting in different transcript lengths.**  **(a)** Predicted isoforms for locus XLOC_001473. Transcription start sites are indicated by black arrows. Exons are shown as colored boxes, whereas introns are represented by grey lines. Exons shared by all isoforms are colored dark green.  **(b)** Putative domain structure of two isoforms putatively exhibiting dominant isoform switching during -N conditions. Prediction was performed by NCBI conserved domain search web interface [[15](#_ENREF_15)].  **(c)** Pie chart of relative isoform abundances at the respective time points of –N and N resupply.  **(d)** Absolute isoform abundances (FPKM values) at the respective time points of –N and N resupply on a half-log scale. The grey-shaded area indicates FPKM values < 1.0. |

| 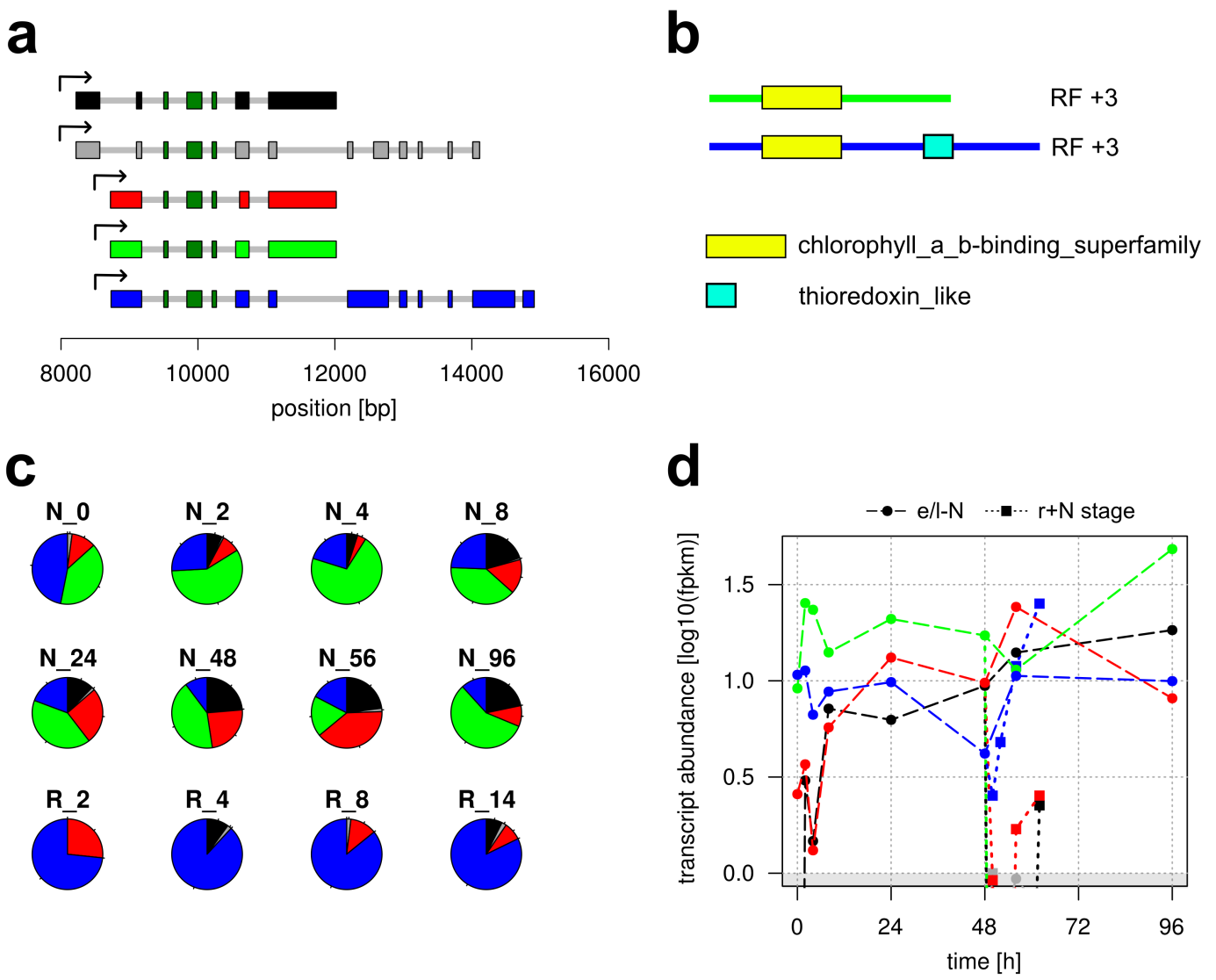 |
| --- |
| **Additional file 1: Figure S4: Evidence for dominant isoform switching during nitrogen starvation resulting in altered domain structures.**  **(a)** Predicted isoforms for locus XLOC_002371. Transcription start sites are indicated by black arrows. Exons are shown as colored boxes, whereas introns are represented by grey lines. Exons shared by all isoforms are colored dark green.  **(b)** Putative domain structure of two isoforms putatively exhibiting dominant isoform switching during -N conditions. Prediction was performed by NCBI conserved domain search web interface [[15](#_ENREF_15)].  **(c)** Pie chart of relative isoform abundances at the respective time points of –N and N resupply.  **(d)** Absolute isoform abundances (FPKM values) at the respective time points of –N and N resupply on a half-log scale. The grey-shaded area indicates FPKM values < 1.0. |

| 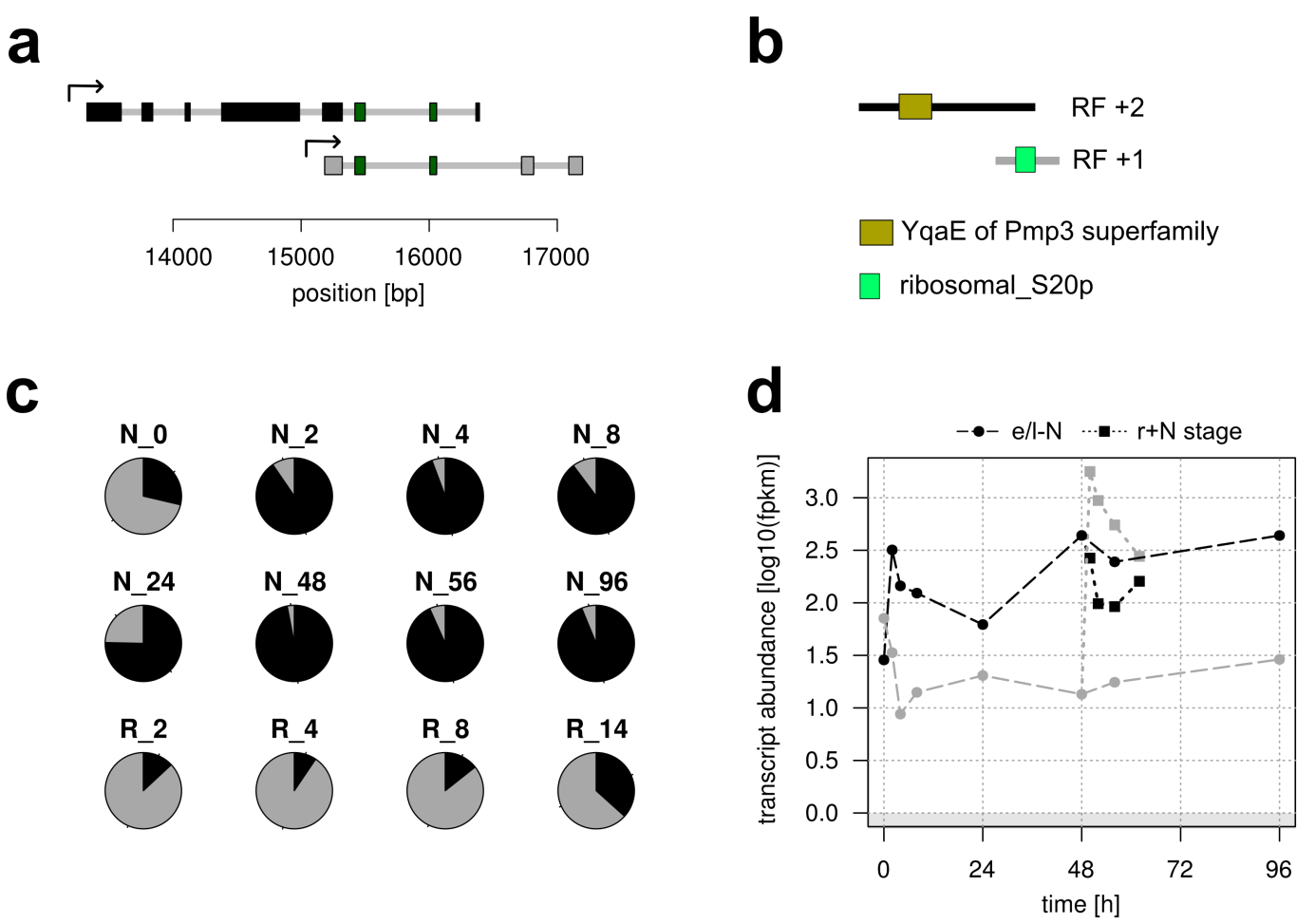 |
| --- |
| **Additional file 1: Figure S5: Evidence for dominant isoform switching during nitrogen starvation resulting in completely different domains.**  **(a)** Predicted isoforms for locus XLOC_007101. Transcription start sites are indicated by black arrows. Exons are shown as colored boxes, whereas introns are represented by grey lines. Exons shared by all isoforms are colored dark green.  **(b)** Putative domain structure of two isoforms putatively exhibiting dominant isoform switching during -N conditions. Prediction was performed by NCBI conserved domain search web interface [[15](#_ENREF_15)].  **(c)** Pie chart of relative isoform abundances at the respective time points of –N and N resupply.  **(d)** Absolute isoform abundances (FPKM values) at the respective time points of –N and N resupply on a half-log scale. The grey-shaded area indicates FPKM values < 1.0. |

| 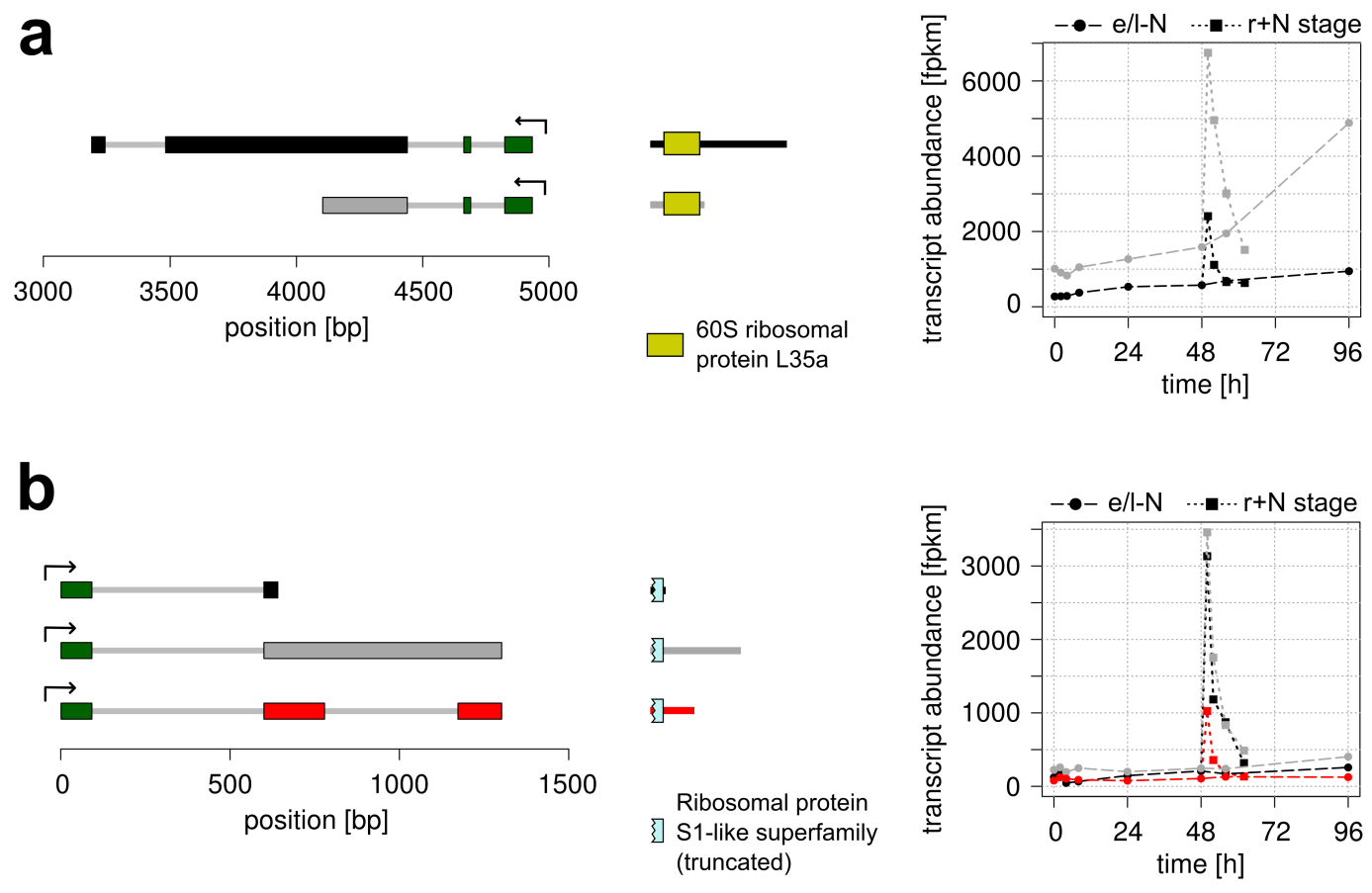 |
| --- |
| **Additional file 1: Figure S6: Evidence for alternative splicing of highly expressed transcripts in the transcriptome of *M. neglectum*.**  **(a)** Annotation and expression of the locus XLOC_003672. Predicted isoforms are shown on the left, in which exons and introns are shown as colored boxes and grey lines, and transcription start sites as a black arrow. Dark green indicates exons shared between different isoforms. In the middle, the predicted domain structure by NCBI conserved domain search web interface [[15](#_ENREF_15)] is shown. On the right, isoform abundances values at the respective time points of –N and N resupply conditions are shown.  **(b)** Annotation and expression of the locus XLOC_012610 as in (a). |

| 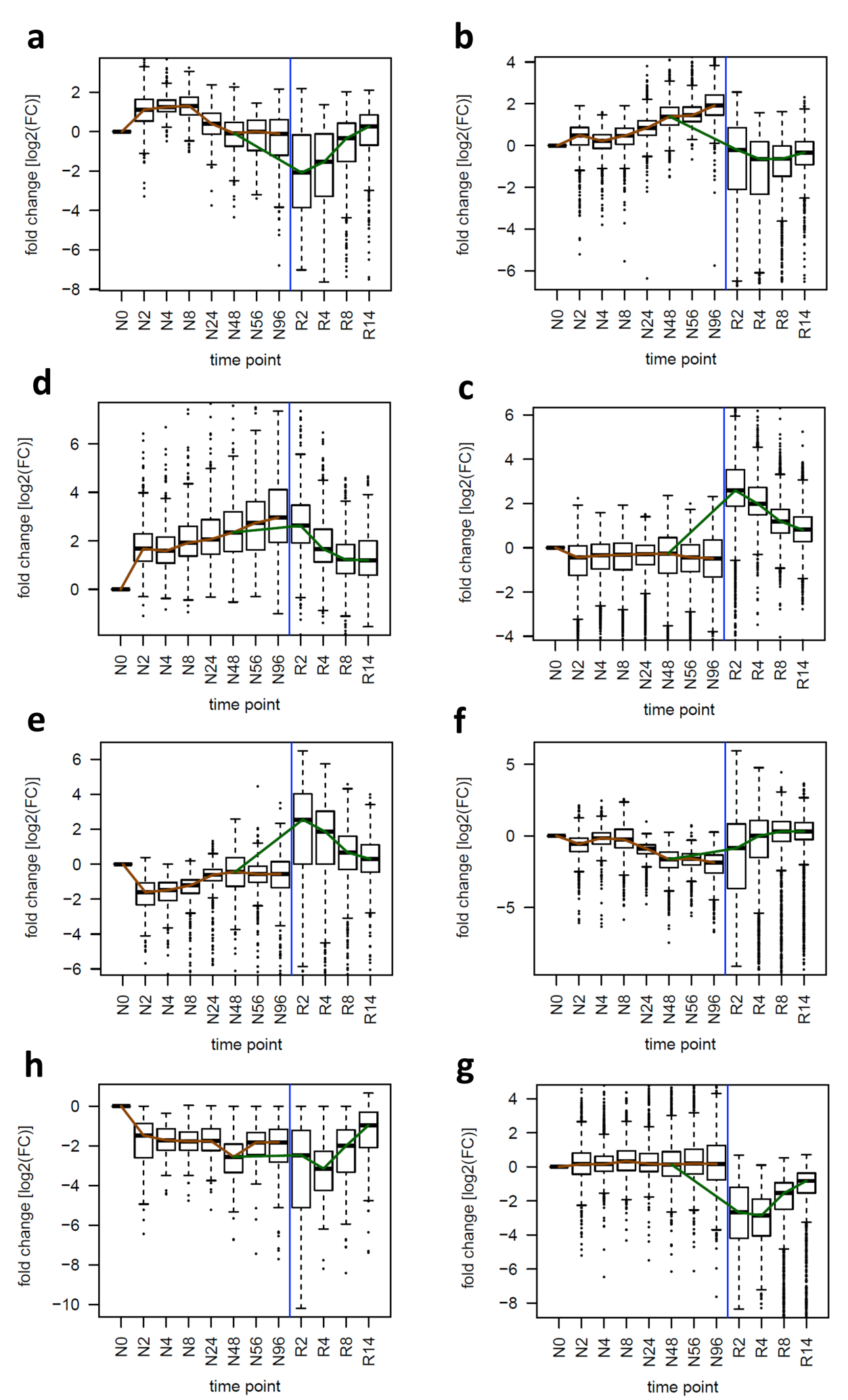 |
| --- |
| **Additional file 1: Figure S7: Expression profiles of the gene sets shown in Figure 3e of the main article.**  **(a)** genes with transcriptional induction restricted to the early –N (e-N) stage,  **(b)** genes with transcriptional induction restricted to the late –N (l-N) stage,  **(c)** genes with transcriptional induction in both, the e-N and l-N stages,  **(d)** genes with transcriptional induction restricted to the N resupply (r+N) stage,  **(e)** genes with transcriptional repression restricted to the e-N stage,  **(f)** genes with transcriptional repression restricted to the l-N stage,  **(g)** genes with transcriptional repression in both, the e-N and l-N stage,  **(h)** genes with transcriptional repression restricted to the r+N stage.  The distribution of FC values from each time point is shown as a box-whisker plot to highlight the trend in expression; for a description of the box-whisker plots. The brown line connects the median FC at the respective time points of –N (the e-N and l-N stages), and the green line those from N resupply after a 48 hour N starvation period (the r+N stage). The different N availability phases are separated by a blue line.  Significantly enriched GO terms are given in Table ST1.  Box-whisker plots in (a–h): the thick lines represent the median values, the grey box represents the interval between the first and third quartile, the two whiskers indicate the respective 1.5x interquartile ranges, and open circles mark the outliers. |

|  |
| --- |
| **Additional File 1: Figure S8: Usability of different nitrogen sources by *M. neglectum*.**  *M. neglectum* was grown mixotrophically in tris-phosphate-glucose medium [[61](#_ENREF_61)] supplemented with 12 mM of the respective N source. Cultivation was performed at room temperature with gentle shaking at 200 µE. The higher cell concentration for growth in urea was likely due to twice the N amount, because one urea molecule contains two N atoms. Mean values and standard errors (n = 3) are shown. |

| 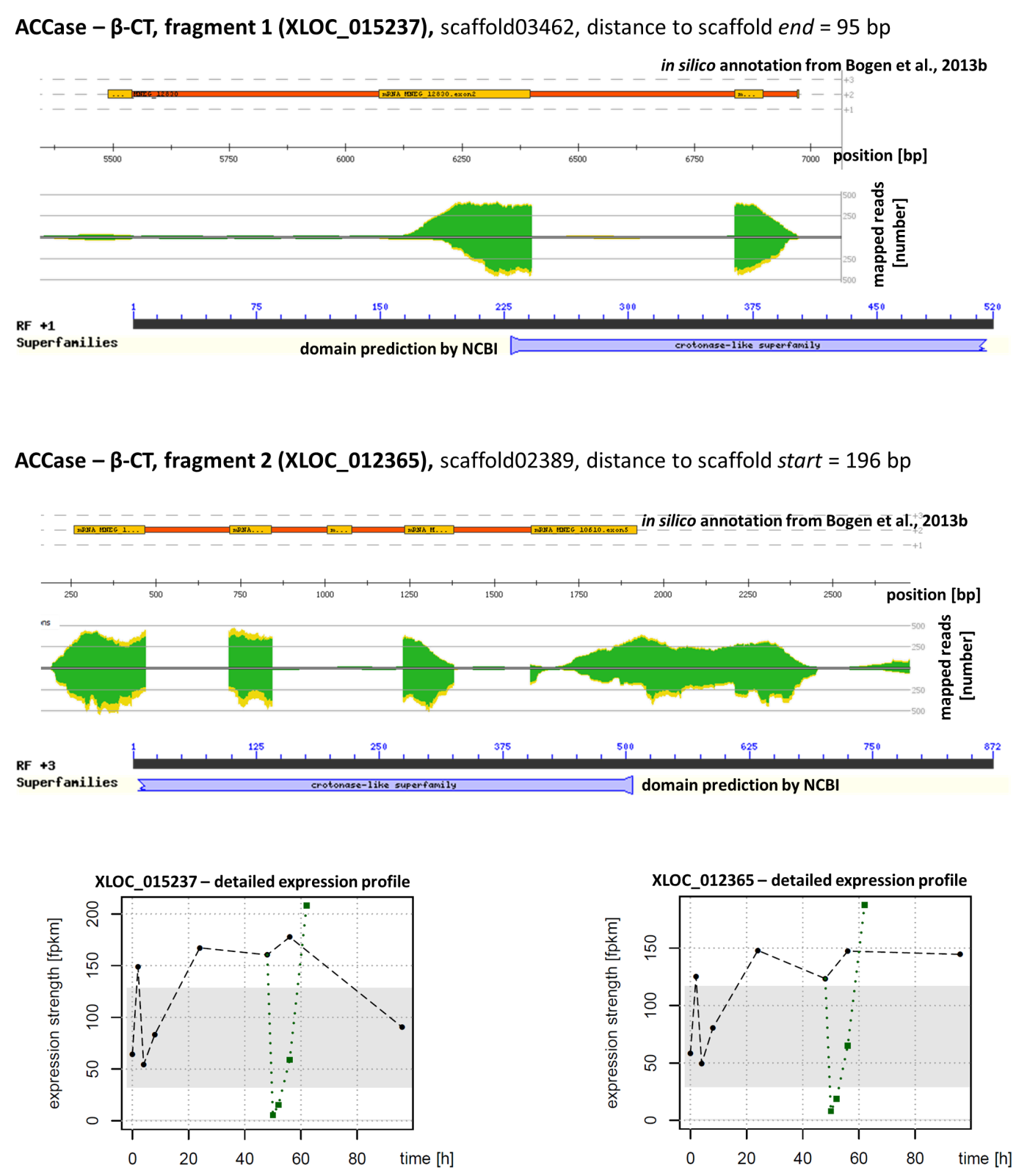 |
| --- |
| **Additional file 1: Figure S9: Strategy for the identification of putative gene fragments.**  As an example for gene fragmentation, the beta carboxyltransferase subunit (β-CT) of the multimeric acetyl-CoA-carboxylase complex (ACCase) is shown. The two genes had the same annotation (crotonase-like superfamily) and were located at the scaffold boundaries (close to the end, top; and close to the start, bottom). Furthermore, their predicted domain structure fitted together visually (bottom part of each subfigure) and both genes had very similar expression profiles (right). Since additionally the Pearson correlation between the expression profiles of the two genes was 0.94, which was larger than the threshold of 0.90, these two genes were therefore defined as fragments of a hypothetical, larger, full-length β-CT gene.  In the expression profile plots, the black dashed line represents the transcript level under –N conditions (the e-N and l-N stages), while the dotted green line represents the transcript level upon N resupply (the r+N stage). The grey box indicates the range in which a gene was classified as not responsive, i.e. -1 ≤ log2-FC ≤ 1.  Note that the pattern of expression is very similar for all time points, except for the last time point after four days of –N (N_96). For this time point, the fragment close to the 3’ end (plot in the lower part of the figure) exhibited higher expression. This was consistently observed specifically for the N_96 time point for the 3’ fragments from putative fragment pairs.  The read mapping visualization was obtained by ReadXplorer (version 2.1.0) [[62](#_ENREF_62), [63](#_ENREF_63)]. |

| 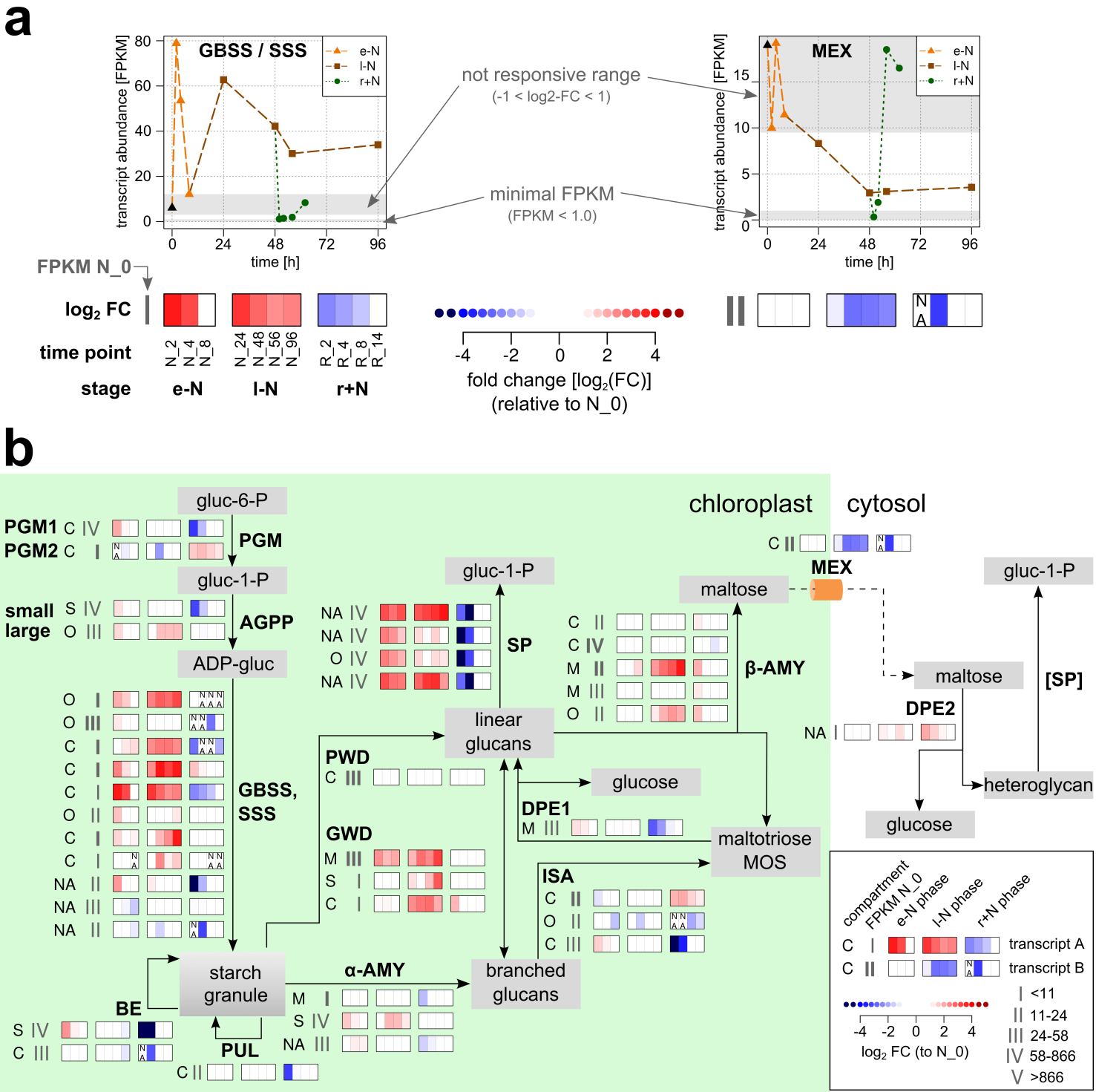 |
| --- |
| **Additional file 1: Figure S10: Schematic representation of the putative enzymatic steps of starch metabolism in *M. neglectum*, including the transcriptional response to N starvation (stages e-N and l-N) or N resupply (stage r+N)**  **(a)** Illustration of the interpretation of the expression pictograms used to display transcriptional regulation in Figures 4, 5 and this figure (b). The transcript abundances of a putative starch synthase gene in the e-N, l-N and r+N stages are shown on the left top, and the corresponding transcriptional regulation related to the reference time point N_0 on the left bottom. The grey box in the upper plot indicates the range where the absolute log2-FC (fold change) is less than 1, and for which the corresponding box in the expression pictogram on the bottom is colored white.  On the right side, the transcript abundance and expression pictogram plot illustrate the occurrence of the tag “NA”, which is added to a white box of a pictogram if the transcript abundance (FPKM) value at the respective time point is less than 1.0. This is shown as the smaller, lower grey box in the upper right plot; note that the larger, upper grey box indicates the range where log2-FC ≤ 1. Interpretation of the tag “NA” depends on the context. For the case of the putative MEX transcript, the transcript is repressed after four hours of N resupply in the r+N stage (R_4 time point), so that the tag “NA” for the previous time point (R_2) underscores a tight transcriptional regulation of the MEX transcript in the r+N stage. Note, however, that in other cases, the tag “NA” indicates generally low abundance, such as for the first putative starch synthase transcript in (b), which has FPKM values of 1.4, 2.4, 1.0, 0.2, 0.7 at the time points N_0, R_2, R_4, R_8 and R_14, respectively.  **(b)** Reconstruction of the putative starch metabolism from *M. neglectum* by genomic and transcriptomic data. Enzymatic steps are represented by solid arrows and transport processes by dashed lines. Each step has at least one transcript associated, and the putative localization is indicated on the left (C = chloroplast, M = mitochondrion, O = other, S = secretory pathway, NA = not available due to truncation).  The section with the grey Roman numerals next to the predicted localization shows the binned transcript abundance at the reference time point N_0. The number of vertical bars indicates the category, i.e. one bar = below median abundance, two bars = around median abundance, three bars = upper quartile abundance, four bars = upper interquartile range, five bars = high abundance outlier; see also legend on the bottom right and Figure 2d for the distribution of FPKM values. Bold Roman numerals indicate that the respective gene is likely not fragmented, whereas normal font style indicates that only the transcript abundance of the putative fragment containing the 5’ end is shown.  The transcription profile of each enzyme is represented by three color boxes, representing the three different cultivation stages investigated in this work (e-N, l-N, r+N). In each of the boxes, the transcriptional regulation at the individual harvesting time points relative to time point zero (N_0) is indicated by color-coded bars (red = up-regulation, blue = down-regulation compared to N_0). White bars are shown if the change in relative transcript abundance was between 50 and 200 % (absolute log2-FC < 1). The tag “NA” (not available) is added if the absolute transcript abundance (as FPKM) at that time point was less than 1.0, which was set as the minimum threshold for reliable transcript abundance estimation.  The full annotations of the corresponding genes are given in Additional file 5.  **Abbreviations:** AGPP, ADP-glucose pyrophosphorylase; AMY, amylase; BE, branching enzyme; DPE, disproportionating enzyme; GBSS, granule-bound starch synthase; GWD, glucan-water dikinase; ISA, isoamylase-type debranching enzyme; MEX, maltose exporter; PGM, phosphoglucomutase; PUL, pullulanase-type debranching enzyme; PWD, phosphoglucan-water dikinase; SP, starch phosphorylase; SSS, soluble starch synthase.  ADP, adenosindiphosphate; gluc, glucose; MOS, maltooligosaccharides; P, phosphate. |

|  |
| --- |
| **Additional file 1: Figure S11: Usability of different carbon sources by *M. neglectum*.**  *M. neglectum* was grown with or without addition of an reduced external carbon source in ProF medium [[1](#_ENREF_1)]. Cultivation was performed at room temperature with gentle shaking at ~50 µE. It became apparent that *M. neglectum* can use glucose as sole carbon source, but acetate only to a limited extent. Mean values and standard errors (n = 2) are shown. |

| 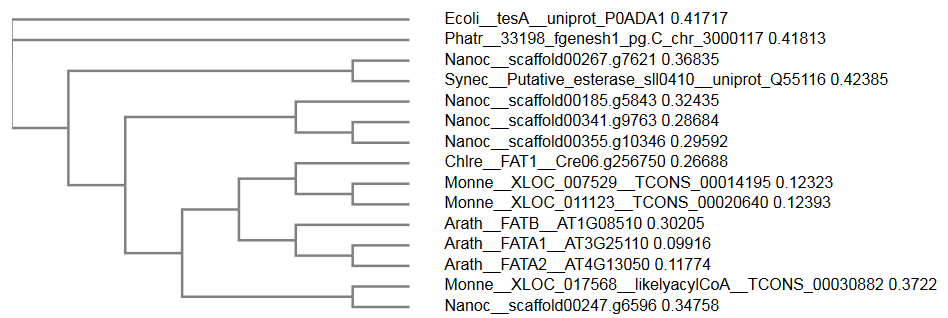 |
| --- |
| **Additional file 1: Figure S12: Phylogenetic tree of different thioesterase protein sequences.**  The alignment was performed with ClustalOmega [[64](#_ENREF_64)] and the tree obtained by the function “Send to Simple_Phylogeny” with default settings (“Neighbour-joining” as clustering method).  Arath, *Arabidopsis thaliana*; Chlre, *Chlamydomonas reinhardtii*; Ecoli, *Escherichia coli*; Monne, *Monoraphidium neglectum*; Nanoc, *Nannochloropsis oceanica*; Synec, *Synechocystis* sp. strain PCC 6803. |

| 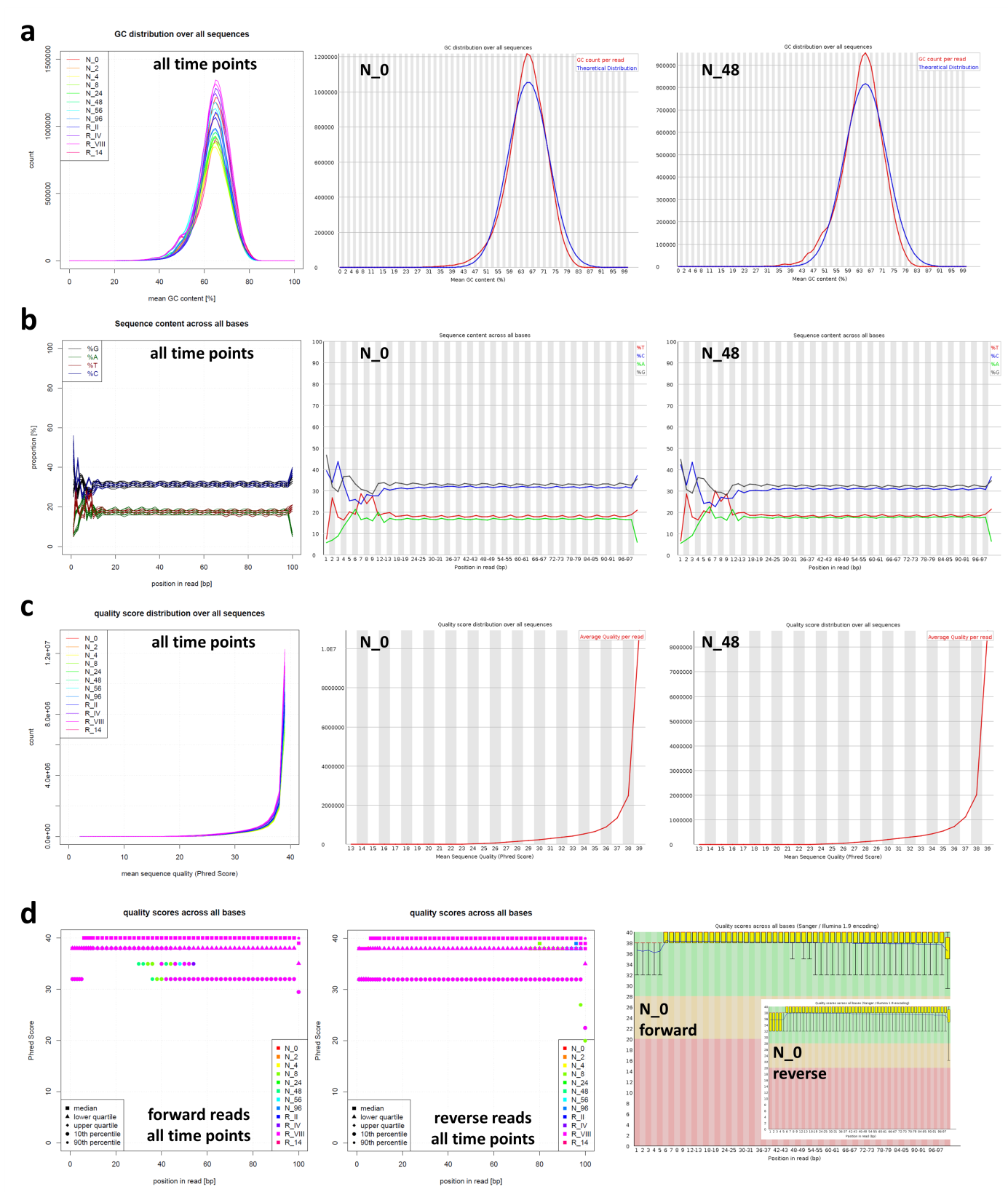 |
| --- |
| **Additional file 1: Figure S13: Quality of reads after filtering via FastQC [**[**65**](#_ENREF_65)**].**  **(a)** Distribution of GC content of all sequences.  **(b)** Proportion of the four bases for each position over all sequences. Note that a differentiation between different time points is not shown for this analysis; in contrast, each of the four bases (A, T, G, C) is color-coded.  **(c)** Distribution of Phred Scores of all sequences.  **(d)** Overview of the quality ranges (as Phred Score) across all bases at each position.  For each subfigure, a combined plot of all FASTQ-files from all time points is shown on the left, while individual plots produced by FastQC are exemplarily shown in the middle (time point N_0, lane 1, forward reads) and on the right (time point N_48, lane 1, forward reads). Note that for each time point, four FASTQ-files were available: one for each of the two read orientations (forward and reverse), as well as one from each of the two lanes. Since each file was analyzed separately, each color (i.e. time point) has attributed four analysis values, i.e. four same-colored lines e.g. in (a). |

| 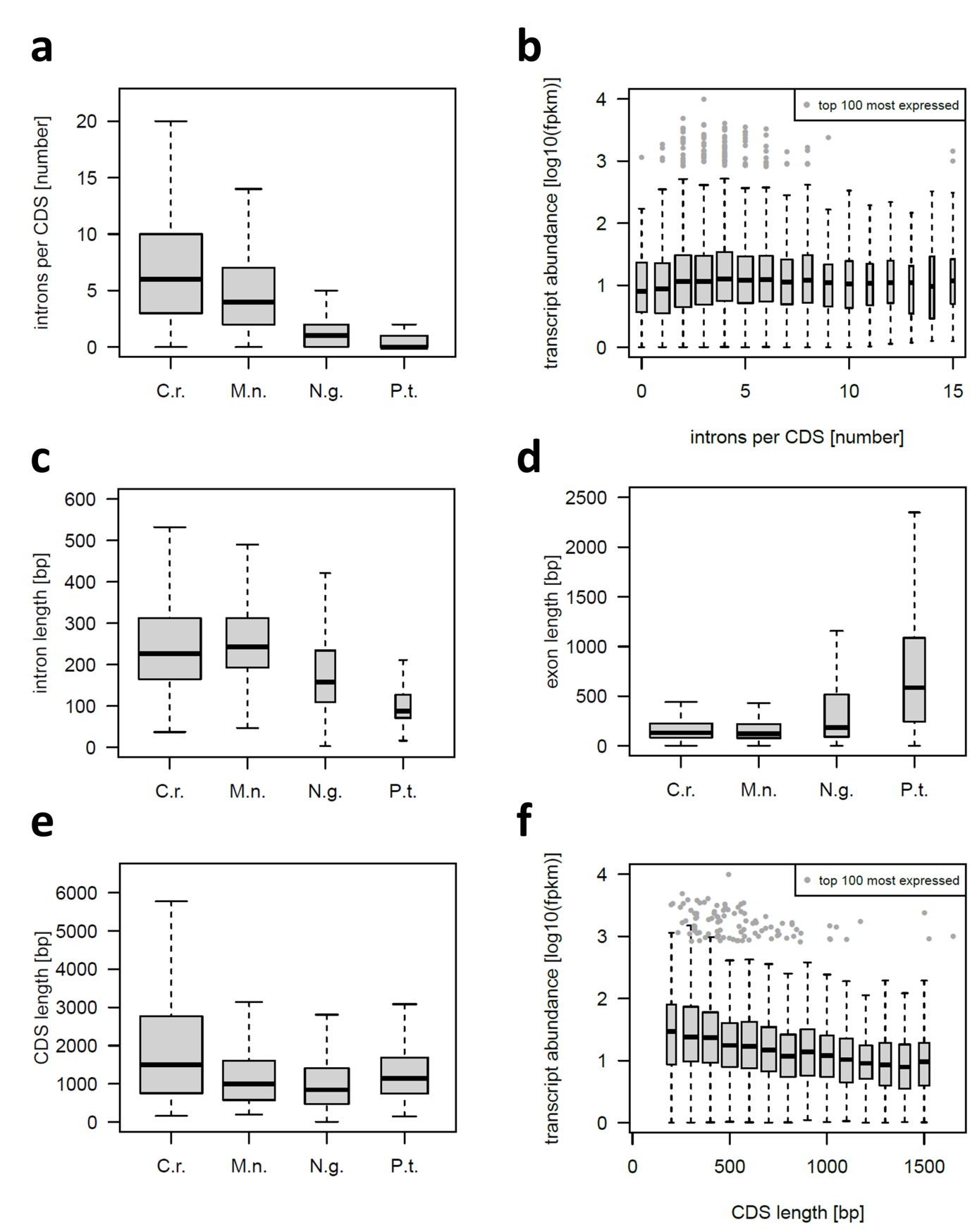 |
| --- |
| **Additional file 1: Figure S14: Quantitative composition of CDS from the chlorophyceae *C. reinhardtii* and *M. neglectum*, the eustigmatophyceae *N. gaditana* and the diatom *P. tricornutum*.**  **(a)** Number of introns per gene in the four microalgae; n = 17,737; 12,435; 8,818 and 10,025 loci from *C. reinhardtii*, *M. neglectum*, *N. gaditana* and *P. tricornutum*, respectively.  **(b – d)** Distribution of the length (base pairs; bp) of all introns **(b)**, exons **(c)** and coding sequences **(d)** of all genes; n = 132,306; 61,051; 13,508; 7,725 introns and n = 150,043; 73,486; 22,326 and 17,750 exons from *C. reinhardtii*, *M. neglectum*, *N. gaditana* and *P. tricornutum*, respectively; the number of coding sequences are equal to (a).  Box-whisker plots in (a–d): the thick lines represent the median values, the grey box represents the interval between the first and third quartile, the two whiskers indicate the respective 1.5x interquartile ranges, and open circles mark the outliers. The size of the box is scaled according to square root of the sample size. See Table ST3 for pairwise Cohen’s d values to evaluate effect size. |

| 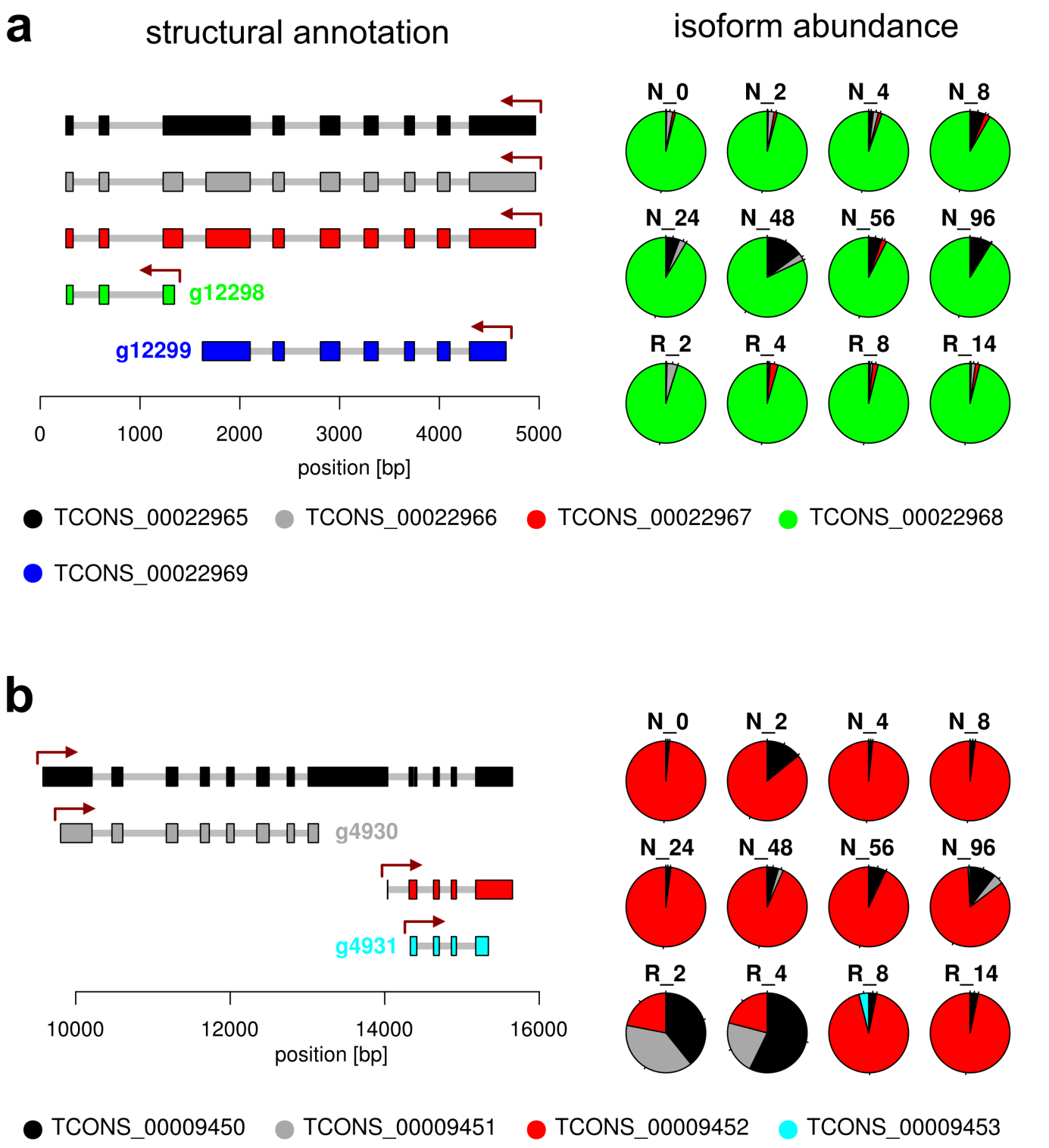 |
| --- |
| **Additional file 1: Figure 15: Resolving transcript fusion on the level of isoforms.**  **(a)** The left part shows the structural annotation of the locus XLOC_012520. This locus likely comprises two individual genes, i.e. g12298 (putative PGK) and g12299 (unknown function). The red arrows indicate the putative transcription start sites. The relative contribution of the individual isoforms on the overall locus-FPKM value for each time point is depicted on the right. It becomes apparent that the isoform TCONS_00022968 is the dominant isoform at all time points. Therefore, the locus-FPKM value can be used as an approximation for the transcript abundance of the putative PGK gene.  **(b)** The left part shown the structural annotation of the locus XLOC_004946, which is likely a fusion of two individual genes, i.e. g4930 (putative PGK) and g4931 (a putative DNA binding protein). The isoform abundance plot on the right reveals that the transcript levels of the second gene are more abundant than those of the first. Therefore, the locus-FPKM values cannot be used to approximate the expression profile of the putative PGK gene. |

| 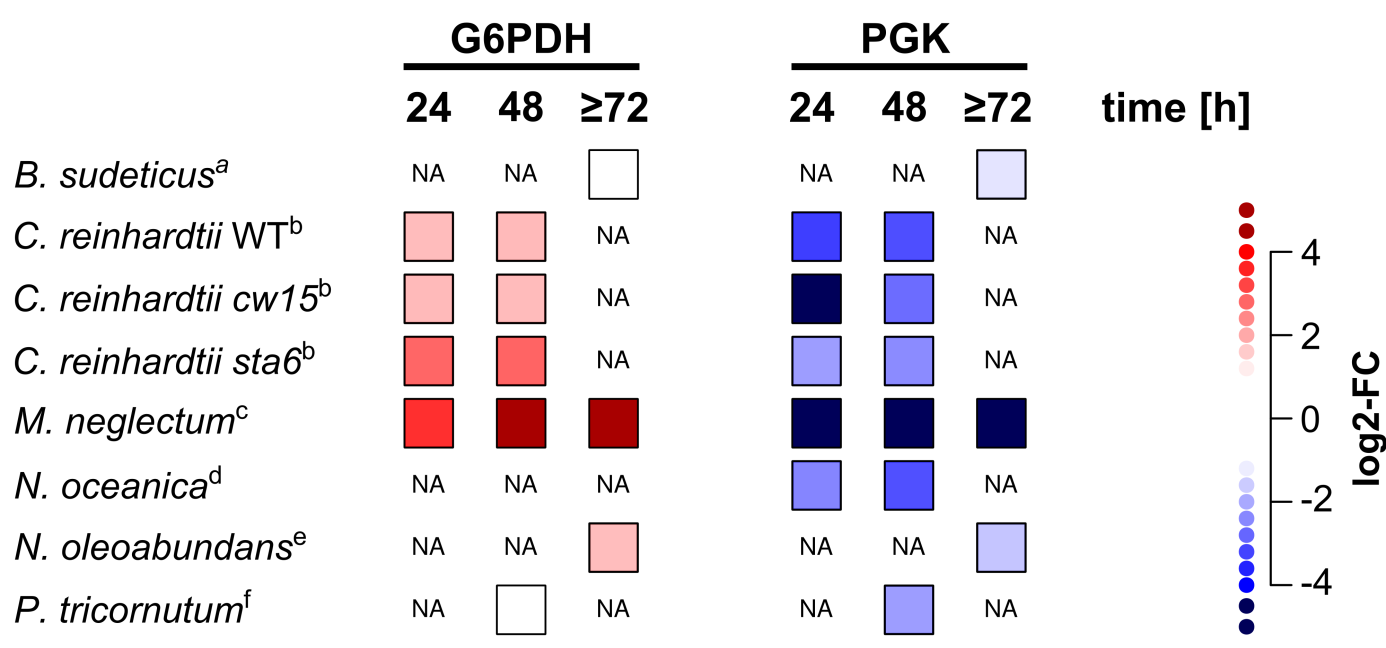 |
| --- |
| **Additional file 1: Figure 16: Transcriptional regulation of glucose 6-phosphate dehydrogenase and phosphoglycerate kinase under nitrogen starvation in different microalgae.**  The transcript data was obtained by querying the transcriptome database developed in this study. Different time points of –N conditions are indicated at the top. G6PDH, glucose 6-phosphate dehydrogenase; NA, transcript data not available; PGK, phosphoglycerate kinase.  References for the transcript data are: ^a^ [[21](#_ENREF_21)], ^b^ [[17](#_ENREF_17)], ^c^ this study, ^d^ [[18](#_ENREF_18)], ^e^ [[20](#_ENREF_20)], ^f^ [[19](#_ENREF_19)]. |

|  |
| --- |
| **Additional file 1: Figure S17: Distribution of transcription factor families of transcription factors which were up- or down-regulated in the l-N stage in *M. neglectum*.**  193 putative transcription factors were identified using the Plant Transcription Factor Database [[38](#_ENREF_38)]. Shown are those with an absolute mean-FC greater than 1 in the late –N (l-N) stage. For the complete list of putative transcription factors, see Additional file 4. |

**References**

1. Bogen C, Al-Dilaimi A, Albersmeier A, Wichmann J, Grundmann M, Rupp O, Lauersen KJ, Blifernez-Klassen O, Kalinowski J, Goesmann A: **Reconstruction of the lipid metabolism for the microalga Monoraphidium neglectum from its genome sequence reveals characteristics suitable for biofuel production.** *BMC genomics* 2013, **14:**926.

2. Hoff KJ, Lange S, Lomsadze A, Borodovsky M, Stanke M: **BRAKER1: Unsupervised RNA-Seq-Based Genome Annotation with GeneMark-ET and AUGUSTUS.** *Bioinformatics* 2015.

3. Stanke M, Waack S: **Gene prediction with a hidden Markov model and a new intron submodel.** *Bioinformatics* 2003, **19:**ii215-ii225.

4. Besemer J, Borodovsky M: **GeneMark: web software for gene finding in prokaryotes, eukaryotes and viruses.** *Nucleic acids research* 2005, **33:**W451-W454.

5. Barnett DW, Garrison EK, Quinlan AR, Strömberg MP, Marth GT: **BamTools: a C++ API and toolkit for analyzing and managing BAM files.** *Bioinformatics* 2011, **27:**1691-1692.

6. Trapnell C, Roberts A, Goff L, Pertea G, Kim D, Kelley DR, Pimentel H, Salzberg SL, Rinn JL, Pachter L: **Differential gene and transcript expression analysis of RNA-seq experiments with TopHat and Cufflinks.** *Nature protocols* 2012, **7:**562-578.

7. Langmead B, Salzberg SL: **Fast gapped-read alignment with Bowtie 2.** *Nat Methods* 2012, **9**.

8. Merchant S, Prochnik S, Vallon O, Harris E, Karpowicz S, Witman G, Terry A, Salamov A, Fritz-Laylin L, Marechal-Drouard L, other s: **The Chlamydomonas genome reveals the evolution of key animal and plant functions.** *Science* 2007, **318**.

9. Radakovits R, Jinkerson RE, Fuerstenberg SI, Tae H, Settlage RE, Boore JL, Posewitz MC: **Draft genome sequence and genetic transformation of the oleaginous alga Nannochloropsis gaditana.** *Nature communications* 2012, **3:**686.

10. Bowler C, Allen AE, Badger JH, Grimwood J, Jabbari K, Kuo A, Maheswari U, Martens C, Maumus F, Otillar RP: **The Phaeodactylum genome reveals the evolutionary history of diatom genomes.** *Nature* 2008, **456:**239-244.

11. Team RC: **R: A Language and Environment for Statistical Computing.** 2016.

12. Cohen J: **Statistical Power Analysis for the Behavioral Sciences.** *Routledge* 1988.

13. Trapnell C, Williams B, Pertea G, Mortazavi AGK, van Baren M, Salzberg S, Wold B, Pachter L: **Transcript assembly and quantification by RNA-Seq reveals unannotated transcripts and isoform switching during cell differentiation.** *Nature Biotechnology* 2010, **28**.

14. Conesa A, Gotz S, Garcia-Gomez JM, Terol J, Talon M, Robles M: **Blast2GO: a universal tool for annotation, visualization and analysis in functional genomics research.** *Bioinformatics* 2005, **21:**3674-3676.

15. Marchler-Bauer A, Derbyshire MK, Gonzales NR, Lu S, Chitsaz F, Geer LY, Geer RC, He J, Gwadz M, Hurwitz DI, et al: **CDD: NCBI's conserved domain database.** *Nucleic Acids Res* 2015, **43:**D222-226.

16. Tardif M, Atteia A, Specht M, Cogne G, Rolland N, Brugiere S, Hippler M, Ferro M, Bruley C, Peltier G, et al: **PredAlgo: a new subcellular localization prediction tool dedicated to green algae.** *Mol Biol Evol* 2012, **29:**3625-3639.

17. Schmollinger S, Mühlhaus T, Boyle NR, Blaby IK, Casero D, Mettler T, Moseley JL, Kropat J, Sommer F, Strenkert D: **Nitrogen-sparing mechanisms in Chlamydomonas affect the transcriptome, the proteome, and photosynthetic metabolism.** *The Plant Cell Online* 2014, **26:**1410-1435.

18. Li J, Han D, Wang D, Ning K, Jia J, Wei L, Jing X, Huang S, Chen J, Li Y: **Choreography of Transcriptomes and Lipidomes of Nannochloropsis Reveals the Mechanisms of Oil Synthesis in Microalgae.** *The Plant Cell Online* 2014, **26:**1645-1665.

19. Yang ZK, Niu YF, Ma YH, Xue J, Zhang MH, Yang WD, Liu JS, Lu SH, Guan Y, Li HY: **Molecular and cellular mechanisms of neutral lipid accumulation in diatom following nitrogen deprivation.** *Biotechnol Biofuels* 2013, **6:**67.

20. Rismani-Yazdi H, Haznedaroglu BZ, Hsin C, Peccia J: **Transcriptomic analysis of the oleaginous microalga Neochloris oleoabundans reveals metabolic insights into triacylglyceride accumulation.** *Biotechnol Biofuels* 2012, **5:**74.

21. Sun D, Zhu J, Fang L, Zhang X, Chow Y, Liu J: **De novo transcriptome profiling uncovers a drastic downregulation of photosynthesis upon nitrogen deprivation in the nonmodel green alga Botryosphaerella sudeticus.** *BMC genomics* 2013, **14:**715.

22. Vieler A, Wu G, Tsai C-H, Bullard B, Cornish AJ, Harvey C, Reca I-B, Thornburg C, Achawanantakun R, Buehl CJ: **Genome, functional gene annotation, and nuclear transformation of the heterokont oleaginous alga Nannochloropsis oceanica CCMP1779.** *PLoS genetics* 2012, **8:**e1003064.

23. Consortium E: **Standards, guidelines, and best practices for RNA-seq.** *V1 0* 2011**:**1-7.

24. Kovar JL, Zhang J, Funke RP, Weeks DP: **Molecular analysis of the acetolactate synthase gene of Chlamydomonas reinhardtii and development of a genetically engineered gene as a dominant selectable marker for genetic transformation.** *Plant J* 2002, **29:**109-117.

25. Lumbreras V, Stevens DR, Purton S: **Efficient foreign gene expression in Chlamydomonas reinhardtii mediated by an endogenous intron.** *The Plant Journal* 1998, **14:**441-447.

26. Eichler-Stahlberg A, Weisheit W, Ruecker O, Heitzer M: **Strategies to facilitate transgene expression in Chlamydomonas reinhardtii.** *Planta* 2009, **229:**873-883.

27. Berthold P, Schmitt R, Mages W: **An engineered Streptomyces hygroscopicus aph 7 ″gene mediates dominant resistance against hygromycin B in Chlamydomonas reinhardtii.** *Protist* 2002, **153:**401-412.

28. Dong B, Hu H-H, Li Z-F, Cheng R-Q, Meng D-M, Wang J, Fan Z-C: **A novel bicistronic expression system composed of the intraflagellar transport protein gene ift25 and FMDV 2A sequence directs robust nuclear gene expression in Chlamydomonas reinhardtii.** *Applied Microbiology and Biotechnology* 2017**:**1-19.

29. Labadorf A, Link A, Rogers MF, Thomas J, Reddy AS, Ben-Hur A: **Genome-wide analysis of alternative splicing in Chlamydomonas reinhardtii.** *BMC Genomics* 2010, **11:**114.

30. Sullivan GM, Feinn R: **Using Effect Size—or Why the P Value Is Not Enough.** *Journal of Graduate Medical Education* 2012, **4:**279-282.

31. Grishkevich V, Yanai I: **Gene length and expression level shape genomic novelties.** *Genome Research* 2014, **24:**1497-1503.

32. Ramsköld D, Wang ET, Burge CB, Sandberg R: **An Abundance of Ubiquitously Expressed Genes Revealed by Tissue Transcriptome Sequence Data.** *PLOS Computational Biology* 2009, **5:**e1000598.

33. Ribeiro AS, Häkkinen A, Lloyd-Price J: **Effects of gene length on the dynamics of gene expression.** *Computational Biology and Chemistry* 2012, **41:**1-9.

34. Mussgnug JH: **Genetic tools and techniques for Chlamydomonas reinhardtii.** *Applied Microbiology and Biotechnology* 2015, **99:**5407-5418.

35. Trapnell C, Williams BA, Pertea G, Mortazavi A, Kwan G, Van Baren MJ, Salzberg SL, Wold BJ, Pachter L: **Transcript assembly and quantification by RNA-Seq reveals unannotated transcripts and isoform switching during cell differentiation.** *Nature biotechnology* 2010, **28:**511-515.

36. Miller R, Wu G, Deshpande RR, Vieler A, Gärtner K, Li X, Moellering ER, Zäuner S, Cornish AJ, Liu B: **Changes in transcript abundance in Chlamydomonas reinhardtii following nitrogen deprivation predict diversion of metabolism.** *Plant physiology* 2010, **154:**1737-1752.

37. Juergens MT, Deshpande RR, Lucker BF, Park J-J, Wang H, Gargouri M, Holguin FO, Disbrow B, Schaub T, Skepper JN, et al: **The Regulation of Photosynthetic Structure and Function during Nitrogen Deprivation in Chlamydomonas reinhardtii.** *Plant Physiology* 2015, **167:**558-573.

38. Jin J, Tian F, Yang D-C, Meng Y-Q, Kong L, Luo J, Gao G: **PlantTFDB 4.0: toward a central hub for transcription factors and regulatory interactions in plants.** *Nucleic Acids Research* 2016.

39. Camargo A, Llamas Á, Schnell RA, Higuera JJ, González-Ballester D, Lefebvre PA, Fernández E, Galván A: **Nitrate Signaling by the Regulatory Gene NIT2 in Chlamydomonas.** *The Plant Cell* 2007, **19:**3491-3503.

40. Romero-Campero FJ, Perez-Hurtado I, Lucas-Reina E, Romero JM, Valverde F: **ChlamyNET: a Chlamydomonas gene co-expression network reveals global properties of the transcriptome and the early setup of key co-expression patterns in the green lineage.** *BMC Genomics* 2016, **17:**227.

41. Ngan CY, Wong C-H, Choi C, Yoshinaga Y, Louie K, Jia J, Chen C, Bowen B, Cheng H, Leonelli L, et al: **Lineage-specific chromatin signatures reveal a regulator of lipid metabolism in microalgae.** *Nature Plants* 2015, **1:**15107.

42. Boyle NR, Page MD, Liu B, Blaby IK, Casero D, Kropat J, Cokus SJ, Hong-Hermesdorf A, Shaw J, Karpowicz SJ: **Three acyltransferases and nitrogen-responsive regulator are implicated in nitrogen starvation-induced triacylglycerol accumulation in Chlamydomonas.** *Journal of Biological Chemistry* 2012, **287:**15811-15825.

43. Breuer G, de Jaeger L, Artus VP, Martens DE, Springer J, Draaisma RB, Eggink G, Wijffels RH, Lamers PP: **Superior triacylglycerol (TAG) accumulation in starchless mutants of Scenedesmus obliquus:(II) evaluation of TAG yield and productivity in controlled photobioreactors.** *Biotechnology for biofuels* 2014, **7:**1-11.

44. Jaeger Ld, Verbeek RE, Draaisma RB, Martens DE, Springer J, Eggink G, Wijffels RH: **Superior triacylglycerol (TAG) accumulation in starchless mutants of Scenedesmus obliquus:(I) mutant generation and characterization.** *Biotechnology for Biofuels* 2014, **7**.

45. Li Y, Han D, Hu G, Dauvillee D, Sommerfeld M, Ball S, Hu Q: **Chlamydomonas starchless mutant defective in ADP-glucose pyrophosphorylase hyper-accumulates triacylglycerol.** *Metab Eng* 2010, **12**.

46. Li Y, Han D, Hu G, Sommerfeld M, Hu Q: **Inhibition of starch synthesis results in overproduction of lipids in Chlamydomonas reinhardtii.** *Biotechnol Bioeng* 2010, **107**.

47. Siaut M, Cuiné S, Cagnon C, Fessler B, Nguyen M, Carrier P, Beyly A, Beisson F, Triantaphylidès C, Li-Beisson Y, Peltier G: **Oil accumulation in the model green alga Chlamydomonas reinhardtii: characterization, variability between common laboratory strains and relationship with starch reserves.** *BMC Biotechnology* 2011, **11:**1-15.

48. Davey MP, Horst I, Duong GH, Tomsett EV, Litvinenko AC, Howe CJ, Smith AG: **Triacylglyceride production and autophagous responses in Chlamydomonas reinhardtii depend on resource allocation and carbon source.** *Eukaryot Cell* 2014, **13:**392-400.

49. Work VH, Radakovits R, Jinkerson RE, Meuser JE, Elliott LG, Vinyard DJ, Laurens LM, Dismukes GC, Posewitz MC: **Increased lipid accumulation in the Chlamydomonas reinhardtii sta7-10 starchless isoamylase mutant and increased carbohydrate synthesis in complemented strains.** *Eukaryotic cell* 2010, **9:**1251-1261.

50. Blaby IK, Glaesener AG, Mettler T, Fitz-Gibbon ST, Gallaher SD, Liu B, Boyle NR, Kropat J, Stitt M, Johnson S, et al: **Systems-level analysis of nitrogen starvation-induced modifications of carbon metabolism in a Chlamydomonas reinhardtii starchless mutant.** *Plant Cell* 2013, **25:**4305-4323.

51. Schulz-Raffelt M, Chochois V, Auroy P, Cuine S, Billon E, Dauvillee D, Li-Beisson Y, Peltier G: **Hyper-accumulation of starch and oil in a Chlamydomonas mutant affected in a plant-specific DYRK kinase.** *Biotechnol Biofuels* 2016, **9:**55.

52. Krishnan A, Kumaraswamy GK, Vinyard DJ, Gu H, Ananyev G, Posewitz MC, Dismukes GC: **Metabolic and photosynthetic consequences of blocking starch biosynthesis in the green alga Chlamydomonas reinhardtii sta6 mutant.** *The Plant Journal* 2015, **81:**947-960.

53. Streb S, Zeeman SC: **Starch Metabolism in Arabidopsis.** *The Arabidopsis Book / American Society of Plant Biologists* 2012, **10:**e0160.

54. Satoh H, Shibahara K, Tokunaga T, Nishi A, Tasaki M, Hwang S-K, Okita TW, Kaneko N, Fujita N, Yoshida M, et al: **Mutation of the Plastidial α-Glucan Phosphorylase Gene in Rice Affects the Synthesis and Structure of Starch in the Endosperm.** *The Plant Cell* 2008, **20:**1833-1849.

55. Dauvillée D, Chochois V, Steup M, Haebel S, Eckermann N, Ritte G, Ral J-P, Colleoni C, Hicks G, Wattebled F, et al: **Plastidial phosphorylase is required for normal starch synthesis in Chlamydomonas reinhardtii.** *The Plant Journal* 2006, **48:**274-285.

56. Tunçay H, Findinier J, Duchêne T, Cogez V, Cousin C, Peltier G, Ball SG, Dauvillée D: **A Forward Genetic Approach in Chlamydomonas reinhardtii as a Strategy for Exploring Starch Catabolism.** *PLoS ONE* 2013, **8:**e74763.

57. Jang S, Yamaoka Y, Ko D-h, Kurita T, Kim K, Song W-Y, Hwang J-U, Kang B-H, Nishida I, Lee Y: **Characterization of a Chlamydomonas reinhardtii mutant defective in a maltose transporter.** *Journal of Plant Biology* 2015, **58:**344-351.

58. Lu YAN, Sharkey TD: **The importance of maltose in transitory starch breakdown.** *Plant, Cell & Environment* 2006, **29:**353-366.

59. Colleoni C, Dauvillée D, Mouille G, Buléon A, Gallant D, Bouchet B, Morell M, Samuel M, Delrue B, d'Hulst C, et al: **Genetic and Biochemical Evidence for the Involvement of α-1,4 Glucanotransferases in Amylopectin Synthesis.** *Plant Physiology* 1999, **120:**993-1004.

60. Alexa A, Rahnenfuhrer J: **topGO: Enrichment Analysis for Gene Ontology.** *R package version 2260* 2016.

61. Jaeger D, Hübner W, Huser T, Mussgnug JH, Kruse O: **Nuclear transformation and functional gene expression in the oleaginous microalga Monoraphidium neglectum.** *Journal of Biotechnology*, **2017**.

62. Hilker R, Stadermann KB, Schwengers O, Anisiforov E, Jaenicke S, Weisshaar B, Zimmermann T, Goesmann A: **ReadXplorer 2-detailed read mapping analysis and visualization from one single source.** *Bioinformatics* 2016.

63. Hilker R, Stadermann KB, Doppmeier D, Kalinowski J, Stoye J, Straube J, Winnebald J, Goesmann A: **ReadXplorer--visualization and analysis of mapped sequences.** *Bioinformatics* 2014, **30:**2247-2254.

64. Li W, Cowley A, Uludag M, Gur T, McWilliam H, Squizzato S, Park YM, Buso N, Lopez R: **The EMBL-EBI bioinformatics web and programmatic tools framework.** *Nucleic acids research* 2015, **43:**W580-W584.

65. Andrews S: **FastQC: a quality control tool for high throughput sequence data.** *Available online at:* [*http://wwwbioinformaticsbabrahamacuk/projects/fastqc*](http://wwwbioinformaticsbabrahamacuk/projects/fastqc) 2010.
